# Highly efficient homology‐directed repair using CRISPR/Cpf1‐geminiviral replicon in tomato

**DOI:** 10.1111/pbi.13373

**Published:** 2020-04-01

**Authors:** Tien Van Vu, Velu Sivankalyani, Eun‐Jung Kim, Duong Thi Hai Doan, Mil Thi Tran, Jihae Kim, Yeon Woo Sung, Minwoo Park, Yang Jae Kang, Jae‐Yean Kim

**Affiliations:** ^1^ Division of Applied Life Science (BK21 Plus Program) Plant Molecular Biology and Biotechnology Research Center Gyeongsang National University Jinju Korea; ^2^ National Key Laboratory for Plant Cell Biotechnology Agricultural Genetics Institute Bac Tu Liem Vietnam; ^3^ Hyundai Seed Co., LTD. Yeoju Korea; ^4^ Division of Life Science Gyeongsang National University Jinju Korea

**Keywords:** CRISPR/Cas9, CRISPR/Cpf1, gene targeting, genome editing, homology‐directed repair, multi‐replicon

## Abstract

Genome editing via the homology‐directed repair (HDR) pathway in somatic plant cells is very inefficient compared with error‐prone repair by nonhomologous end joining (NHEJ). Here, we increased HDR‐based genome editing efficiency approximately threefold compared with a Cas9‐based single‐replicon system via the use of *de novo* multi‐replicon systems equipped with CRISPR/LbCpf1 in tomato and obtained replicon‐free but stable HDR alleles. The efficiency of CRISPR/LbCpf1‐based HDR was significantly modulated by physical culture conditions such as temperature and light. Ten days of incubation at 31 °C under a light/dark cycle after *Agrobacterium*‐mediated transformation resulted in the best performance among the tested conditions. Furthermore, we developed our single‐replicon system into a multi‐replicon system that effectively increased HDR efficiency. Although this approach is still challenging, we showed the feasibility of HDR‐based genome editing of a salt‐tolerant SlHKT1;2 allele without genomic integration of antibiotic markers or any phenotypic selection. Self‐pollinated offspring plants carrying the HKT1;2 HDR allele showed stable inheritance and germination tolerance in the presence of 100 mm NaCl. Our work may pave the way for transgene‐free editing of alleles of interest in asexually and sexually reproducing plants.

## Introduction

In plant somatic cells, double‐strand DNA breaks (DSBs) are efficiently repaired by a nonhomologous end‐joining (NHEJ) mechanism, which dominates over the homology‐directed repair (HDR) pathway (Jiang *et al.*, 2013; Puchta, 2005). NHEJ repair usually leads to various types of mutations including DNA sequence insertions, deletions (Hsu *et al.*, 2014; Zetsche *et al.*, 2015), chromosome rearrangement or chromosome relocation (Ferguson and Alt, 2001; Richardson *et al.*, 1998; Varga and Aplan, 2005). Early in the 1990s, a transgenic approach using yeast mitochondrial I‐Sce I endonuclease as a DSB inducer was adopted in attempts to investigate the mechanisms of DSB repair in plants, especially gene targeting via the HDR pathway in plant somatic cells (Fauser *et al.*, 2012; Puchta *et al.*, 1993), which have been the main targets of recent plant genome engineering approaches (Baltes *et al.*, 2014; Belhaj *et al.*, 2013; Čermák *et al.*, 2015; Nekrasov *et al.*, 2013). In plant somatic cells, the HDR pathway employs homologous DNA templates to precisely repair damaged DNA, mainly via the synthesis‐dependent strand annealing (SDSA) mechanism, with an extremely low efficiency (Puchta *et al.*, 1996; Szostak *et al.*, 1983), leading to difficulties in practical applications. Therefore, research on plant gene targeting has continued to focus on improving HDR efficacy. Previously reported data have indicated two most important factors affecting HDR efficiency in plant somatic cells: DSB formation and the amount of homologous DNA templates available at sites of breakage (Baltes *et al.*, 2014; Endo *et al.*, 2016; Puchta, 2005; Puchta *et al.*, 1993; Townsend *et al.*, 2009).

The recent development of the clustered regularly interspaced short palindromic repeat (CRISPR)/CRISPR‐associated (Cas) protein system has provided excellent molecular scissors for the generation of DSBs. *Streptococcus pyogenes* Cas 9 (SpCas9) (Sapranauskas *et al.*, 2011) and *Lachnospiraceae bacterium* Cas12a (LbCas12a or LbCpf1) (Zetsche *et al.*, 2015) have been adapted for wide use in genome engineering studies in various kingdoms including Plantae (Barrangou and Doudna, 2016; Hsu *et al.*, 2014; Jinek *et al.*, 2012). The former system generally generates blunt ends (Jinek *et al.*, 2012) at DSBs, while the latter cuts in a cohesive end configuration (Zetsche *et al.*, 2015). As a consequence of DSB repair by NHEJ, the two types of CRISPR complexes exhibit comparably high indel mutation rates under *in vivo* conditions, thus proving to be ideal tools for DSB formation for initiating targeted HDR in plants. Furthermore, it has been suggested that the Cpf1 complex might present an advantage in HDR‐based genome editing compared with the Cas9 complex because the cutting site of Cpf1 is located distal to the core target sequence and the protospacer‐adjacent motif (PAM), potentially allowing recutting even after indel mutations are introduced during NHEJ‐mediated repair (Lowder *et al.*, 2016; Zetsche *et al.*, 2015). CRISPR/Cpf1 complexes were recently successfully applied for gene targeting in plants (Li *et al.*, 2018), providing alternative options for T‐rich target site selection.

Because of the highly efficient replication of geminivirus genomes and their single‐stranded DNA nature, these genomes have been used as an ideal DNA template carrier for gene targeting in plants. Geminiviral genomic DNAs have been reconstructed to exogenously overexpress foreign proteins in plants at up to 80‐fold higher levels compared with those of conventional T‐DNA systems (Mor *et al.*, 2003; Needham *et al.*, 1998; Zhang and Mason, 2006), due to their highly autonomous replication inside host nuclei and the ability to reprogramme cells (Gutierrez, 1999; Hanley‐Bowdoin *et al.*, 2013). Furthermore, Rep/RepA has been reported to promote a cell environment that is permissive for homologous recombination to stimulate the replication of viral DNA. Interestingly, it has been reported that somatic homologous recombination is promoted by geminiviral infection (Richter *et al.*, 2014). The above characteristics of geminiviral replicons have been shown to make them ideal delivery tools for introducing large amounts of homologous donor templates to plant nuclei. Likewise, a bean yellow dwarf virus (BeYDV)‐based replicon was developed by replacing its movement protein and coat protein genes with Cas9 or TALEN to improve gene targeting in plants (Baltes *et al.*, 2014; Butler *et al.*, 2016; Čermák *et al.*, 2015; Dahan‐Meir *et al.*, 2018; Gil‐Humanes *et al.*, 2017; Hummel *et al.*, 2018). The LbCpf1 complex, which was subsequently discovered and adapted for genome editing in 2015, has not been tested in combination with geminiviral replicon systems for plant gene targeting.

Despite higher success rates in gene targeting in plants using the geminiviral replicon system, most of the reported cases have required selection markers associated with the edited alleles, indicating that plant gene targeting without the use of selection markers is still challenging (Butler *et al.*, 2016; Gil‐Humanes *et al.*, 2017; Hummel *et al.*, 2018). In addition, the effective application of replicon cargos in plant gene targeting has been shown to be limited by their size (Baltes *et al.*, 2014; Suarez‐Lopez and Gutierrez, 1997). Therefore, plant gene targeting, especially in cases of selection marker‐free alleles, still requires improvement. Here, we report significant improvement of homology‐directed repair using CRISPR/LbCpf1‐geminiviral multi‐replicons in tomato and the successful application of the system to target a marker‐free salt‐tolerant HKT1;2 allele.

## Results and discussion

### The CRISPR/LbCpf1‐based geminiviral replicon system is feasible for performing HDR in tomato

**Figure 1:**
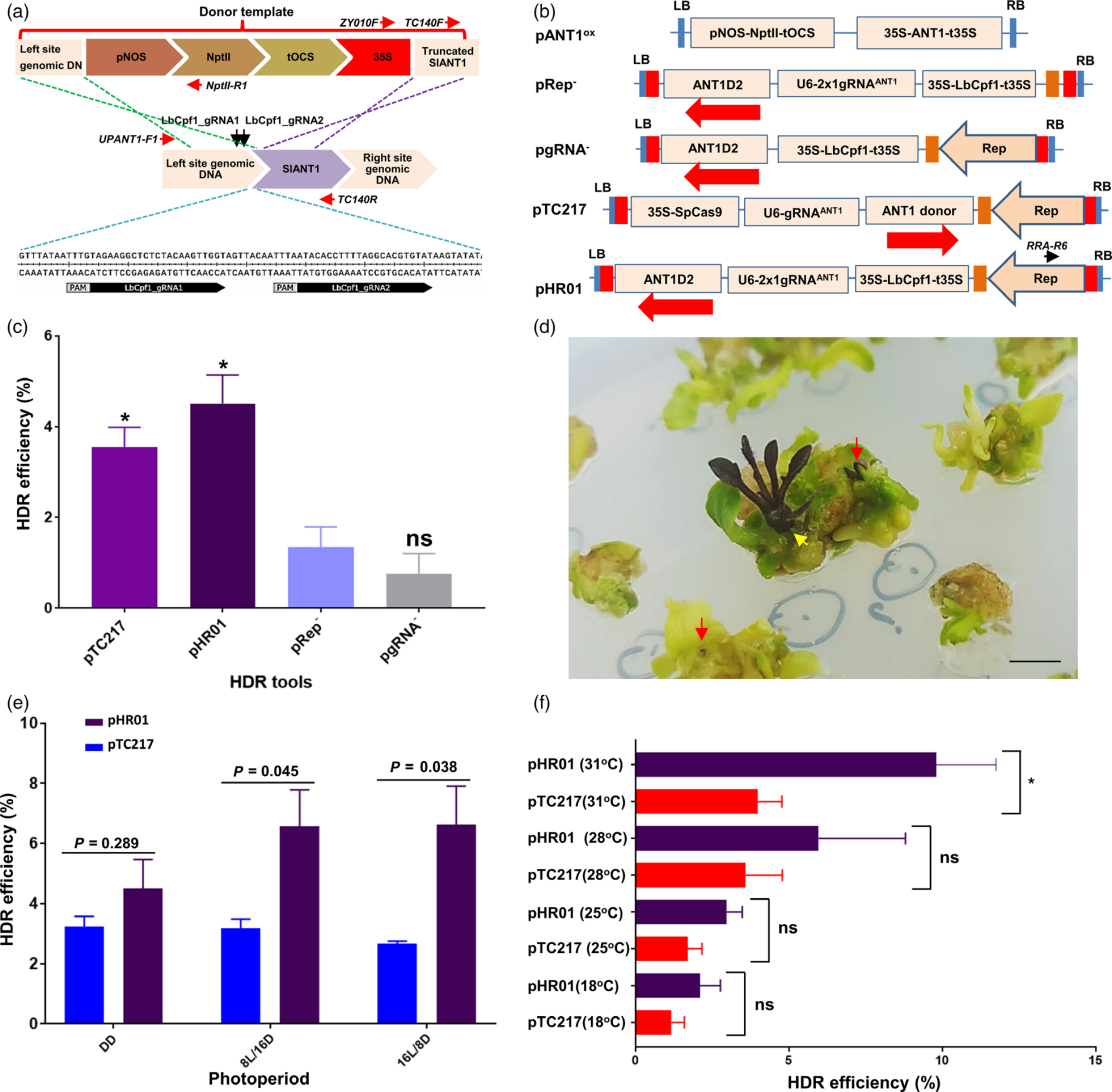
HDR‐based genome editing of the ANT1 locus. (a) Representatives of ANT1 targeting sites and homologous DNA donor template construction. The upstream sequence of the ANT1 locus (middle panel) was selected for targeting by HDR. The kanamycin expression cassette (pNOS‐NptII‐tOCS) and CaMV 35S promoter were designed to be inserted at a position 142 bp upstream of the ANT1 start codon. The cutting sites of the two guide RNAs used in this study are indicated by two black arrows. The sequences of the gRNAs are shown in the bottom panel. The red arrows show the relative binding sites and orientations of the primers used for analyses of HDR events. (b) T‐DNA constructs used for HDR improvement experiments. The dual‐guide RNA scaffold (2x1 gRNA^ANT1^) was driven by the *Arabidopsis* U6 promoter core element (75 bp). The LbCpf1 expression cassette was re‐engineered to contain the *Arabidopsis* Ubiquitin 1 intron I downstream of the CaMV 35S promoter and upstream of LbCpf1 and to be terminated by the CaMV 35S terminator (35S‐LbCpf1I‐t35S). Red and orange boxes indicate long intergenic regions (LIR) and short intergenic regions (SIR) of geminivirus DNA, respectively. The black arrow indicates the relevant binding site and orientation of the RRA‐R6 primer for subsequent analyses. The red arrows show the orientation of the ANT1 donor templates (ANT1D2). (c) Comparison of HDR efficiency between different constructs. Transformed tomato cotyledon fragments were incubated under continuous darkness at 28 °C for the first 10 days after washing. (d) Representative photographs of HDR‐edited T0 events indicated by purple calli (red arrows) or direct HDR shoot formation (yellow arrow). (e) Impact of photoperiod on HDR. The transformed tomato cotyledon fragments were incubated under different lighting regimes at 28 °C for the first 10 days after washing. DD: continuous darkness; 8 L/16 D: 8‐h light/16‐h darkness; 16 L/8 D: 16‐h light/8‐h darkness. (f) HDR efficiencies of the pTC217 and pHR01 constructs obtained at various temperatures. HDR efficiencies were recorded in at least triplicate and were calculated and plotted using PRISM 7.01 software (details of the statistical analyses are described in Methods section). *: significantly different (*P* < 0.05); ns: not significantly different; *P* values are shown on the top of the bars of (e) for comparison. The data in (c), (e) and (f) are represented as the mean ± SEM.

To test the hypothesis above, we re‐engineered a BeYDV replicon to supply a high dose of homologous donor templates and used the CRISPR/LbCpf1 system (Zetsche *et al.*, 2015) for DSB formation (Figure 1a and b). Two long intergenic regions (LIR) of BeYDV (pLSLR) (Baltes *et al.*, 2014) were cloned in the same orientation with a short intergenic region (SIR) inserted between them, generating an LIR‐SIR‐LIR amplicon unit (Data S1). To support the autonomous replication of the replicon, the Rep/RepA coding sequence was also introduced in *cis* (in the centre of the 3’ side, SIR‐LIR) and transcriptionally driven by the bidirectional promoter activity of the LIR. This cloning strategy interrupted a possible upstream ORF of Rep/RepA and added an AAA Kozak consensus sequence (Kozak, 1981) upstream of the major ATG of Rep (Figure S1A and B; Data S1), thus potentially contributing to increasing the translation of the Rep protein (Barbosa *et al.*, 2013; Zhang *et al.*, 2018). The selection of HDR events was performed with a double selection/screening system based on kanamycin resistance and anthocyanin overproduction (Figure 1a; Figure S2A and B; Data S1).

To validate our system, the LbCpf1 expression cassette driven by the CaMV 35S promoter and 5’UTR with AtUBI10 intron I (to suppress silencing effects (Christie *et al.*, 2011)), guide RNA scaffolds driven by the AtU6 promoter (Data S1) (Belhaj *et al.*, 2013) and donor templates were cloned into the *de novo*‐engineered geminiviral DNA replicon (Figure 1b) and transformed via *Agrobacterium*‐mediated transformation into tomato cotyledon explants. The *de novo*‐engineered geminiviral DNA replicon system exhibited efficient and durable maintenance of circularized DNAs in mature tomato leaves (Figure S3). The LbCpf1 system using two guide RNAs for targeting the ANT1 gene, a key transcription factor controlling the anthocyanin pathway, showed a much higher HDR efficiency, of 4.51 ± 0.63% (normalized to an overexpression construct (pANT1^ox^, Figure 1b)), than the other control constructs, including the ‘minus Rep’ (pRep^‐^) and ‘minus gRNA’ (pgRNA^‐^) constructs (Figure 1c; Table S1A). LbCpf1 system‐based HDR events were visualized by the presence of purple calli and/or shoots (Figure 1c and d), and its efficiency was similar to that of a CRISPR/SpCas9‐based construct (pTC217) (Čermák *et al.*, 2015) included in the same experiment (Figure 1c; Table S1A) or used in hexaploid wheat with the same scoring method (Gil‐Humanes *et al.*, 2017). It is worth noting that the normalized HDR efficiencies reported from this study (see Materials and Methods section) using transformed cell‐based efficiency are calculated differently from those reported in the initial work by Čermák *et al. *(2015). The data obtained from this experiment revealed that functional geminiviral replicons were crucial for increasing HDR efficiencies of the Cpf1 complex, as shown for Cas9 system (Čermák *et al.*, 2015). This result shows the feasibility of highly efficient HDR in plants using Cpf1 expressed from a geminiviral replicon, thus expanding the choices of molecular scissor system for gene targeting in plants.

### Favourable physical conditions significantly increase the HDR efficiency of the CRISPR/LbCpf1‐based geminiviral replicon system

In seeking suitable physical conditions for *Agrobacterium*‐mediated delivery and DSB repair using our HDR tool in tomato somatic cells, we investigated various incubation regimes at early stages post‐transformation. Short‐day conditions have been shown to have strong impacts on intrachromosomal recombination repair (ICR) in *Arabidopsis* (Boyko *et al.*, 2005). We tested whether the same could be true for the gene targeting approach in tomato. Using various lighting regimes, including complete darkness (DD), and short (8 h light/16 h dark; 8 L/16 D)‐day and long (16 L/8 D)‐day conditions, we found that the HDR efficiencies achieved under short‐ and long‐day conditions were higher than those under DD conditions in the case of LbCpf1 but not SpCas9 and reached 6.62 ± 1.29% (*P* < 0.05, Figure 1e; Table S1B). Considering the similar repair activities observed after DSBs were generated by either of the CRISPR/Cas systems, it was quite difficult to explain why the light conditions only affected LbCpf1‐based HDR in this experiment compared with the dark treatment. There must be unknown mechanism(s) that facilitate LbCpf1‐mediated HDR in a light‐dependent manner.

Temperature is an important factor controlling ICR (Boyko *et al.*, 2005), CRISPR/Cas9‐based targeted mutagenesis in plants (LeBlanc *et al.*, 2018) and CRISPR/Cpf1‐based HDR in zebrafish and *Xenopus* by controlling genome accessibility (Moreno‐Mateos *et al.*, 2017). In addition, Malzahn and co‐workers recently reported dependency of Cpf1 cleavage activity on temperature (Malzahn *et al.*, 2019). Pursuing the approach for the improvement of HDR, we compared the HDR efficiencies of the pHR01 and pTC217 systems subjected to various temperature treatments under an 8 L/16 D photoperiod, since the two nucleases (SpCas9 and LbCpf1) may respond differently. Our data revealed that within a temperature range of 19–31 °C, the somatic HDR efficiency increased with increasing temperature (Figure 1f; Table S1C). Notably, at 31 °C, LbCpf1 showed an HDR efficiency (9.80 ± 1.12%) that was more than twofold higher than that of SpCas9 (*P* < 0.05) and was nearly twice that of a similar system in hexaploid wheat (Gil‐Humanes *et al.*, 2017) and an LbCpf1‐based T‐DNA tool in rice (Li *et al.*, 2018). The results supported the principle of heat stress‐stimulated HDR in plants reported by Boyko *et al. *(2005). The ease of LbCpf1 at genome accessibility at high temperatures (Moreno‐Mateos *et al.*, 2017) in combination with the ability to repeatedly cut at the target sites (Zetsche *et al.*, 2015) may explain the higher HDR efficiency of LbCpf1 than that of SpCas9. The claims are supported by the pHR01 and pTC217 guide RNA activity analyses. The number of transformed events carrying indel mutations was almost similar, but the average adjusted levels of mutation rate obtained from the pHR01‐based LbCpf1 (Table S2B; Data S2) were nearly threefold higher than those of the SpCas9‐based pTC217 events (Table S2A; Data S2). Interestingly, the LbCpf1 complex was shown to be highly active only at high temperatures (i.e. more than 29 °C) (Malzahn *et al.*, 2019), which partially explains the higher HDR efficiencies observed at high temperatures in this experiment. It is notable that a highly efficient CRISPR/LbCpf1 mutant in low temperature was reported for plant gene editing (Schindele and Puchta, 2019). Even the LbCpf1_gRNA1 appeared to be highly active at the on‐target site; no modification at two potential off‐targeting sites was observed (Data S3). Briefly, a comparison of data on plant HDR between Cas9‐ and Cpf1‐based systems at different temperatures and under short‐day conditions is presented to reveal the best conditions for plant HDR improvement.

### A multi‐replicon system outperformed the single‐replicon system in HDR‐based GE

**Figure 2:**
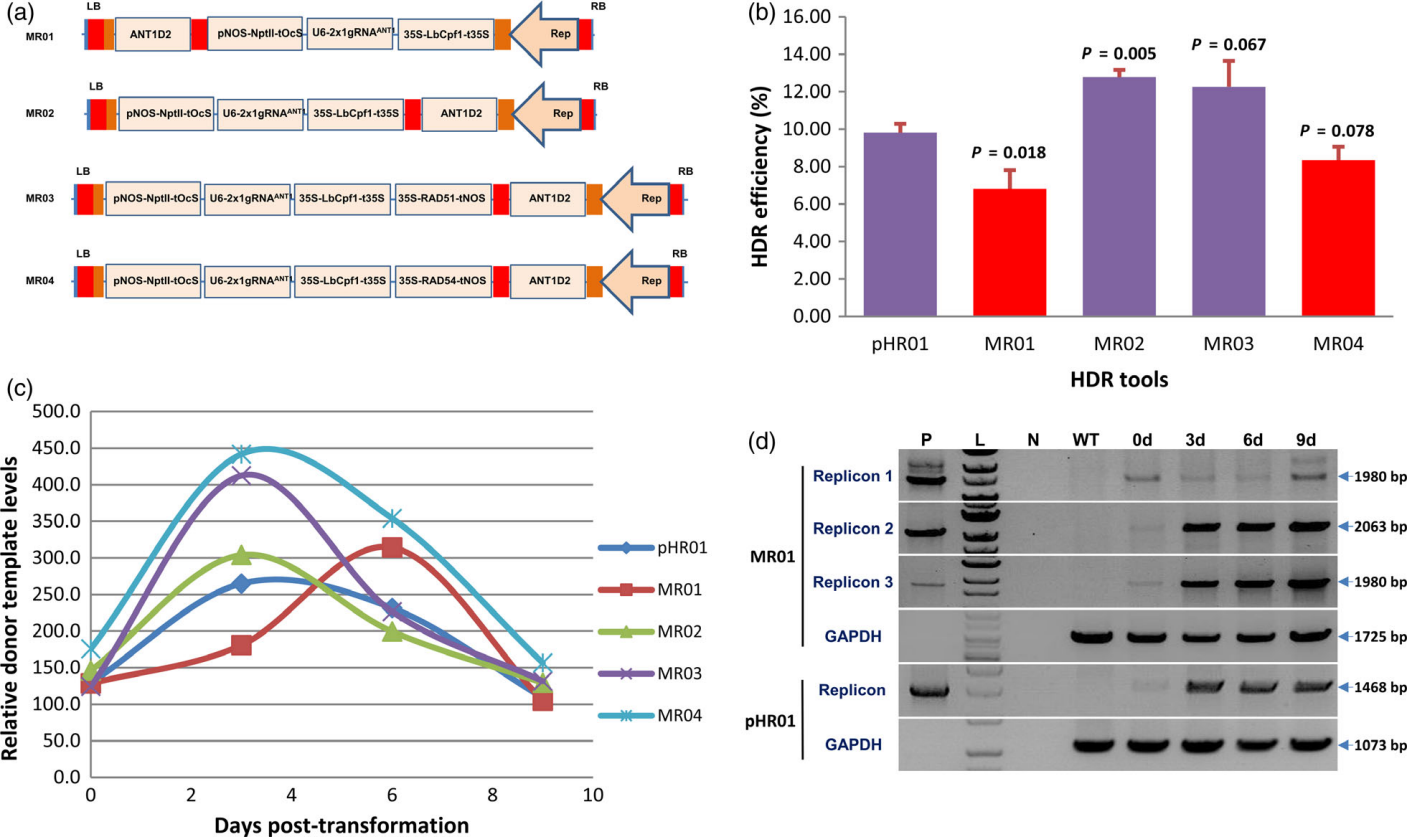
Multi‐replicon tools for HDR improvement. (a) Multi‐replicon constructs tested for the improvement of HDR over NHEJ. Red and orange boxes indicate LIRs and SIRs of geminiviral DNA, respectively. ANT1D2, ANT1 donor templates. (b) HDR efficiencies obtained using multi‐replicons as cargos for the HDR tools. HDR efficiencies were recorded four times and were calculated and plotted using PRISM 7.01 software (details of the statistical analyses are described in Materials and Methods section). *P* values (pairwise comparisons to pHR01 using Student’s test) are shown on the top of the bars. Data are represented as the mean ± SEM. (c) Relatively quantified donor template levels at different time points post‐transformation by qPCR using ANT1D2 template‐specific primers normalized to SlPDS. (d) PCR detection of circularized replicons simultaneously released from the MR01 vector. 0d, 3d, 6d and 9d: samples collected at 0, 3, 6 and 9 days post‐transformation with *Agrobacterium* carrying MR01. P: pHR01 plasmid isolated from Agrobacteria; L: 1 kb ladder; N: water control; WT: wild‐type tomato Hongkwang. The primer pairs used in PCR to detect circularized replicons are shown in Figure S4, bottom panel, and Table S4.

The size of viral replicons has been shown to be inversely correlated with their copy numbers (Baltes *et al.*, 2014; Suarez‐Lopez and Gutierrez, 1997). In an approach to overcome the replicon size limitation, we designed and tested the novel idea of using a T‐DNA system that potentially produces multiple replicons (Figure 2a, and Figure S4). Compared with pHR01, a multi‐replicon system designed to release donor templates from replicon 2 (MR02) but not replicon 1 (MR01) showed a significant increase in the HDR efficiency by 30% and reached up to 12.79 ± 0.37% (Figure 2b and Table S3). Temporal evaluation of donor template levels between the HDR tools showed significantly higher levels of MR02 at 3 days post‐transformation (dpt) compared with those of pHR01 and MR01 (Figure 2c). The highest donor template levels in multi‐replicons tested were available, while CRISPR/Cas was generating DSBs at early times after transformation (3 dpt, Figure 2c), except for MR01 showing a peak at 6 dpt (Figure 2c). Under the same conditions and calculation methods, the combination of our multi‐replicons with LbCpf1 significantly increased HDR efficiencies by threefold to fourfold compared with those of the Cas9‐based replicon systems. We also confirmed the release of three circularized replicons from the single vector used in this work (Figure 2d) by PCR amplification using circularized replicon‐specific primers (Table S4).

In another test of the multi‐replicon system, we overexpressed two key proteins involved in the plant HDR pathway from the replicon 1 site. Either SlRAD51 (Solyc07g017540.2) or SlRAD54 (Solyc04g056400.2) was overexpressed with the multi‐replicon tools (MR03 and MR04) (Figure 2a; Data S1). Surprisingly, even when the donor template level of MR03 or MR04 was nearly twice that of MR01 (Figure 2c), the HDR efficiency was not significantly different in the case of MR03 and was even significantly lower for MR04 (Figure 2b and Table S3). The assessment of mRNA levels of SlRAD51 (Figure S5A; Data S4) or SlRAD54 (Figure S5B; Data S4) of transformed events of MR03 or MR04, respectively, showed higher relative transcript levels (up to 522.18‐fold of SlRAD51 and 83.68‐fold of SlRAD54 transcripts) than multi‐replicon control (MR02) (Figure S5; Data S4). Overexpression of SlRAD54 might increase the displacement of SlRAD51 from SlRAD51‐bound dsDNAs at the early stage of HDR initiation (Petukhova *et al.*, 1999), thereby suppressing HDR to some extent in the case of MR04 (Figure 2b). Overexpression of either SlRAD51 (MR03) or SlRAD54 (MR04) increased the 3‐day peaks of geminiviral replicons (replicon 2 and 3) at 30%–50% compared with the control (MR02) (Figure 2c), confirming the positive roles of these proteins in geminivirus replication in a homologous recombination manner, as reported elsewhere (Kaliappan *et al.*, 2012; Richter *et al.*, 2016; Suyal *et al.*, 2013). The data also revealed a temporal difference in the maximal peaks of replicon 1 and 2 because replicon 1 was not accompanied by a Rep/RepA expression cassette.

The multi‐replicon system may provide more flexible choices for expressing multiple donor templates/genes/genetic tools in plant cells with temporally controllable copy levels without incurring an expression penalty from excess replicon sizes up to 18 kb (size of replicon 3 released by MR03). The validation of the multi‐replicon system provides an excellent alternative for genetic engineering in plants in addition to applications in plant genome editing. If we carefully design and clone multiple donor templates or gene expression cassettes into the multi‐replicons, we can control donor templates/gene doses without incurring penalties from excessing replicon size limitations.

### True ANT1 HDR events occurred at high frequency

**Figure 3:**
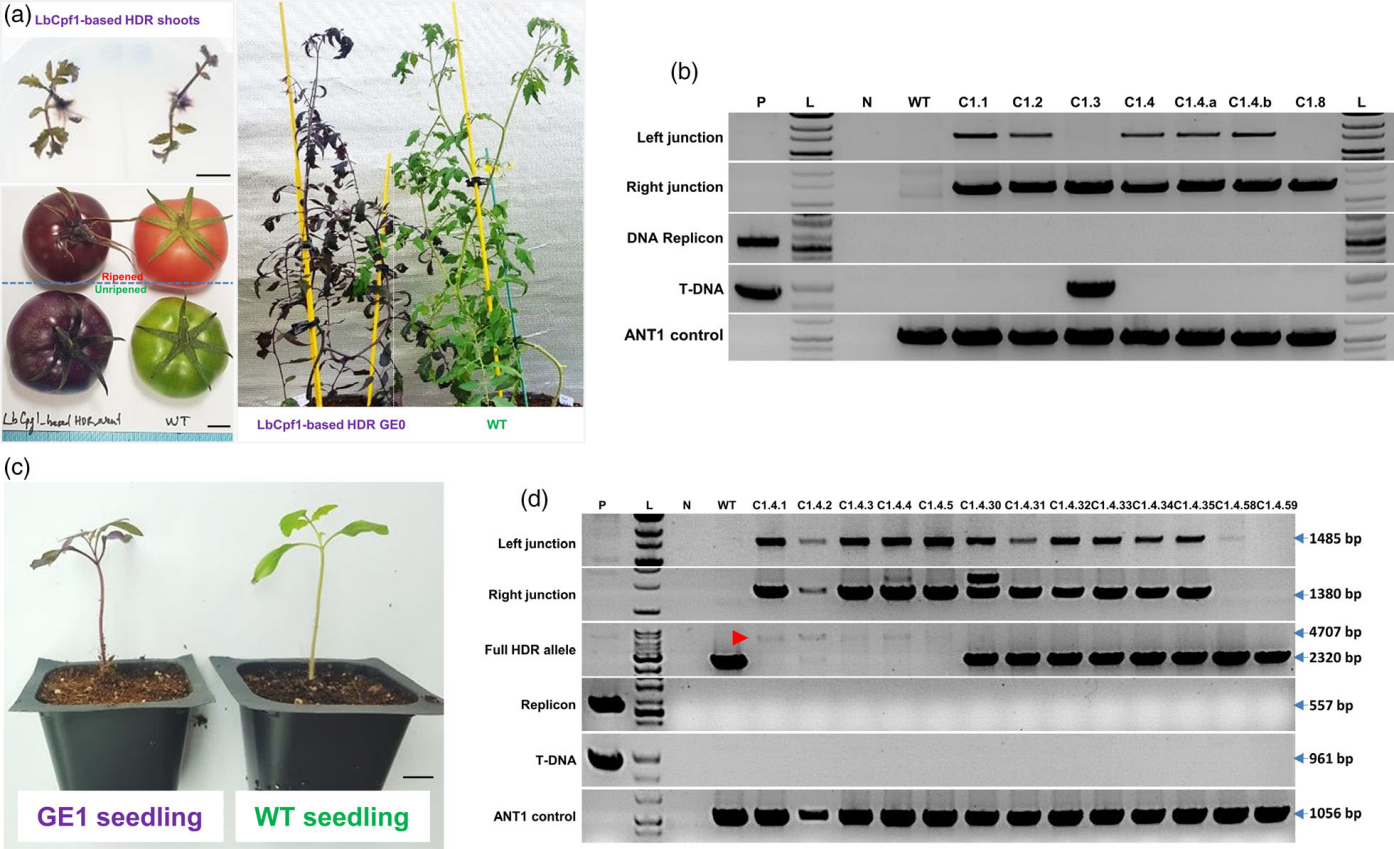
Analyses of HDR‐edited plants. (a) Representative HDR‐edited plants in greenhouse conditions and their fruits. Scale bars = 1 cm. (b) PCR analysis data of representative HDR‐independent events. P: pHR01 plasmid isolated from Agrobacteria; L: 1 kb ladder; N: water control; WT: wild‐type tomato Hongkwang; C1.1, C1.2, C1.3, C1.8: independent LbCpf1‐based HDR GE0 events. ANT1 control products were PCR‐amplified using the TC140F and TC140R primers (Figure 1a) flanking the upstream region of the ANT1 gene. (c) Generation 1 of HDR‐edited events (GE1). GE1 plants (left) germinated in soil in pots in comparison with wild‐type plants (right). Scale bar = 1 cm. (d) PCR analysis data of GE1 offspring resulting from C1.4 events. P: pHR01 plasmid isolated from Agrobacteria; L: 1 kb ladder; N: water control; WT: wild‐type tomato Hongkwang; C1.4.1, C1.4.2, C1.4.3, C1.4.4 and C1.4.5: GE1 plants showing dark purple colour obtained from the self‐pollination of plants from the C1.4 event. ANT1 control products were PCR‐amplified using the TC140F and TC140R primers (Figure 1a) flanking the upstream region of the ANT1 gene. Red arrowhead indicates HDR allele.

An important step in plant genome editing is the regeneration of edited calli into shoots. We used kanamycin in our study to select edited calli and plants. Since we put a fully functional NptII expression cassette into the ANT1 donor, we observed many WT‐like calli and green shoots arose from our plates. In the case, the purple marker was so much useful for us to select HDR events. Our observation recorded a significantly higher number of both purple spots per cotyledon and purple plants per cotyledon obtained from pHR01 and MR02 than those of pTC217 (Table S5). However, the regeneration of the purple calli into plants was not completely proportional probably due to pleiotropic impacts of the new replicon systems. To verify HDR repair events, PCR analyses were conducted using primers specific for the left (UPANT1‐F1/NptII‐R1) and right (ZY010F/TC140R) (Figure 1a; Tables S6 and S7) junctions employing genomic DNAs extracted from derived HDR events (independently regenerated purple plants or genome‐edited generation 0 (GE0)) (Figure 3a, Figures S6 and S7). For pHR01, all (16/16) of the analysed independent events showed the expected band for right junction integration, and 10 of 16 independent events showed the expected band for left junction repair (Figure 3b). The PCR products were sequenced to identify junction sequences. A majority of the events (11/16) showed sequences corresponding to perfect right arm integration through HDR repair, and 5 of 16 events showed a combination of HDR and NHEJ repair with an NHEJ fingerprint at the 5′ terminus of the pNOS sequence (Figure S7A, with event C1.8 highlighted in blue) or even an integration of the right board of T‐DNA at the left junction boundary (Figure S8). All of the sequences amplified from the left junctions showed perfected DNA sequence exchange via the HDR pathway (Figure S7B). The results obtained in these analyses revealed the common features of products repaired via HDR pathways in plant somatic cells reported elsewhere in dicots (Butler *et al.*, 2016; Čermák *et al.*, 2015; Dahan‐Meir *et al.*, 2018) and monocots (Gil‐Humanes *et al.*, 2017; Li *et al.*, 2018), regardless of whether a T‐DNA or geminiviral replicon system was involved. More importantly, 15 of 16 events showed no amplification of circularized forms of the DNA replicon, and even the replicon‐carrying events lost this replicon after long‐term growth in greenhouse conditions (data not shown), indicating that these plants were free of the replicon (Figure 3b). The absence of the replicon might be hypothetically explained by reverse construction of the donor template (Figure 1b), leading to the opposite arrangement of the LIR forward promoter sequence against a 35S promoter sequence (LIR‐p35S orientation interference), which triggers a silencing mechanism in plant cells in later stages. This possibility was later supported by the appearance of replicons in the majority of plants regenerated using other replicon systems without LIR‐p35S orientation interference.

### The HDR allele was stably inherited in offspring by self‐pollination and **backcrossing**

To validate stable heritable edits, we grew genome‐edited generation 1 (GE1) plants (Figure 3c) obtained from the self‐pollination of LbCpf1‐based HDR GE0 events and identified a segregating population with a purple phenotype (Table S8) similar to the segregating profiles shown by Čermák *et al. *(2015). PCR analyses of the segregating plants showed inheritance of the edited allele (Figure 3d and Figure S9). In addition, all GE1 plants tested showed no amplification of the DNA replicon (Figure 3d), indicating that the GE1 plants were also free of the replicons. The offspring segregated from the #C1.4 event were analysed in detail. Five dark purple plants (C1.4.1‐C1.4.5, homozygous for the ANT1 HDR‐edited allele; Figure S10), six pale purple plants (C1.4.30‐C1.4.35, heterozygous for the ANT1 HDR‐edited allele; Figure S10) and two wild‐type‐like plants did not contain the HDR‐edited allele, as expected (Figure 3d, predicted results correlated with phenotypes). The dark purple plants showed PCR amplification from the replaced allele but no amplification of the wild‐type allele when PCR was performed using primers flanking the editing site (Figure 1a). In contrast, heterozygous and wild‐type plants showed a band corresponding to the wild‐type allele. Further assessment indicated that the GE2 offspring of the homozygous GE1 plants were all dark purple, and the backcrossed (to WT female as pollen acceptors) BC1F1 generation all showed the pale purple phenotype (Figure S10), suggesting the feasibility of recovering the parental genetic background via backcrossing in cases of unexpected modification, including off‐target effects. Sanger sequencing revealed perfect inheritance of the HDR‐edited allele from the GE0 generation of event C1.4 (Figure S11) to its homozygous offspring. We subsequently subjected gDNAs of several putative homozygous and heterozygous GE1 lines to Southern blot analysis. The data revealed and confirmed the existence and inheritance of the edited locus in GE1 lines as shown at expected sizes at single HDR band (homozygote) or in combination of HDR and WT bands (heterozygote) (Figure S12; Data S5). Further, some GE1 lines (C1.4.4 and C1.9.1) presented a mixture of the precisely edited allele and others including the ones with one‐side HDR (one‐side right HDR or one‐side left HDR) and the other side repaired by illegitimate recombination (Figure S12). In addition, we detected unexpected bands in lines C1.9.1, C9.1.1 and C1.12.3 (Figure S12), which might be caused by unspecific genomic integration of partial replicon DNA.

### HDR‐based GE using allele‐associated marker‐free approaches

**Figure 4:**
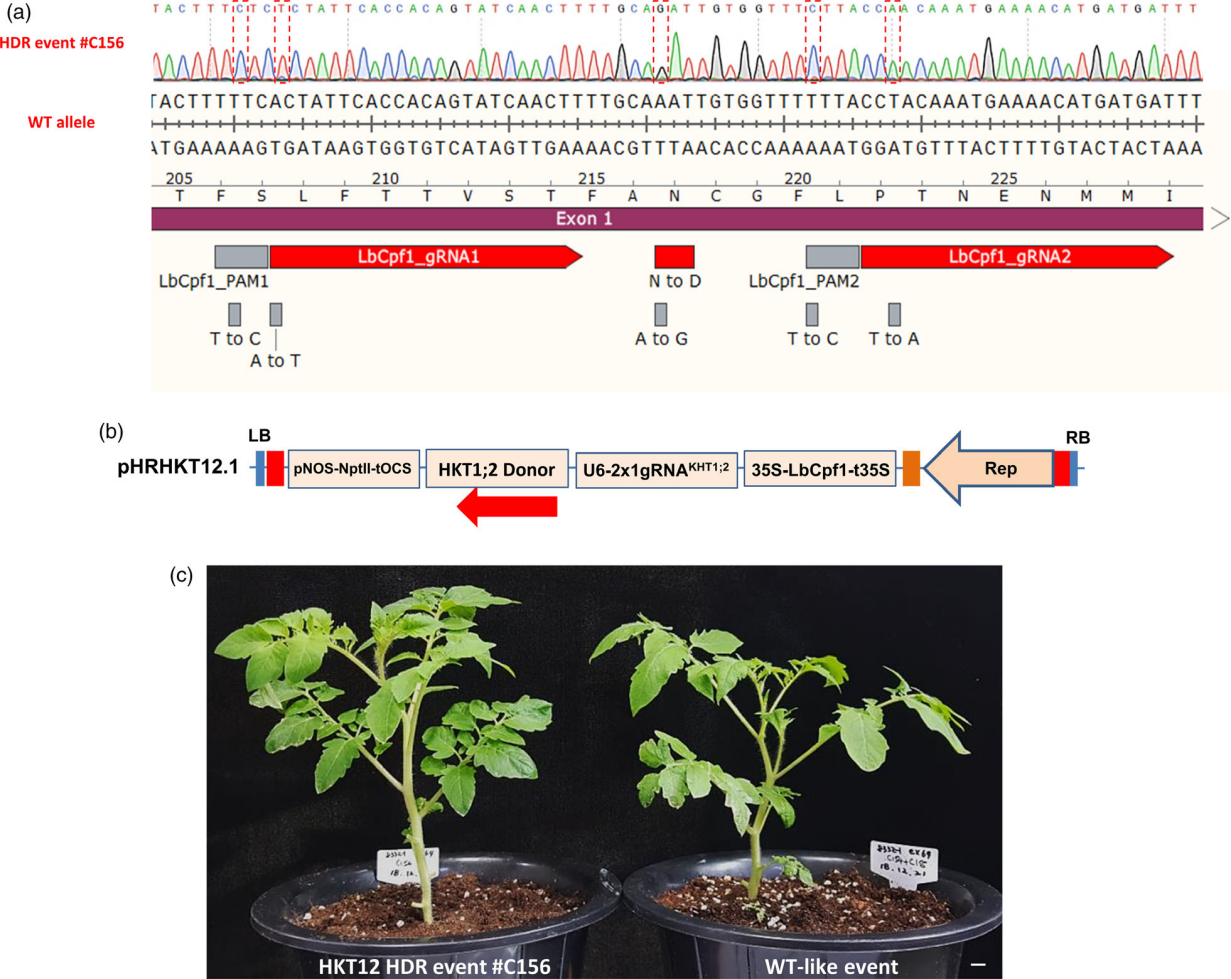
HKT1;2^N217D^ allele editing by HDR using the CRISPR/Cpf1‐based replicon system. (a) Sanger sequencing of event #C156. Sequence alignment shows the perfectly edited HKT1;2 N217 to D217 allele with the WT allele as a reference. The nucleotides highlighted in the discontinuous red boxes correspond to intended modifications for N217D, PAM and core sequences (to avoid recutting). (b) HDR construct layout for HKT1;2 editing. There is neither selection nor a visible marker integrated into the donor sequence. The *Npt*II marker was used for the enrichment of transformed cells. (c) Morphology of the HKT1;2^N217D^ edited event compared with its WT‐like event in greenhouse conditions. Scale bar = 1 cm.

To show the applicability of our HDR system to practical plant genome editing, we sought to use it to edit a potentially agronomic trait, and salinity tolerance was chosen as the target trait. High‐affinity K^+^ transporter 1;2 (HKT1;2) plays an important role in the maintenance of K + uptake under salt stress (Ali *et al.*, 2012). Salinity tolerance was determined by a single N/D variant (N217D in tomato) in the pore region of HKT1;2, which determines selectivity for Na + and K+ (Ali *et al.*, 2016). We succeeded in generating a heterozygous but perfect HDR GE0 event to produce the salt‐tolerant allele (N217D) (Ali *et al.*, 2016) (Figure 4a, Table S9) according to the analysis of 150 events (~0.66%) using our system with a *HKT1;2* gene donor template that included neither an allele‐associated antibiotic selection marker nor an ANT1 colour marker (Figure 4b; Data S1). The CRISPR/LbCpf1 system was very effective for NHEJ repair because it generated indel mutation rates of up to 72% in multiple mutation patterns decomposed by ICE–Synthego software (Hsiau *et al.*, 2019) (Figure S13A and B), in which most of the events resulted in 47%–97% cells carrying indel sequences (Table S10). In comparison with the first report on the allele‐associated marker‐free gene targeting of the CRTISO allele (Dahan‐Meir *et al.*, 2018), the HDR frequency obtained with the HKT1;2 locus in this study was much lower, possibly due to (i) lower cutting activity (note the indel mutation rates in Table S10), (ii) a different target site context or (iii) the use of a different strategy to express Rep/RepA (Dahan‐Meir and co‐workers used a replicon tool with Rep expression driven by a CaMV35S promoter from outside of LIR‐SIR‐LIR boundary), or unknown reasons associated with the CRTISO alleles, as claimed by the authors, or all of the above‐mentioned factors. We used a similar replicon tool to that reported by Dahan‐Meir *et al. *(2018) for ANT1 targeting via the HDR pathway in this study but obtained significantly lower HDR efficiencies than were obtained with the pHR01 tool (data not shown).

**Figure 5:**
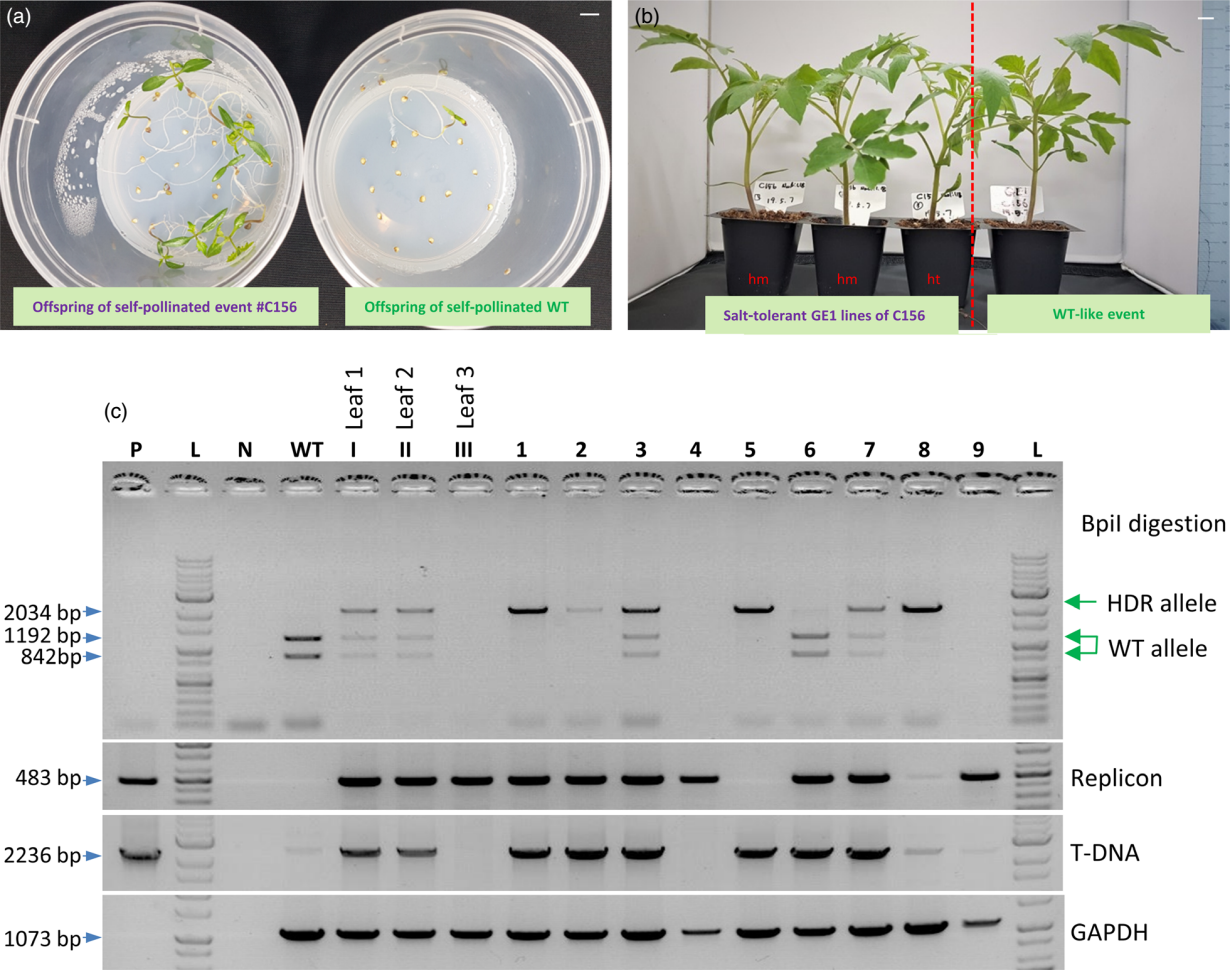
Evaluation of the GE1 offspring of the HKT1;2^N217D^ HDR event. (a) Salinity tolerance test at the germination stage using NaCl. Left panel: GE1 plants obtained from self‐pollination of the plants obtained from event #C156; right panel: WT control. Bar = 1 cm. (b) Salt‐tolerant plants (left panel) growing in soil showed normal growth compared with WT‐like event (right panel). hm = homozygous for the HKT1;2^N211D^ allele; ht = heterozygous for the HKT1;2^N217D^ allele. Bar = 1 cm. (c) Screening for the presence of HDR allele(s) in the tested plants via the cleaved amplified polymorphic sequence (CAPS) method. PCR amplification using primers flanking the targeted region was conducted. The PCR products were digested with the BpiI enzyme and resolved in a 1% agarose gel. P: plasmid control; L: 1 kb ladder; WT: wild‐type sample; Leaf 1, Leaf 2 and Leaf 3: samples collected from three different positions (angles) on the C156 plants. 1–9: GE1 plants of C156.

The editing event involving the D217 allele resulted in a normal morphology (Figure 4c) and normally set fruits (Figure S14) compared with WT. It should be noted that the mutated nucleotide (A to G) of *HKT1;2* is not accessible by any currently known base editor (BE), including xCas9‐ABE (Hu *et al.*, 2018), highlighting the significance of HDR‐based genome editing. We tested the self‐pollinated GE1 generation of the plants obtained from the event and observed up to 100 mm NaCl tolerance at the germination stage (Figure 5a) in both homozygous and heterozygous plants. The salt‐tolerant plants showed a 3‐ to 4‐day delay in germination compared with the mock controls but grew normally in NaCl‐containing medium (Figure 5a) and later fully recovered in soil (Figure 5b). Screening for the presence of HDR allele(s) in the tested plants via the cleaved amplified polymorphic sequence (CAPS) method showed allele segregation following Mendelian rules (Figure 5c). The true HKT1;2^N217D^ HDR alleles in the GE1 plants were ultimately confirmed by Sanger sequencing. It is worth noting that most of the elite alleles in plants do not associate with any selection marker, and hence, a highly efficient HDR with allele‐associated marker‐free system is in high demand.

Thus, through the application of various approaches, our study showed a significant improvement of HDR efficiency in tomato somatic cells. The HDR allele was stably inherited in subsequent generations obtained via self‐pollination and backcrossing. The advancement of HDR in somatic cells, the generation of replicon‐free HDR‐edited plants in the GE0 generation and the invention of multi‐replicon system open the door for practical applications of the technique to improve crop traits, with special interest for asexually reproducing crops.

## Experimental procedures

### Construction and cloning of HDR testing systems

The entire design principle and all cloning procedures followed MoClo (Weber *et al.*, 2011) and Golden Gate (Engler *et al.*, 2014) protocols. pLSL.R.Ly was designed by amplifying the long intergenic region (LIR), short intergenic region (SIR) and lycopene marker from the pLSLR plasmid (Čermák *et al.*, 2015) and was cloned following the order shown in Figure S3A and Data S1. Level 2 Golden Gate BpiI restriction sites flanking the pink marker gene (lycopene) were also integrated within the replicon for the cloning of HDR expression cassettes. The release of circularized DNA replicons was validated in tomato leaves (Figure S3B) and tomato cotyledon explants (data not shown). The pTC147 and pTC217 plasmids (Čermák *et al.*, 2015) were obtained from Addgene and used as a reference. The LbCpf1‐based HDR replicons were designed and cloned similar to the SpCas9‐based constructs, with two guide RNAs (LbCpf1_gRNA1 and LbCpf1_gRNA2; Figure 1a; Data S1). Donor DNAs (ANT1D2) were constructed for the integration of an antibiotic selection marker (NptII) and the insertion of a CaMV 35S promoter to drive overexpression of the ANT1 gene (pANT1^ox^; Figure 1a; Data S1). The dual‐guide RNA construct was designed by multiplexing the LbCpf1 crRNAs as a tandem repeat of scaffold RNA followed by 23‐nt guide RNA sequences. The crRNAs were driven by an AtU6 promoter (Kamoun Lab, Addgene #46968) and terminated by 7‐T chain sequences (Data S1).

### Tomato transformation

Our study of HDR improvement was conducted using tomato (Hongkwang cultivar, a local variety) as a model plant. All the binary vectors were transformed into *Agrobacterium tumefaciens* GV3101 (pMP90) using electroporation. *Agrobacterium*‐mediated transformation was used to deliver editing tools to tomato cotyledon fragments (Figure S15). Explants for transformation were prepared from 7‐day‐old cotyledons. Sterilized seeds of the Hongkwang cultivar were grown in MSO medium (half‐strength MS medium containing 30 g/L of sucrose, pH 5.8) at 25 ± 2 °C under 16‐h/8‐h light/dark conditions. Seven‐day‐old seedlings were collected, and their cotyledonary leaves were sliced into 0.2‐ to 0.3‐cm fragments. The fragments (explants) were pretreated in PREMC medium [MS basal salts, Gamborg B5 vitamins, 2.0 mg/L of Zeatin trans isomer and 0.2 mg/L of indolyl acetic acid (IAA), 1 mm of putrescine and 30 g/L of glucose, pH 5.7] for 1 day. The precultured explants were then pricked and transformed using *A. tumefaciens* GV3101::pMP90 cells carrying HR construct(s).

*Agrobacterium tumefaciens* GV3101::pMP90 cells were grown in primary culture overnight (LB containing suitable antibiotics) in a shaking incubator at 30 °C. Agrobacteria were then collected from the culture (OD 0.6–0.8) by centrifugation. The cells were resuspended in liquid ABM‐MS (pH 5.2) and 200 µm acetosyringone. Transformation was carried out for 25 min at RT. The explants were then transferred to cocultivation medium containing all of the components in the ABM‐MS medium and 200 µm acetosyringone, pH 5.8. The cocultivation plates were kept in the darkness at 25 °C for 2 days, and the explants were then shifted to nonselection medium (NSEL) for 5 days and subcultured in selection medium (SEL5). The nonselection and selection media contained all of the components of the preculture medium, and 300 mg/L of timentin and 80 mg/L of kanamycin. Subculture of the explants was carried out at 14‐day intervals to achieve the best regeneration efficiency. Explants containing purple calli or shoots were then transferred to SEL5R medium (similar to SEL5 but with the zeatin trans isomer concentration reduced to 1.0 mg/L) for further regeneration and/or elongation. When the shoots were sufficiently long (1.5–3.0 cm), they were transferred to rooting medium (containing all of the components of the elongation medium except the zeatin trans isomer plus 1.0 mg/L IBA) to generate intact plants. The intact plants from the rooting medium were transferred to vermiculite pots to allow them to harden before shifting them to soil pots in a greenhouse with a temperature of 26 ± 2 °C under a 16‐h/8‐h photoperiod. The experimental treatment of the physical conditions and data collection were conducted as described in Figure S15.

### HDR efficiency calculation

In a previous report, the HDR efficiency calculated by dividing the number of explants containing at least one purple callus (appearing as a purple spot) by the total number of explants obtained from *Agrobacterium*‐mediated transformation reached 12% with the replicon system (Čermák *et al.*, 2015). In the present study, purple spots were scored at 21 day post‐transformation and HDR efficiencies were calculated differently by normalization of the purple spot numbers per cotyledon explant obtained using genome editing constructs to the purple spot numbers per cotyledon explant counted in case of transformation of the SlANT1 overexpression cassette (pTC147 and pANT1^ox^; Figure 1b) in the same conditions.

### Plant genomic DNA isolation

Tomato genomic DNA isolation was performed using the DNeasy Plant Mini Kit (Qiagen, Hilden, Germany) according to the manufacturer’s protocol. Approximately 200 mg of leaf tissue was crushed in liquid nitrogen using a ceramic mortar and pestle and processed with the kit. Genomic DNA was eluted from the mini spin column with 50–80 µL of TE or nuclease‐free water.

### HDR event evaluation

The assessment of gene targeting junctions was performed by conventional PCR using primers flanking the left (UPANT1‐F1/NptII‐R1) and right (ZY010F/TC140R (Čermák *et al.*, 2015) (Tables S6 and S7) junctions and a high‐fidelity Taq DNA polymerase (Phusion Taq, Thermo Fisher Scientific, Waltham, MA) and Sanger sequencing (Solgent, Daejeon, Korea). DNA amplicons and related donor template levels were evaluated by semiquantitative PCR and qPCR (using KAPA SYBR FAST qPCR Kits, Sigma‐Aldrich, St. Louis, MS), respectively, using primers specific to only circularized replicons and the donor template. Additionally, the qPCR assays were designed and conducted following MIQE’s guidelines, with SlPDS (Solyc03g123760**)** and SlEF1 (Solyc07g016150**)** as normalized controls. Analyses of the inherited behaviour of the HDR‐edited allele were performed with genome‐edited generation 1 (GE1) by PCR and Sanger sequencing. Circularized replicons were detected using PCR with the corresponding primers for pHR01 (Table S6), multi‐replicons (Table S4) or pTC217 (Table S7).

### Statistical analyses

HDR efficiencies were recorded in at least three replicates and were statistically analysed and plotted using PRISM 7.01 software. In Figure 1c, multiple comparisons of the HDR efficiencies of the other constructs with those of pRep^‐^ were performed by one‐way ANOVA (uncorrected Fisher’s LSD test, *n* = 3, *df* = 2, *t* = 4.4; 4.4 and 1.5 for pTC217; pHR01 and pgRNA^‐^, respectively). In Figure 1e, pairwise comparisons of the HDR efficiencies of pTC217 and pHR01 under the three lighting conditions were performed with Student’s *t* test (DD: *t* = 1.222, *df* = 4; 8 L/16 D: *t* = 2.424, *df* = 7, and 16 L/8 D: *t* = 3.059, *df* = 4). In Figure 1f, comparisons of the HDR efficiencies of pTC217 and pHR01 in the various temperature conditions were performed with Student’s *t* test (19 °C: *t* = 2.656, *df* = 2; 25 °C: *t* = 3.346, *df* = 2; 28 °C: *t* = 2.099, *df* = 5; and 31 °C: *t* = 4.551, *df* = 2). In Figure 2b, comparisons of the HDR efficiencies of the other multi‐replicon tools with pHR01 were performed with Student’s *t* test (MR01: *t* = 3.648, *df* = 3; MR02: *t* = 6.041, *df* = 3; MR03: *t* = 2.032, *df* = 3; and MR04: *t* = 1.893, *df* = 3).

## Conflict of interest

The authors have secured Korean patents (10‐2074744, 10‐2002443) and submitted PCT patent applications (application no. PCT/KR2019/011677, PCT/KR2019/000501) based on the results reported in this paper.

## Author contributions

T.V.V., V.S. and J.Y.K. designed the experiments. T.V.V., V.S., E.J.K., M.T.T., J.K., Y.W.S., D.T.H.D and M.P. performed the experiments. T.V.V., Y.J.K. and J.Y.K. analysed the results. T.V.V. and J.Y.K. wrote the manuscript.

## Supporting information


**Figure S1** Reengineering of the BeYDV Rep coding sequence used in the study.
**Figure S2** Schematic diagram of HDR‐directed editing of ANT1 locus.
**Figure S3** The *de novo‐*engineered geminiviral amplicon (named pLSL.R. Ly) and its replication in tomato.
**Figure S4** Schematic representation of the system and the released forms of the MR01 multi‐replicon system.
**Figure S5** Relative levels of SlRAD51 or SlRAD54 transcripts expressed in transgenic events carrying MR03 or MR04, respectively.
**Figure S6** Morphological appearance of GE0 plants.
**Figure S7** Sanger sequencing data to confirm donor exchanges.
**Figure S8** Error‐prone repair combining HDR and NHEJ in event #C1.3.
**Figure S9** PCR analyses of GE1 plants obtained from GE0 LbCpf1‐based HR events.
**Figure S10** Morphological appearance of GE1 plants.
**Figure S11** Analyses of left and right junction sequences of GE1 plants.
**Figure S12** Southern Blot analysis of the ANT1 edited locus.
**Figure S13** Analyses of indel mutations in HKT12 events.
**Figure S14** Morphology of the heterozygous HKT12^N217D^ event in a mature stage.
**Figure S15** Timeline and contents of the *Agro*‐mediated transformation protocol used in this work.

**Table S1A** Purple spot data collected in the experiment for comparison of HDR efficiency between different constructs.
**Table S1B** Purple spot data collected in the experiment for assessment of Impact of photoperiod on HDR.
**Table S1C** Purple spot data collected in the experiment for assessment of Impact of photoperiod on HDR.
**Table S2A** SlANT1 locus mutation rates observed from transformed events of pTC217.
**Table S2B** Indel mutation rates observed from transformed pHR01 events at SlANT1 sites.
**Table S3** The increase in HDR by multi‐replicon systems.
**Table S4** Primers for detecting circularized replicons released by MR01 and pHR01.
**Table S5** ANT1 HDR events derived from three main HDR constructs used in the study.
**Table S6** Primers for LbCpf1‐based HR event analyses.
**Table S7** Primers for SpCas9‐based HR event analyses.
**Table S8** Phenotypic segregation of self‐pollinated offspring resulting from LbCpf1‐based HDR events.
**Table S9** Summary of the *SlHKT1;2* HDR experiment.
**Table S10** Indel mutation rates among *HKT12* samples decomposed by ICE Synthego software.

**Data S1** Sequences used in the study.

**Data S2** Analysis of guide RNA activity.

**Data S3** Potential off‐targets of LbCpf1_gRNA1.

**Data S4** qRT‐PCR analyses of SlRAD51 and SlRAD54 mRNA levels.

**Data S5** Southern blot analysis of GE1 plants.
