# Supplementary material for: Highly efficient homology‐directed repair using CRISPR/Cpf1‐geminiviral replicon in tomato
Source: Plant Biotechnol J. 2020 Apr 1;18(10):2133–43. doi: 10.1111/pbi.13373 (PMC7540044; doi:10.1111/pbi.13373)
Supplement: Supplementary file 1 — Figure S1 Reengineering of the BeYDV Rep coding sequence used in the study. Figure S2 Schematic diagram of HDR‐directed editing of ANT1 locus. Figure S3 The de novo‐engineered geminiviral amplicon (named pLSL.R. Ly) and its replication in tomato. Figure S4 Schematic representation of the system and the released forms of the MR01 multi‐replicon system. Figure S5 Relative levels of SlRAD51 or SlRAD54 transcripts expressed in transgenic events carrying MR03 or MR04, respectively. Figure S6 Morphological appearance of GE0 plants. Figure S7 Sanger sequencing data to confirm donor exchanges. Figure S8 Error‐prone repair combining HDR and NHEJ in event #C1.3. Figure S9 PCR analyses of GE1 plants obtained from GE0 LbCpf1‐based HR events. Figure S10 Morphological appearance of GE1 plants. Figure S11 Analyses of left and right junction sequences of GE1 plants. Figure S12 Southern Blot analysis of the ANT1 edited locus. Figure S13 Analyses of indel mutations in HKT12 events. Figure S14 Morphology of the heterozygous HKT12N217D event in a mature stage. Figure S15 Timeline and contents of the Agro‐mediated transformation protocol used in this work. [file PBI-18-2133-s005.pptx]

## Slide 1
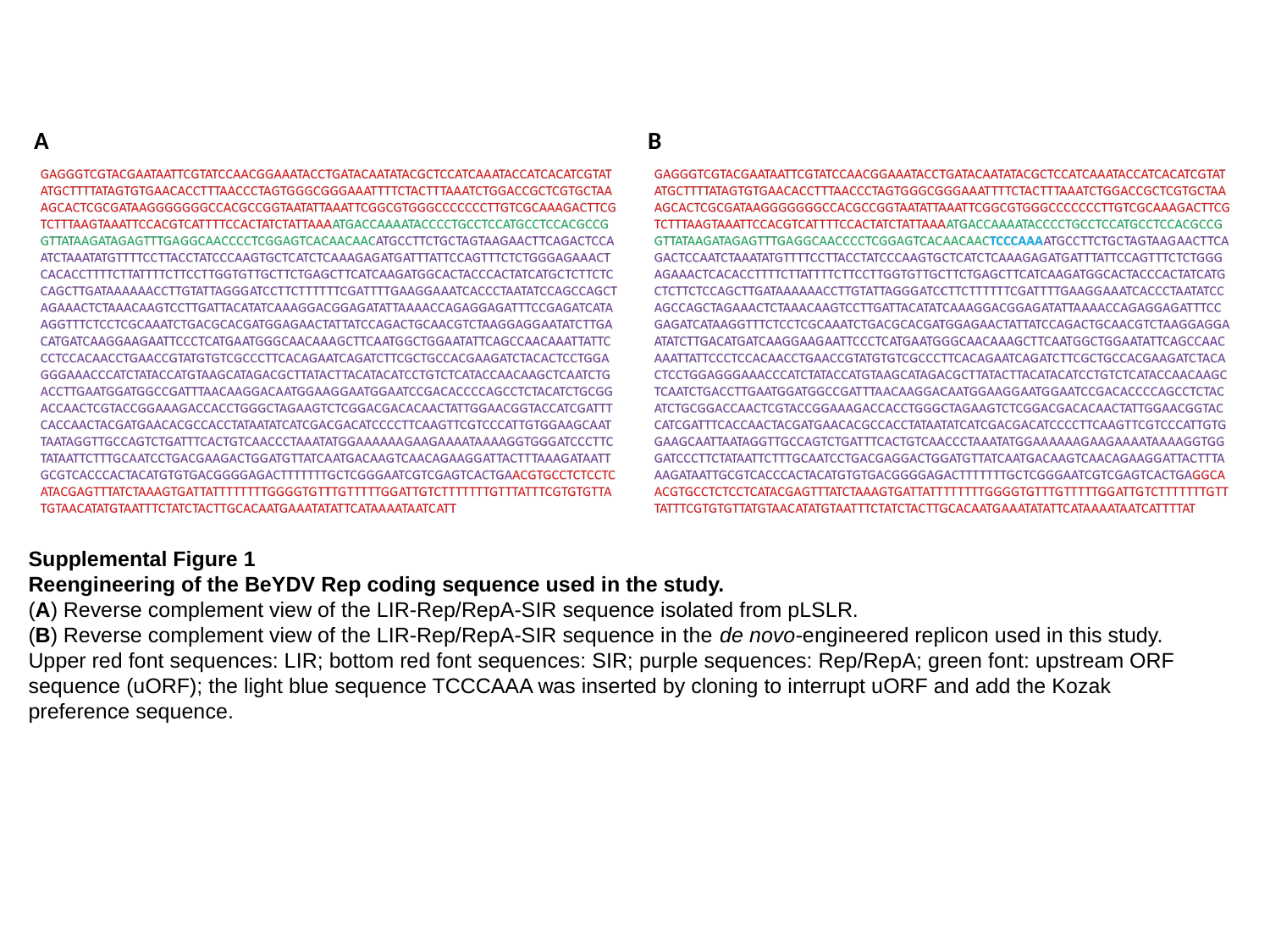

A
B
Supplemental Figure 1
Reengineering of the BeYDV Rep coding sequence used in the study.
(A) Reverse complement view of the LIR-Rep/RepA-SIR sequence isolated from pLSLR.
(B) Reverse complement view of the LIR-Rep/RepA-SIR sequence in the de novo-engineered replicon used in this study.
Upper red font sequences: LIR; bottom red font sequences: SIR; purple sequences: Rep/RepA; green font: upstream ORF sequence (uORF); the light blue sequence TCCCAAA was inserted by cloning to interrupt uORF and add the Kozak preference sequence.

## Slide 2
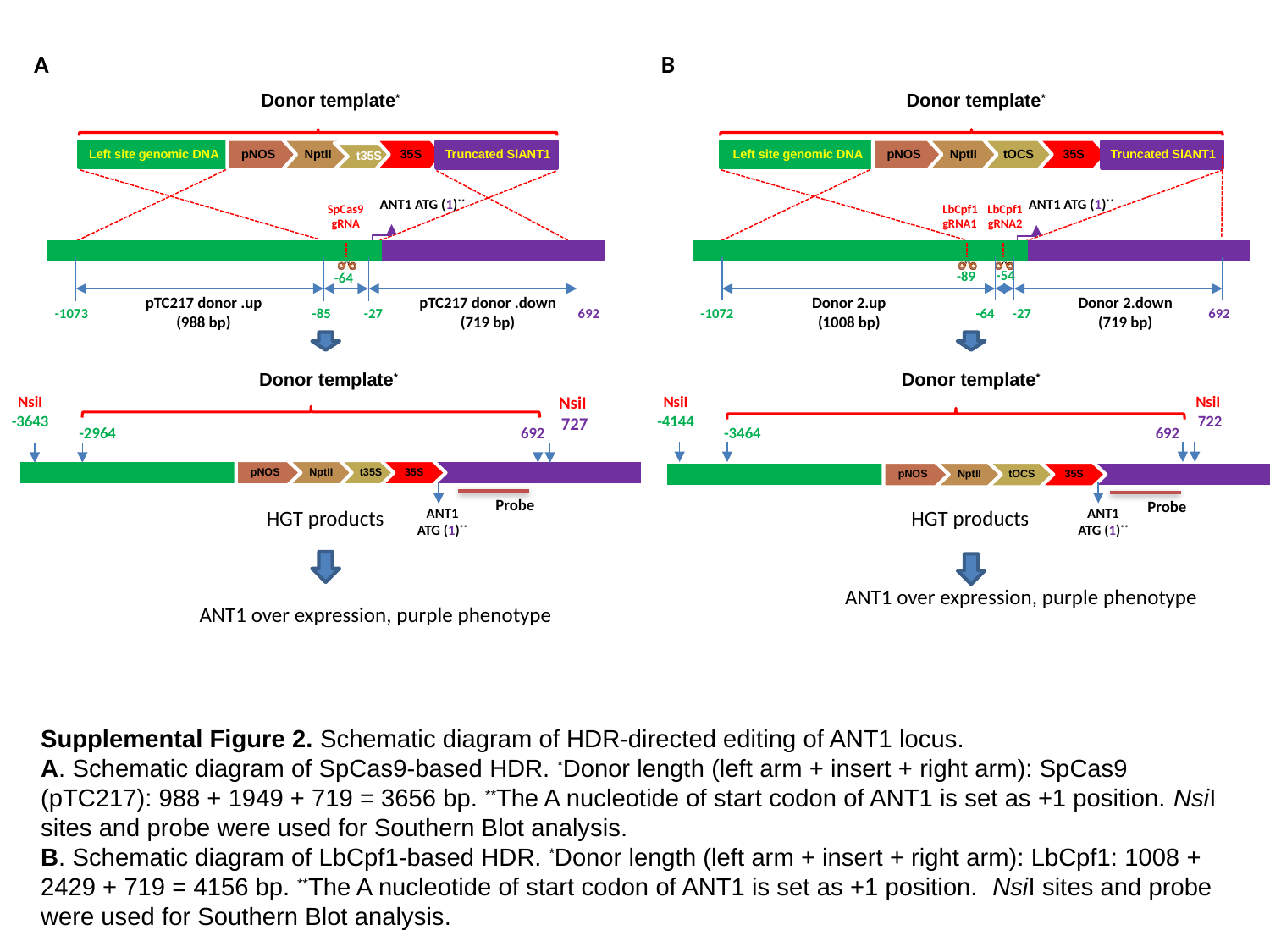

A
B
Donor template*
Donor template*
pNOS
NptII
35S
Left site genomic DNA
Truncated SlANT1
t35S
pNOS
NptII
tOCS
35S
Left site genomic DNA
Truncated SlANT1
ANT1 ATG (1)**
ANT1 ATG (1)**
SpCas9
gRNA
LbCpf1
gRNA1
LbCpf1
gRNA2
-54
-89
-64
pTC217 donor .up (988 bp)
pTC217 donor .down (719 bp)
Donor 2.up (1008 bp)
Donor 2.down (719 bp)
-1072
-64
-27
692
-1073
-85
-27
692
Donor template*
Donor template*
NsiI
-3643
NsiI
727
NsiI
-4144
NsiI
722
-2964
692
-3464
692
pNOS
NptII
t35S
35S
pNOS
NptII
tOCS
35S
Probe
Probe
ANT1 ATG (1)**
ANT1 ATG (1)**
HGT products
HGT products
ANT1 over expression, purple phenotype
ANT1 over expression, purple phenotype
Supplemental Figure 2. Schematic diagram of HDR-directed editing of ANT1 locus.
A. Schematic diagram of SpCas9-based HDR. *Donor length (left arm + insert + right arm): SpCas9 (pTC217): 988 + 1949 + 719 = 3656 bp. **The A nucleotide of start codon of ANT1 is set as +1 position. NsiI sites and probe were used for Southern Blot analysis.
B. Schematic diagram of LbCpf1-based HDR. *Donor length (left arm + insert + right arm): LbCpf1: 1008 + 2429 + 719 = 4156 bp. **The A nucleotide of start codon of ANT1 is set as +1 position. NsiI sites and probe were used for Southern Blot analysis.

## Slide 3
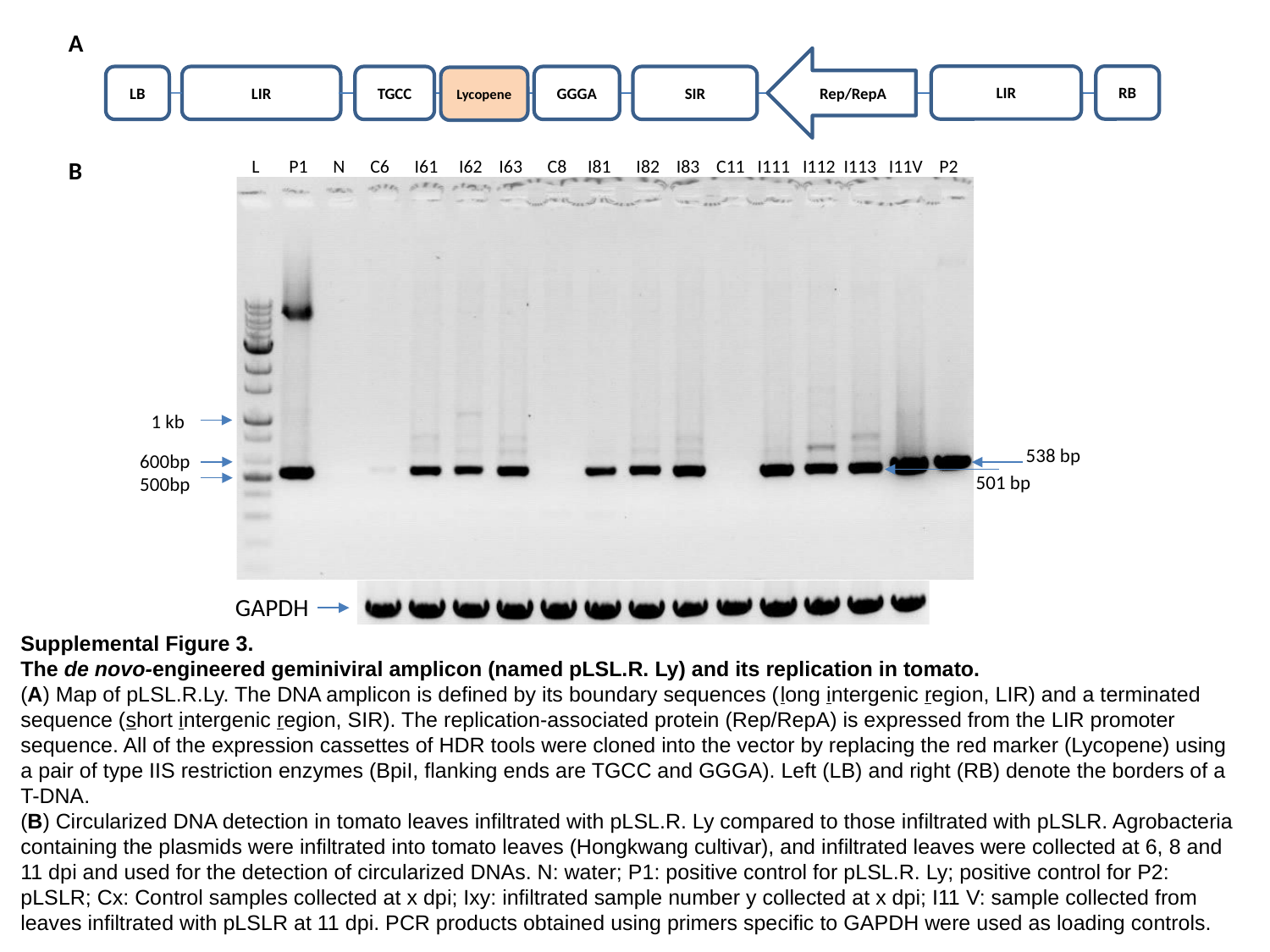

A
Rep/RepA
LIR
RB
LB
LIR
TGCC
GGGA
SIR
Lycopene
B
 L P1 N C6 I61 I62 I63 C8 I81 I82 I83 C11 I111 I112 I113 I11V P2
1 kb
538 bp
600bp
501 bp
500bp
GAPDH
Supplemental Figure 3.
The de novo-engineered geminiviral amplicon (named pLSL.R. Ly) and its replication in tomato.
(A) Map of pLSL.R.Ly. The DNA amplicon is defined by its boundary sequences (long intergenic region, LIR) and a terminated sequence (short intergenic region, SIR). The replication-associated protein (Rep/RepA) is expressed from the LIR promoter sequence. All of the expression cassettes of HDR tools were cloned into the vector by replacing the red marker (Lycopene) using a pair of type IIS restriction enzymes (BpiI, flanking ends are TGCC and GGGA). Left (LB) and right (RB) denote the borders of a T-DNA.
(B) Circularized DNA detection in tomato leaves infiltrated with pLSL.R. Ly compared to those infiltrated with pLSLR. Agrobacteria containing the plasmids were infiltrated into tomato leaves (Hongkwang cultivar), and infiltrated leaves were collected at 6, 8 and 11 dpi and used for the detection of circularized DNAs. N: water; P1: positive control for pLSL.R. Ly; positive control for P2: pLSLR; Cx: Control samples collected at x dpi; Ixy: infiltrated sample number y collected at x dpi; I11 V: sample collected from leaves infiltrated with pLSLR at 11 dpi. PCR products obtained using primers specific to GAPDH were used as loading controls.

## Slide 4
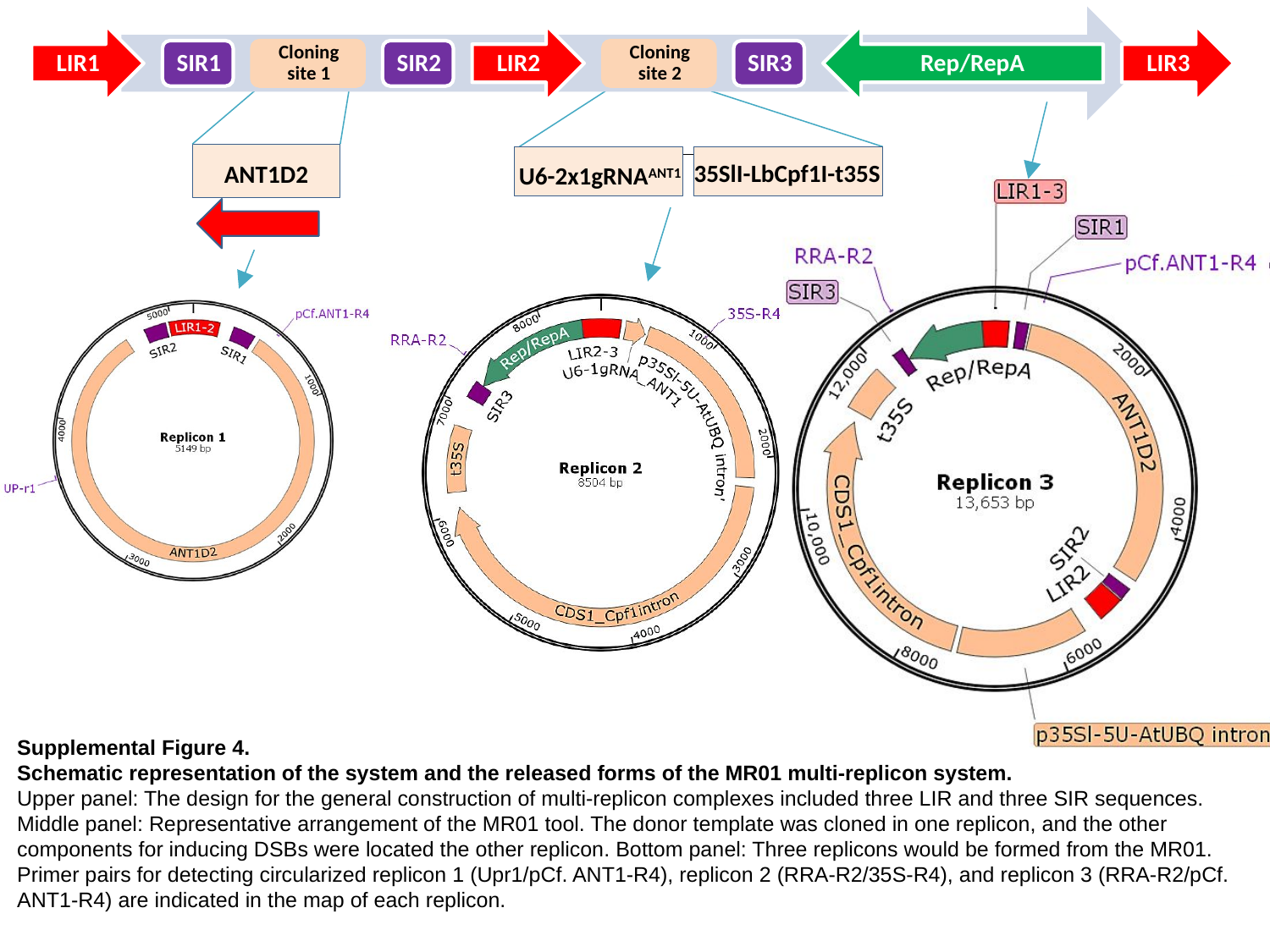

U6-2x1gRNAANT1
35SlI-LbCpf1I-t35S
ANT1D2
Supplemental Figure 4.
Schematic representation of the system and the released forms of the MR01 multi-replicon system.
Upper panel: The design for the general construction of multi-replicon complexes included three LIR and three SIR sequences. Middle panel: Representative arrangement of the MR01 tool. The donor template was cloned in one replicon, and the other components for inducing DSBs were located the other replicon. Bottom panel: Three replicons would be formed from the MR01. Primer pairs for detecting circularized replicon 1 (Upr1/pCf. ANT1-R4), replicon 2 (RRA-R2/35S-R4), and replicon 3 (RRA-R2/pCf. ANT1-R4) are indicated in the map of each replicon.

## Slide 5
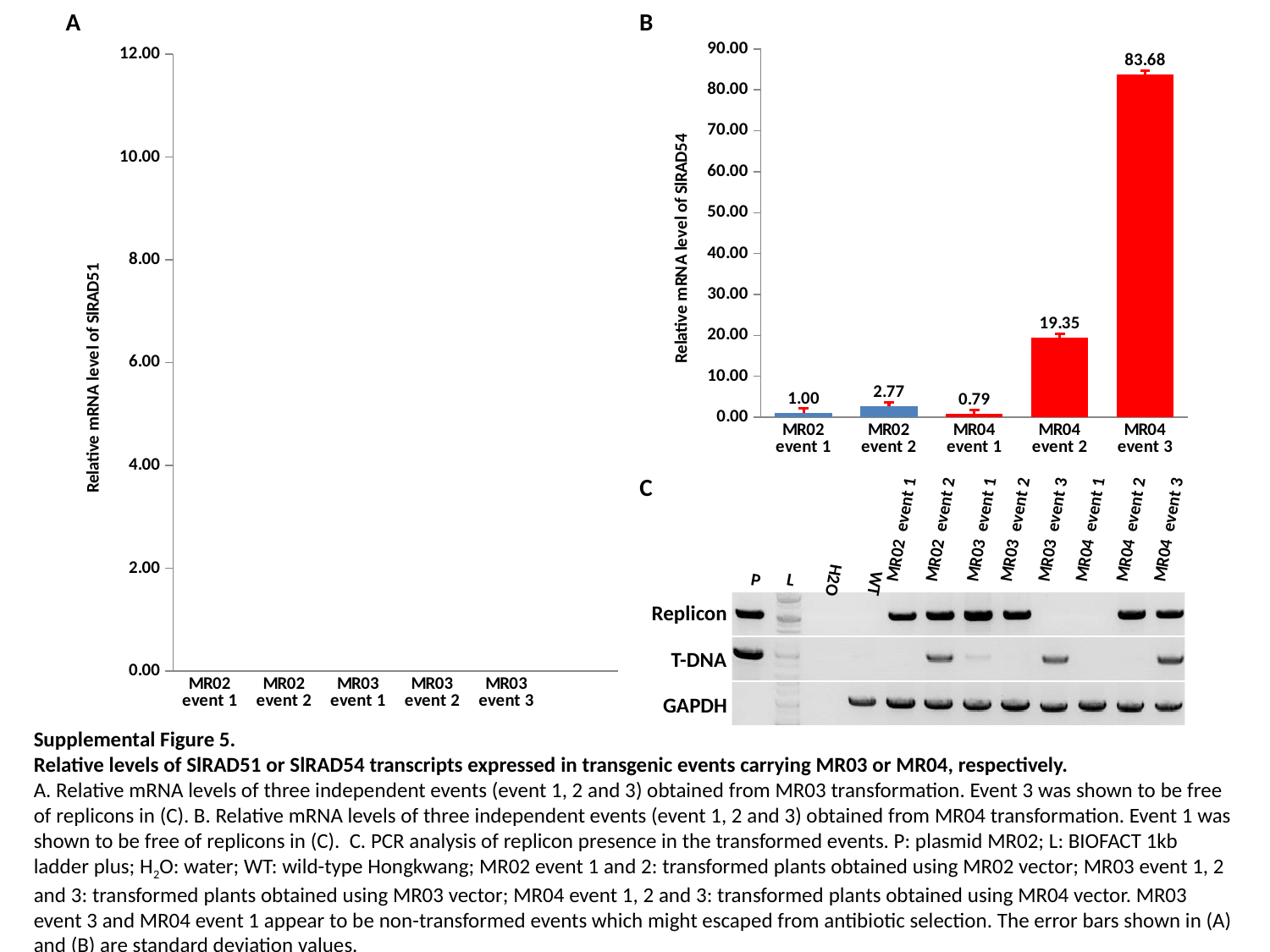

A
B
### Chart
| Category | Fold change |
|---|---|
| MR02 event 1 | 0.9999999999999993 |
| MR02 event 2 | 2.0571908775862324 |
| MR03 event 1 | 522.1757365449257 |
| MR03 event 2 | 46.66254123325564 |
| MR03 event 3 | 1.2619630645305913 |
### Chart
| Category | Fold change |
|---|---|
| MR02 event 1 | 1.0 |
| MR02 event 2 | 2.7652217025390864 |
| MR04 event 1 | 0.792865006005316 |
| MR04 event 2 | 19.353094692826446 |
| MR04 event 3 | 83.6778970454749 |MR02 event 1
MR02 event 2
MR03 event 1
MR03 event 2
MR03 event 3
MR04 event 1
MR04 event 2
MR04 event 3
H2O
WT
P
L
Replicon
T-DNA
GAPDH
C
Supplemental Figure 5.
Relative levels of SlRAD51 or SlRAD54 transcripts expressed in transgenic events carrying MR03 or MR04, respectively.
A. Relative mRNA levels of three independent events (event 1, 2 and 3) obtained from MR03 transformation. Event 3 was shown to be free of replicons in (C). B. Relative mRNA levels of three independent events (event 1, 2 and 3) obtained from MR04 transformation. Event 1 was shown to be free of replicons in (C). C. PCR analysis of replicon presence in the transformed events. P: plasmid MR02; L: BIOFACT 1kb ladder plus; H2O: water; WT: wild-type Hongkwang; MR02 event 1 and 2: transformed plants obtained using MR02 vector; MR03 event 1, 2 and 3: transformed plants obtained using MR03 vector; MR04 event 1, 2 and 3: transformed plants obtained using MR04 vector. MR03 event 3 and MR04 event 1 appear to be non-transformed events which might escaped from antibiotic selection. The error bars shown in (A) and (B) are standard deviation values.

## Slide 6
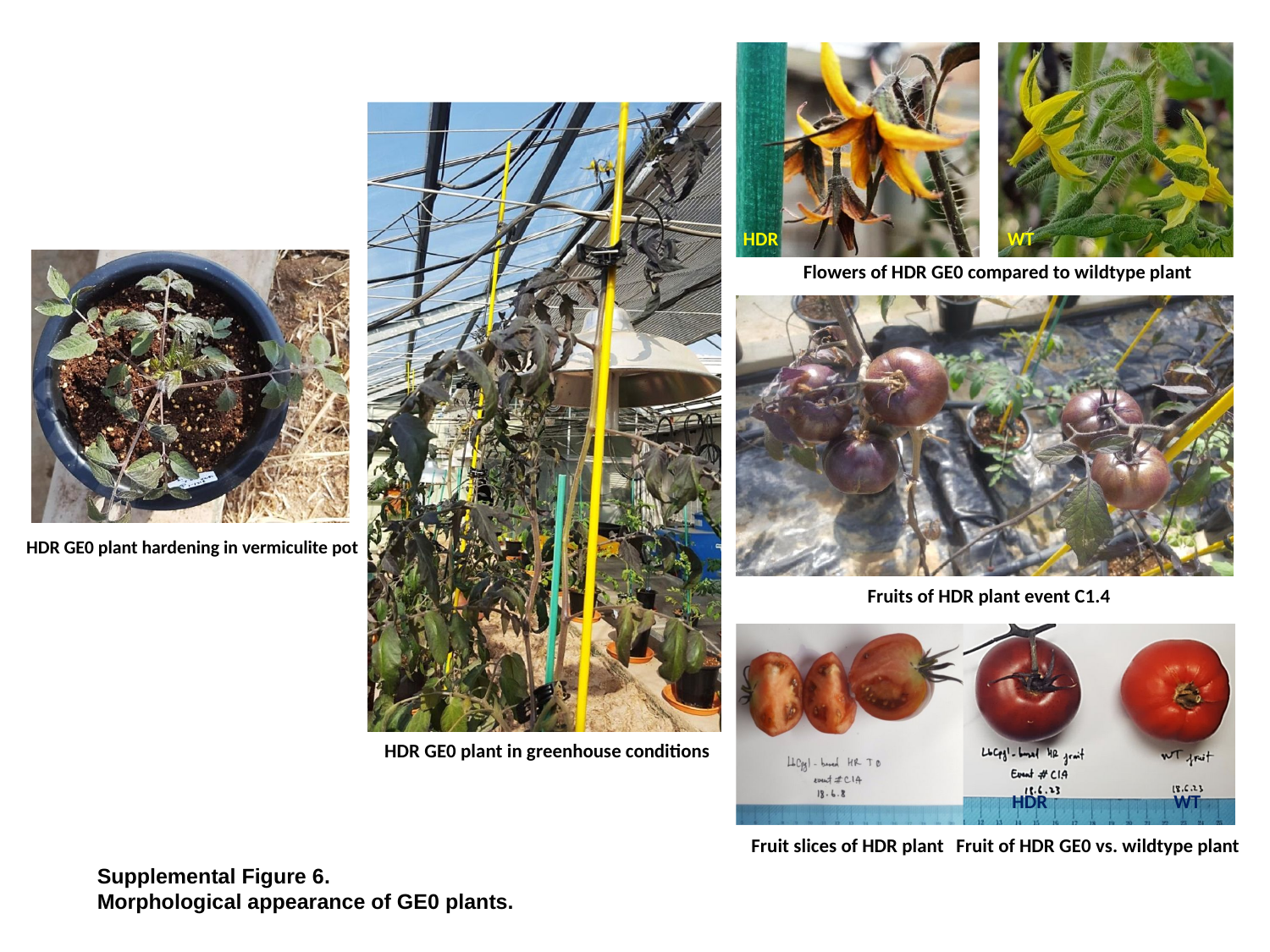

HDR
WT
Flowers of HDR GE0 compared to wildtype plant
Fruits of HDR plant event C1.4
WT
HDR
Fruit slices of HDR plant
Fruit of HDR GE0 vs. wildtype plant
HDR GE0 plant hardening in vermiculite pot
HDR GE0 plant in greenhouse conditions
Supplemental Figure 6.
Morphological appearance of GE0 plants.

## Slide 7
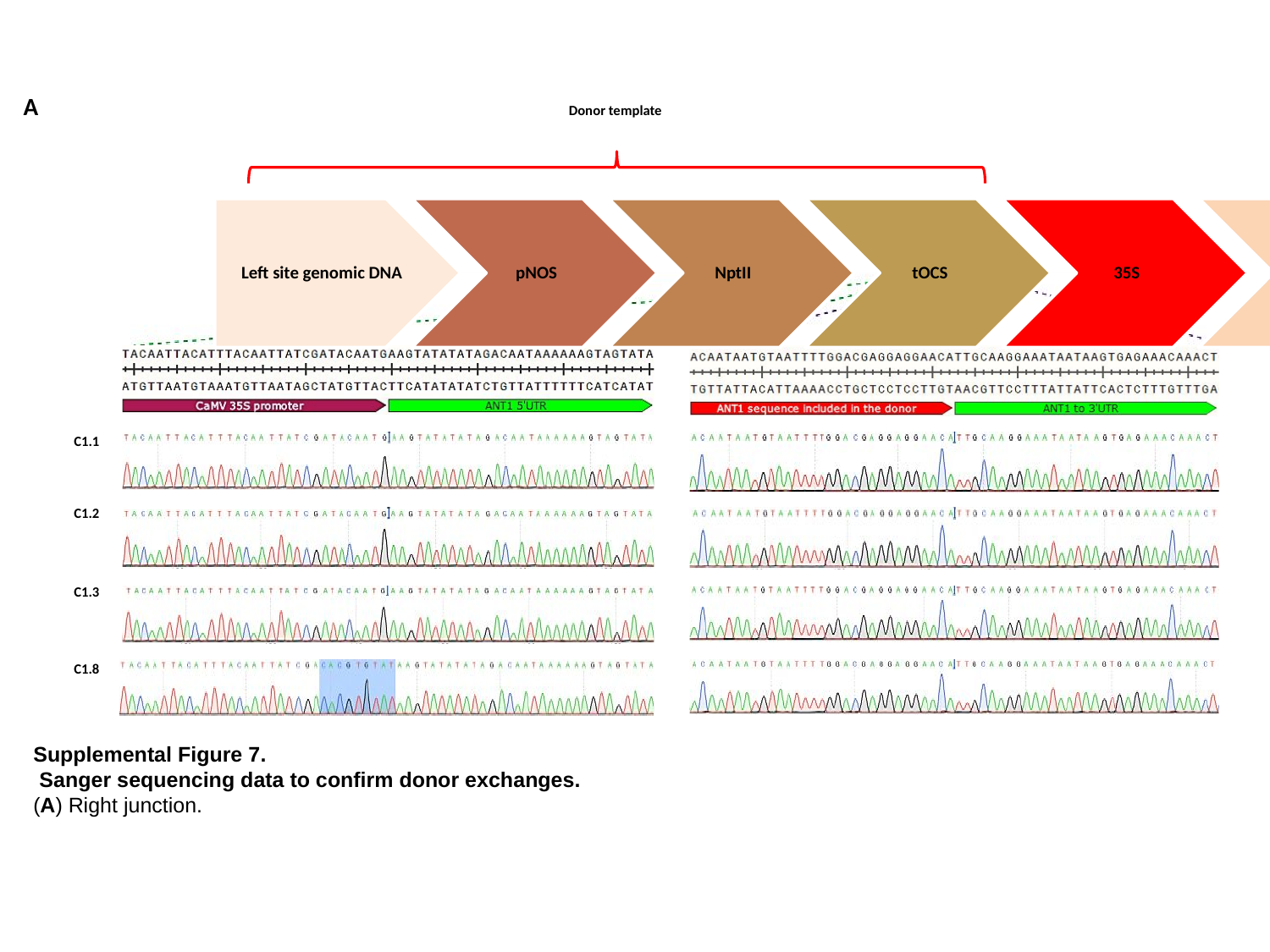

A
Donor template
C1.1
C1.2
C1.3
C1.8
Supplemental Figure 7.
 Sanger sequencing data to confirm donor exchanges.
(A) Right junction.

## Slide 8
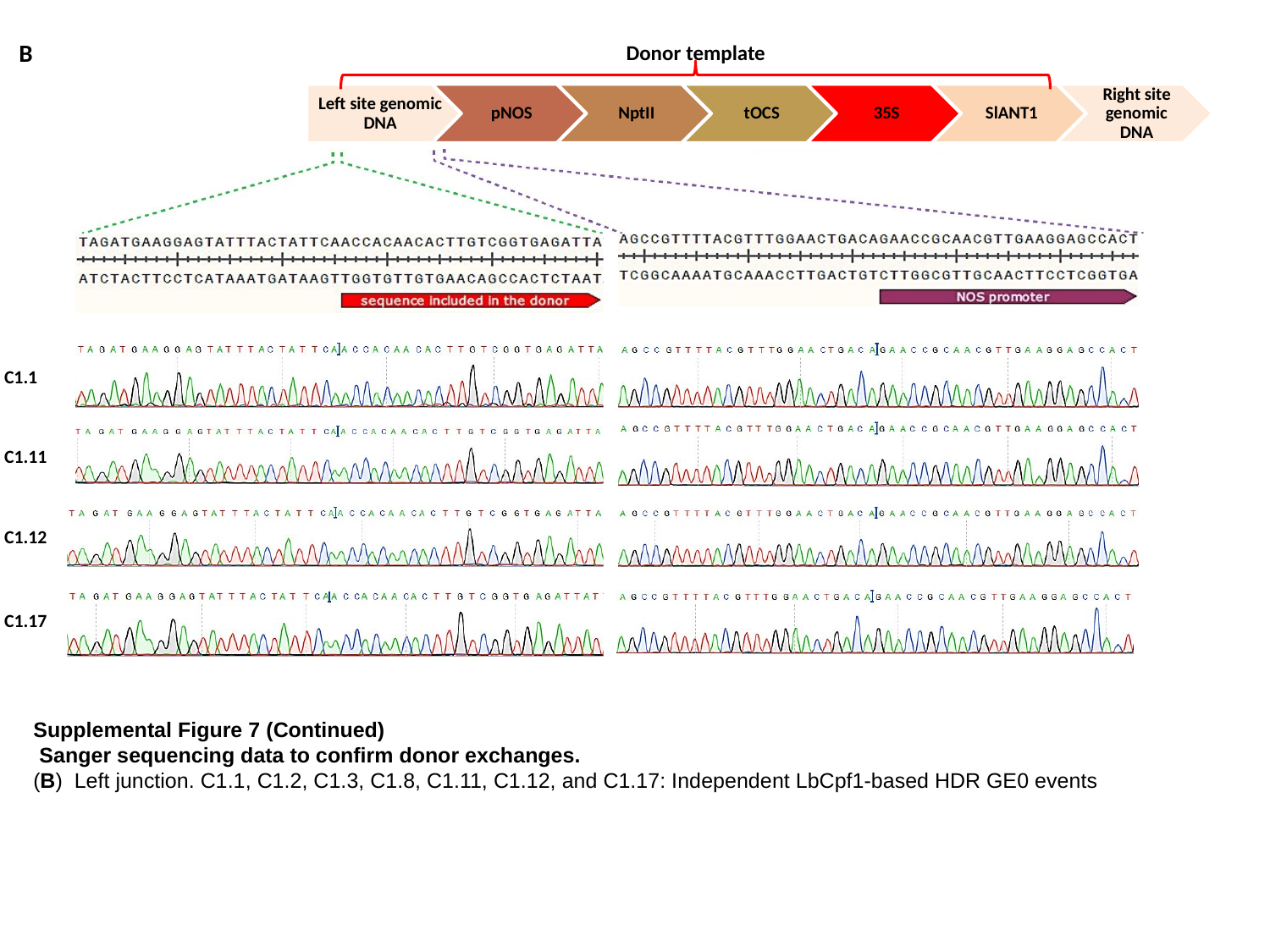

B
Donor template
C1.1
C1.11
C1.12
C1.17
Supplemental Figure 7 (Continued)
 Sanger sequencing data to confirm donor exchanges.
(B) Left junction. C1.1, C1.2, C1.3, C1.8, C1.11, C1.12, and C1.17: Independent LbCpf1-based HDR GE0 events

## Slide 9
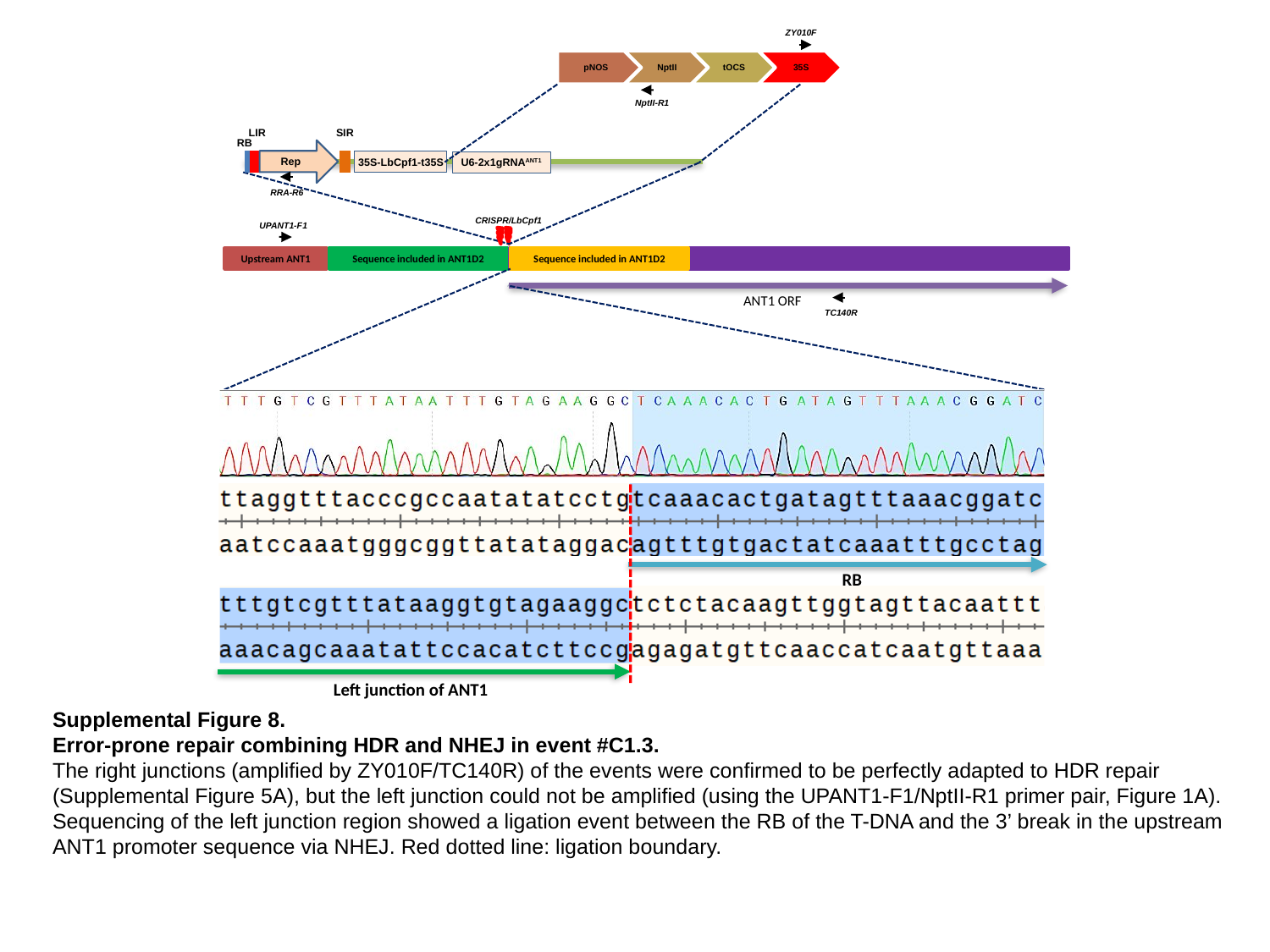

ZY010F
pNOS
NptII
tOCS
35S
NptII-R1
LIR
SIR
RB
Rep
35S-LbCpf1-t35S
U6-2x1gRNAANT1
RRA-R6
CRISPR/LbCpf1
UPANT1-F1
Upstream ANT1
Sequence included in ANT1D2
Sequence included in ANT1D2
ANT1 ORF
TC140R
RB
Left junction of ANT1
Supplemental Figure 8.
Error-prone repair combining HDR and NHEJ in event #C1.3.
The right junctions (amplified by ZY010F/TC140R) of the events were confirmed to be perfectly adapted to HDR repair (Supplemental Figure 5A), but the left junction could not be amplified (using the UPANT1-F1/NptII-R1 primer pair, Figure 1A). Sequencing of the left junction region showed a ligation event between the RB of the T-DNA and the 3’ break in the upstream ANT1 promoter sequence via NHEJ. Red dotted line: ligation boundary.

## Slide 10
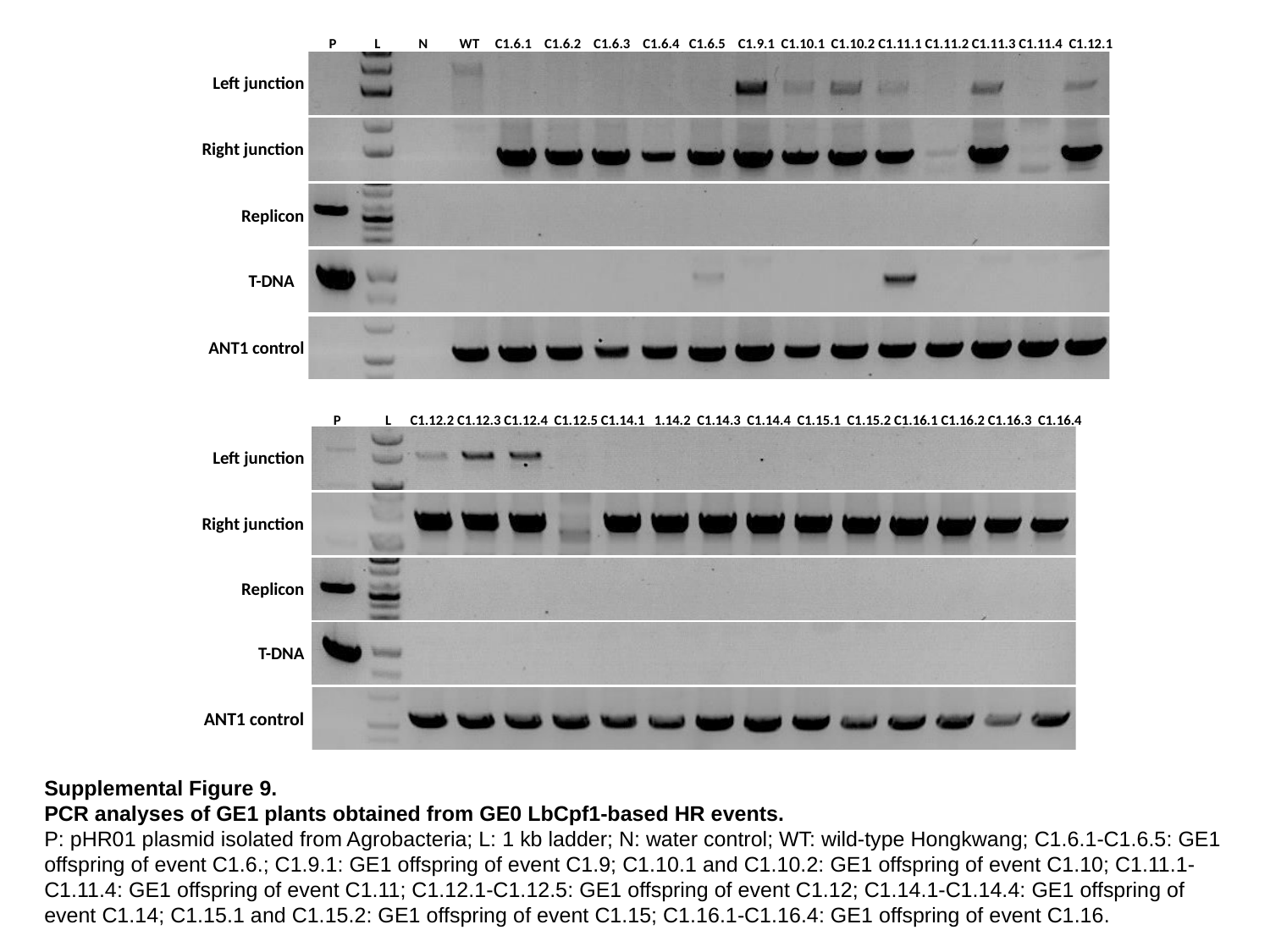

P L N WT C1.6.1 C1.6.2 C1.6.3 C1.6.4 C1.6.5 C1.9.1 C1.10.1 C1.10.2 C1.11.1 C1.11.2 C1.11.3 C1.11.4 C1.12.1
Left junction
Right junction
Replicon
T-DNA
ANT1 control
 P L C1.12.2 C1.12.3 C1.12.4 C1.12.5 C1.14.1 1.14.2 C1.14.3 C1.14.4 C1.15.1 C1.15.2 C1.16.1 C1.16.2 C1.16.3 C1.16.4
Left junction
Right junction
Replicon
T-DNA
ANT1 control
Supplemental Figure 9.
PCR analyses of GE1 plants obtained from GE0 LbCpf1-based HR events.
P: pHR01 plasmid isolated from Agrobacteria; L: 1 kb ladder; N: water control; WT: wild-type Hongkwang; C1.6.1-C1.6.5: GE1 offspring of event C1.6.; C1.9.1: GE1 offspring of event C1.9; C1.10.1 and C1.10.2: GE1 offspring of event C1.10; C1.11.1-C1.11.4: GE1 offspring of event C1.11; C1.12.1-C1.12.5: GE1 offspring of event C1.12; C1.14.1-C1.14.4: GE1 offspring of event C1.14; C1.15.1 and C1.15.2: GE1 offspring of event C1.15; C1.16.1-C1.16.4: GE1 offspring of event C1.16.

## Slide 11
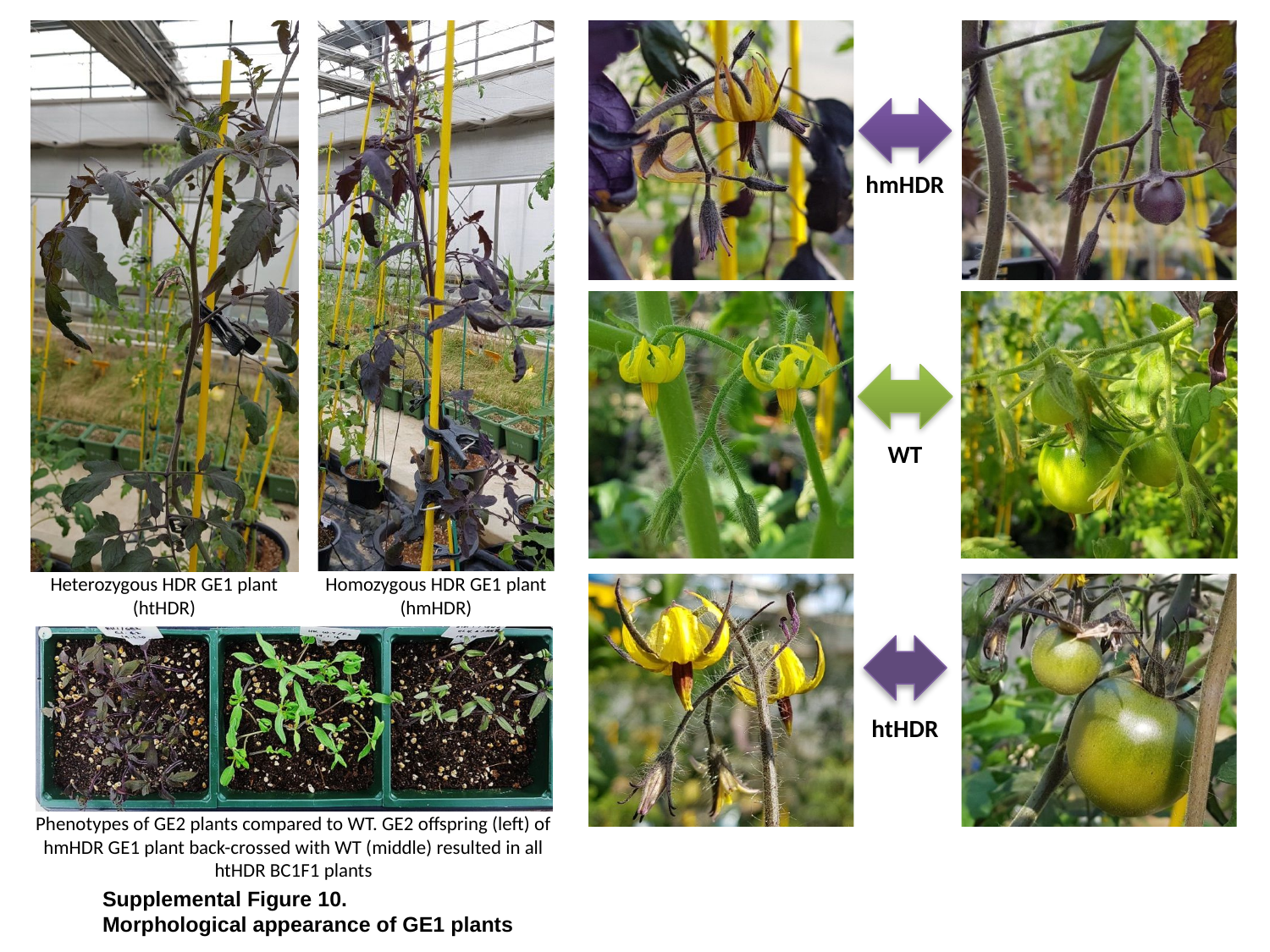

Heterozygous HDR GE1 plant (htHDR)
Homozygous HDR GE1 plant (hmHDR)
hmHDR
WT
htHDR
Phenotypes of GE2 plants compared to WT. GE2 offspring (left) of hmHDR GE1 plant back-crossed with WT (middle) resulted in all htHDR BC1F1 plants
Supplemental Figure 10.
Morphological appearance of GE1 plants

## Slide 12
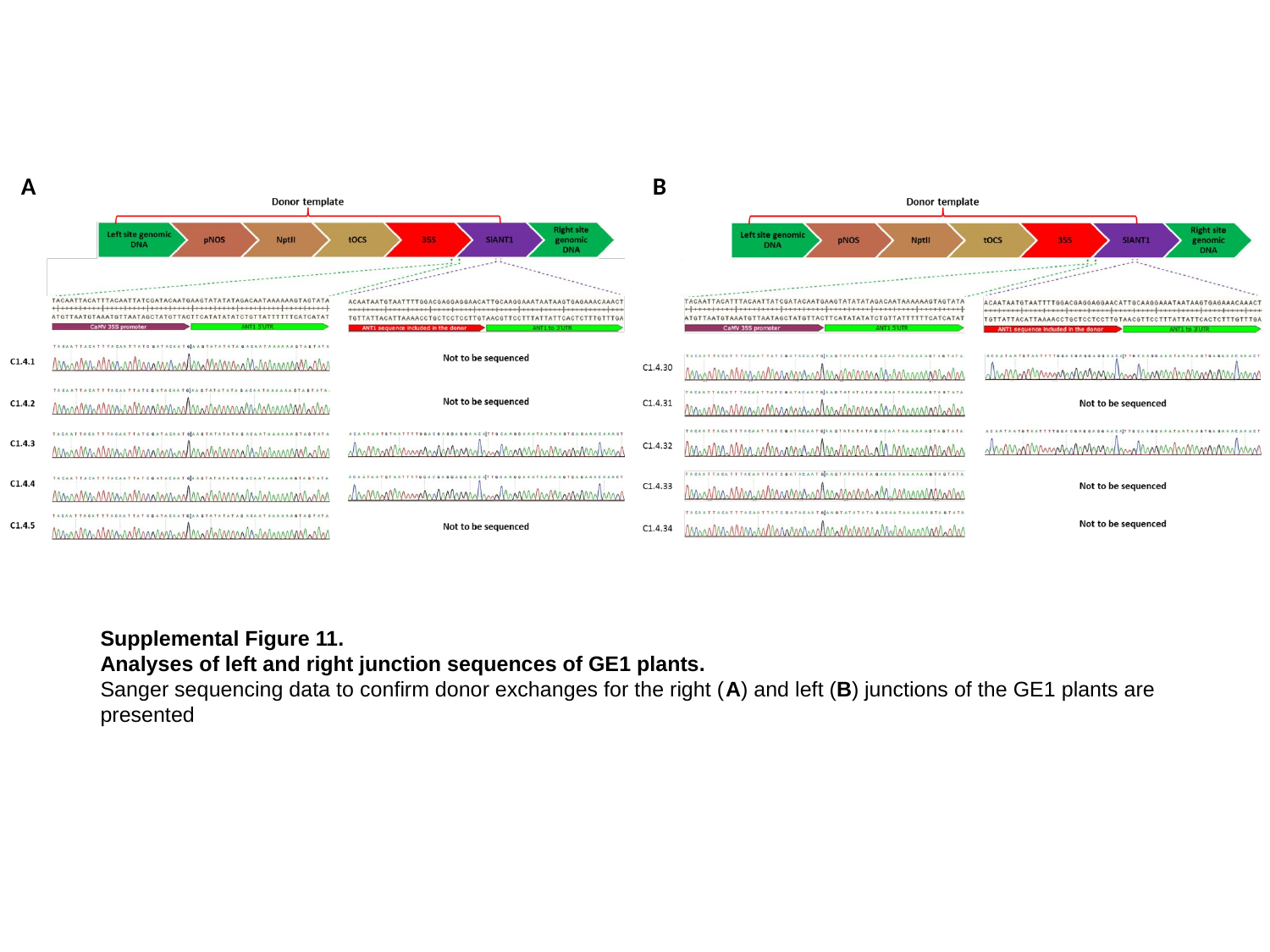

A
B
Supplemental Figure 11.
Analyses of left and right junction sequences of GE1 plants.
Sanger sequencing data to confirm donor exchanges for the right (A) and left (B) junctions of the GE1 plants are presented

## Slide 13
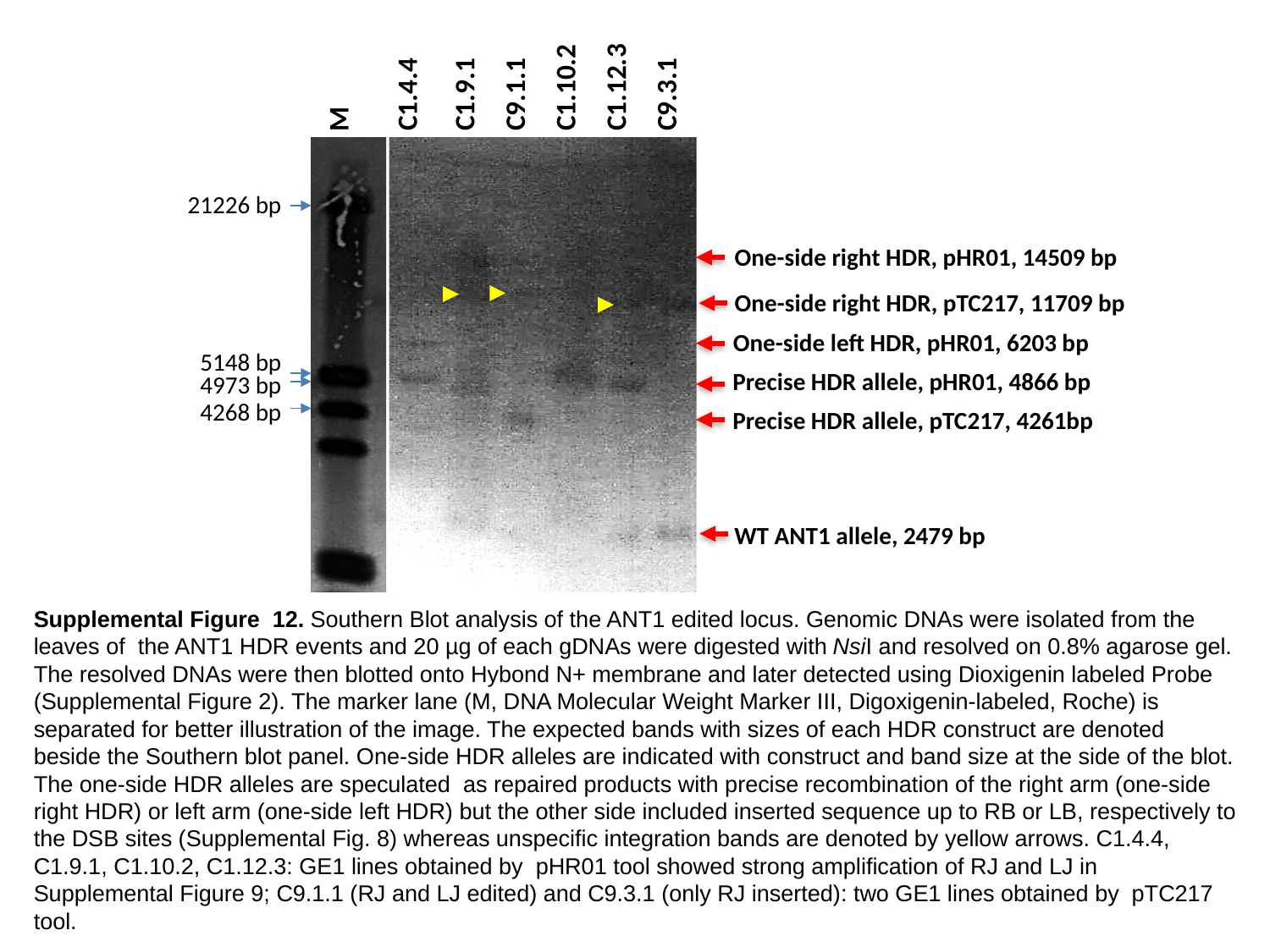

| C1.4.4 | C1.9.1 | C9.1.1 | C1.10.2 | C1.12.3 | C9.3.1 |
| --- | --- | --- | --- | --- | --- |
| M |
| --- |
21226 bp
 One-side right HDR, pHR01, 14509 bp
 One-side right HDR, pTC217, 11709 bp
One-side left HDR, pHR01, 6203 bp
5148 bp
Precise HDR allele, pHR01, 4866 bp
4973 bp
4268 bp
Precise HDR allele, pTC217, 4261bp
WT ANT1 allele, 2479 bp
Supplemental Figure 12. Southern Blot analysis of the ANT1 edited locus. Genomic DNAs were isolated from the leaves of the ANT1 HDR events and 20 µg of each gDNAs were digested with NsiI and resolved on 0.8% agarose gel. The resolved DNAs were then blotted onto Hybond N+ membrane and later detected using Dioxigenin labeled Probe (Supplemental Figure 2). The marker lane (M, DNA Molecular Weight Marker III, Digoxigenin-labeled, Roche) is separated for better illustration of the image. The expected bands with sizes of each HDR construct are denoted beside the Southern blot panel. One-side HDR alleles are indicated with construct and band size at the side of the blot. The one-side HDR alleles are speculated as repaired products with precise recombination of the right arm (one-side right HDR) or left arm (one-side left HDR) but the other side included inserted sequence up to RB or LB, respectively to the DSB sites (Supplemental Fig. 8) whereas unspecific integration bands are denoted by yellow arrows. C1.4.4, C1.9.1, C1.10.2, C1.12.3: GE1 lines obtained by pHR01 tool showed strong amplification of RJ and LJ in Supplemental Figure 9; C9.1.1 (RJ and LJ edited) and C9.3.1 (only RJ inserted): two GE1 lines obtained by pTC217 tool.

## Slide 14
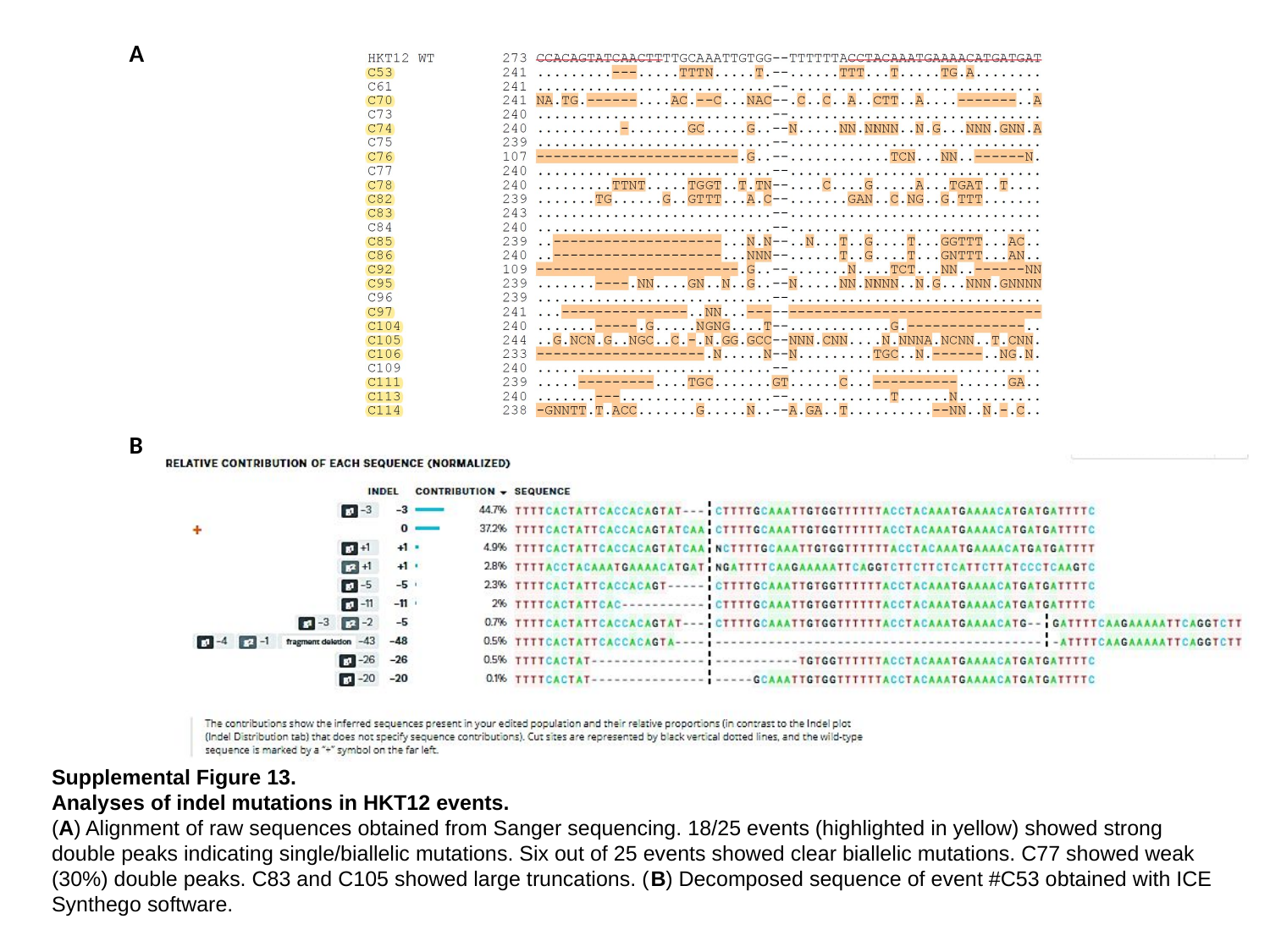

A
B
Supplemental Figure 13.
Analyses of indel mutations in HKT12 events.
(A) Alignment of raw sequences obtained from Sanger sequencing. 18/25 events (highlighted in yellow) showed strong double peaks indicating single/biallelic mutations. Six out of 25 events showed clear biallelic mutations. C77 showed weak (30%) double peaks. C83 and C105 showed large truncations. (B) Decomposed sequence of event #C53 obtained with ICE Synthego software.

## Slide 15
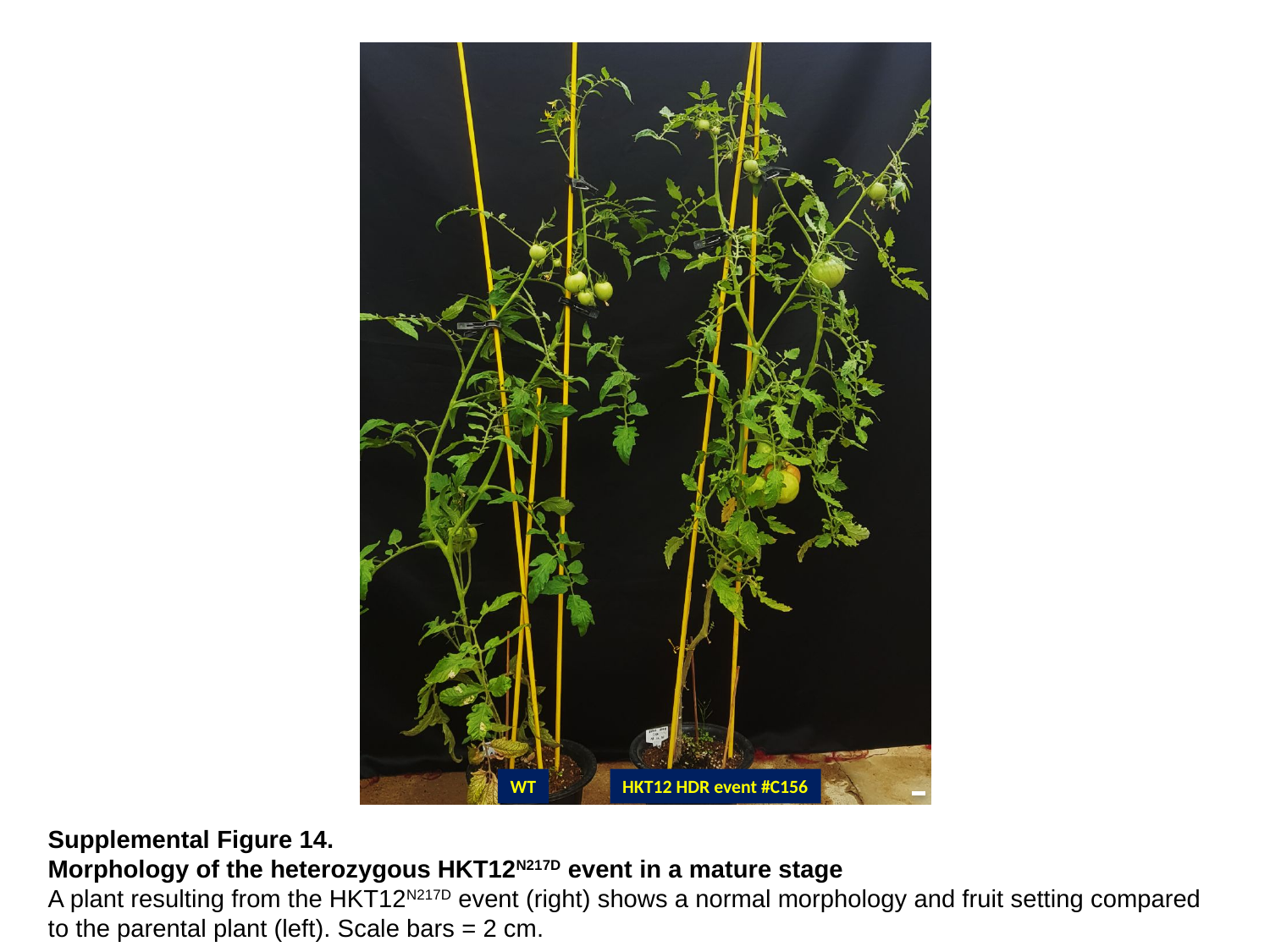

WT
HKT12 HDR event #C156
Supplemental Figure 14.
Morphology of the heterozygous HKT12N217D event in a mature stage
A plant resulting from the HKT12N217D event (right) shows a normal morphology and fruit setting compared to the parental plant (left). Scale bars = 2 cm.

## Slide 16
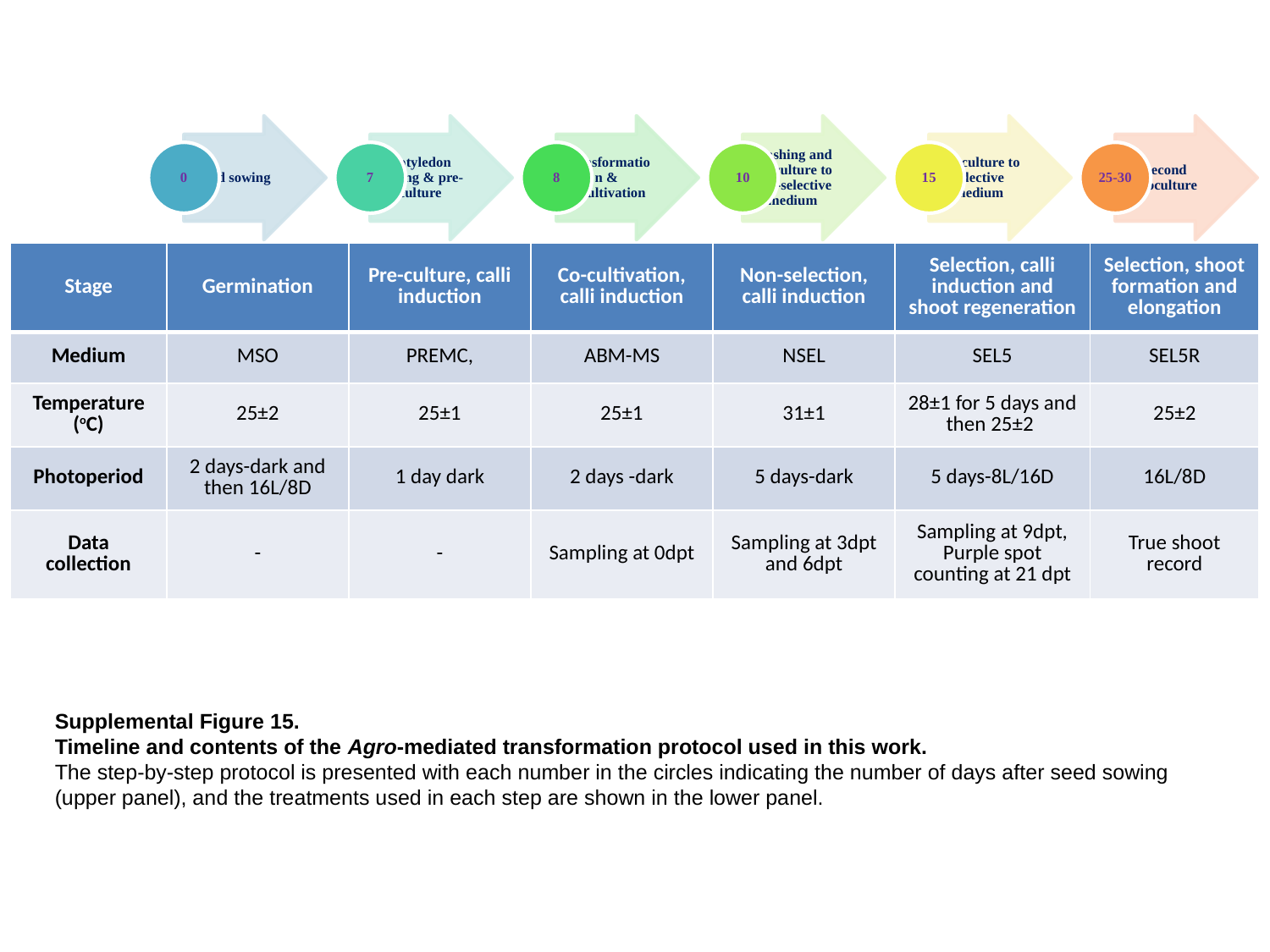

| Stage | Germination | Pre-culture, calli induction | Co-cultivation, calli induction | Non-selection, calli induction | Selection, calli induction and shoot regeneration | Selection, shoot formation and elongation |
| --- | --- | --- | --- | --- | --- | --- |
| Medium | MSO | PREMC, | ABM-MS | NSEL | SEL5 | SEL5R |
| Temperature (oC) | 25±2 | 25±1 | 25±1 | 31±1 | 28±1 for 5 days and then 25±2 | 25±2 |
| Photoperiod | 2 days-dark and then 16L/8D | 1 day dark | 2 days -dark | 5 days-dark | 5 days-8L/16D | 16L/8D |
| Data collection | - | - | Sampling at 0dpt | Sampling at 3dpt and 6dpt | Sampling at 9dpt, Purple spot counting at 21 dpt | True shoot record |
Supplemental Figure 15.
Timeline and contents of the Agro-mediated transformation protocol used in this work.
The step-by-step protocol is presented with each number in the circles indicating the number of days after seed sowing (upper panel), and the treatments used in each step are shown in the lower panel.
