# Supplementary material for: Highly efficient homology‐directed repair using CRISPR/Cpf1‐geminiviral replicon in tomato
Source: Plant Biotechnol J. 2020 Apr 1;18(10):2133–43. doi: 10.1111/pbi.13373 (PMC7540044; doi:10.1111/pbi.13373)
Supplement: Supplementary file 2 — Table S1A Purple spot data collected in the experiment for comparison of HDR efficiency between different constructs. Table S1B Purple spot data collected in the experiment for assessment of Impact of photoperiod on HDR. Table S1C Purple spot data collected in the experiment for assessment of Impact of photoperiod on HDR. Table S2A SlANT1 locus mutation rates observed from transformed events of pTC217. Table S2B Indel mutation rates observed from transformed pHR01 events at SlANT1 sites. Table S3 The increase in HDR by multi‐replicon systems. Table S4 Primers for detecting circularized replicons released by MR01 and pHR01. Table S5 ANT1 HDR events derived from three main HDR constructs used in the study. Table S6 Primers for LbCpf1‐based HR event analyses. Table S7 Primers for SpCas9‐based HR event analyses. Table S8 Phenotypic segregation of self‐pollinated offspring resulting from LbCpf1‐based HDR events. Table S9 Summary of the SlHKT1;2 HDR experiment. Table S10 Indel mutation rates among HKT12 samples decomposed by ICE Synthego software. [file PBI-18-2133-s004.pptx]

## Slide 1
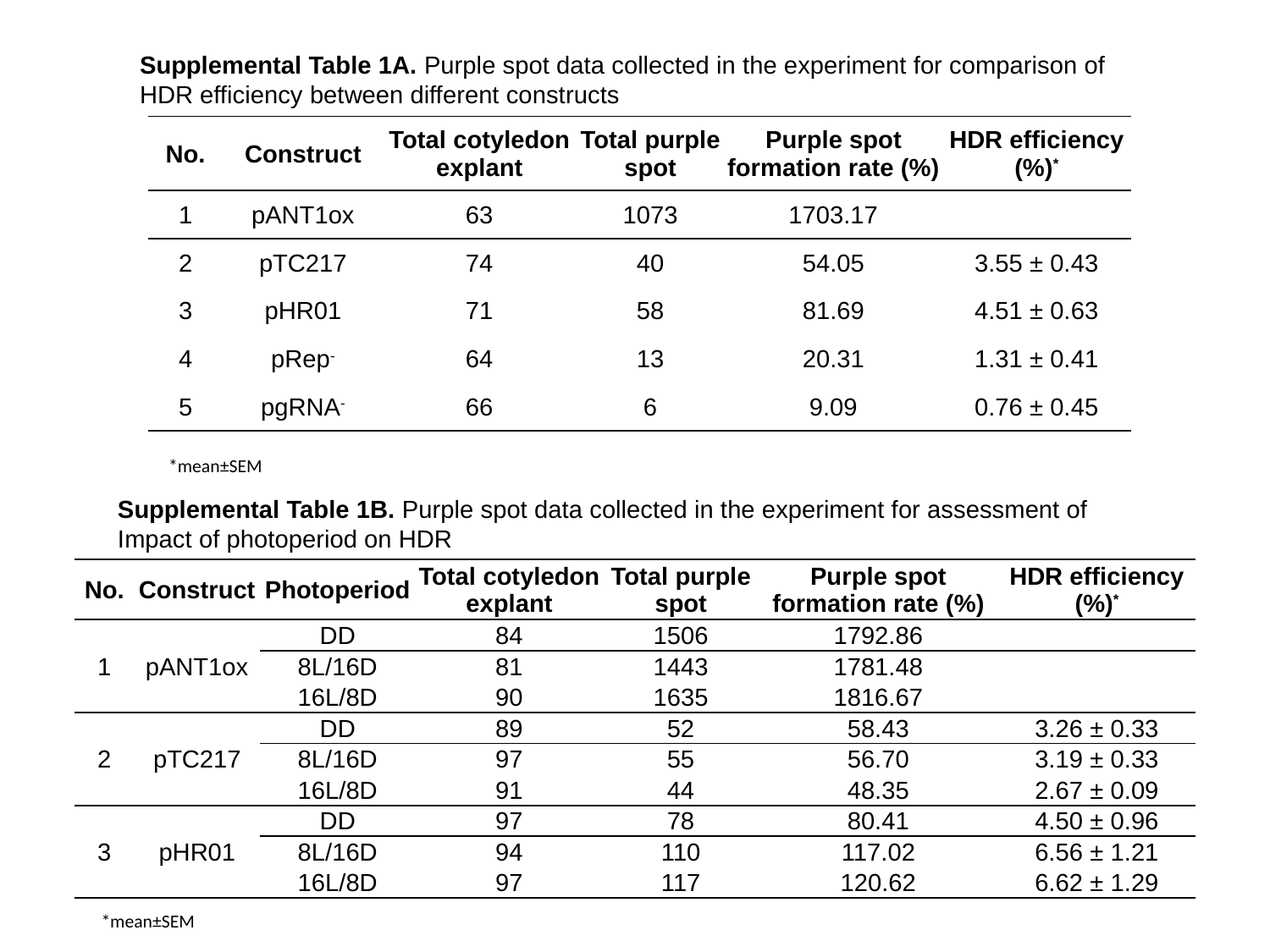

Supplemental Table 1A. Purple spot data collected in the experiment for comparison of HDR efficiency between different constructs
| No. | Construct | Total cotyledon explant | Total purple spot | Purple spot formation rate (%) | HDR efficiency (%)\* |
| --- | --- | --- | --- | --- | --- |
| 1 | pANT1ox | 63 | 1073 | 1703.17 | |
| 2 | pTC217 | 74 | 40 | 54.05 | 3.55 ± 0.43 |
| 3 | pHR01 | 71 | 58 | 81.69 | 4.51 ± 0.63 |
| 4 | pRep- | 64 | 13 | 20.31 | 1.31 ± 0.41 |
| 5 | pgRNA- | 66 | 6 | 9.09 | 0.76 ± 0.45 |
*mean±SEM
Supplemental Table 1B. Purple spot data collected in the experiment for assessment of Impact of photoperiod on HDR
| No. | Construct | Photoperiod | Total cotyledon explant | Total purple spot | Purple spot formation rate (%) | HDR efficiency (%)\* |
| --- | --- | --- | --- | --- | --- | --- |
| 1 | pANT1ox | DD | 84 | 1506 | 1792.86 | |
| | | 8L/16D | 81 | 1443 | 1781.48 | |
| | | 16L/8D | 90 | 1635 | 1816.67 | |
| 2 | pTC217 | DD | 89 | 52 | 58.43 | 3.26 ± 0.33 |
| | | 8L/16D | 97 | 55 | 56.70 | 3.19 ± 0.33 |
| | | 16L/8D | 91 | 44 | 48.35 | 2.67 ± 0.09 |
| 3 | pHR01 | DD | 97 | 78 | 80.41 | 4.50 ± 0.96 |
| | | 8L/16D | 94 | 110 | 117.02 | 6.56 ± 1.21 |
| | | 16L/8D | 97 | 117 | 120.62 | 6.62 ± 1.29 |
*mean±SEM

## Slide 2
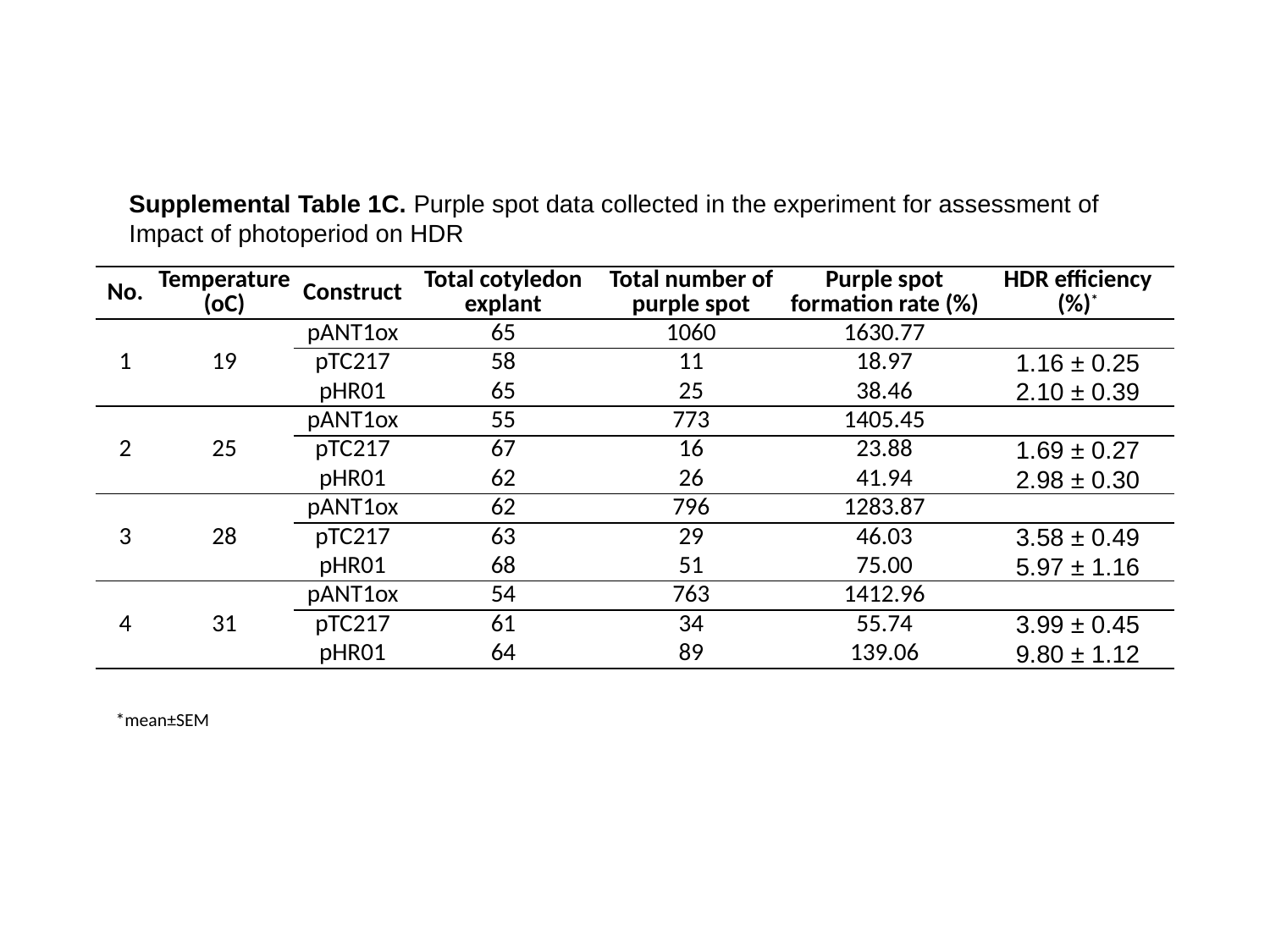

Supplemental Table 1C. Purple spot data collected in the experiment for assessment of Impact of photoperiod on HDR
| No. | Temperature (oC) | Construct | Total cotyledon explant | Total number of purple spot | Purple spot formation rate (%) | HDR efficiency (%)\* |
| --- | --- | --- | --- | --- | --- | --- |
| 1 | 19 | pANT1ox | 65 | 1060 | 1630.77 | |
| | | pTC217 | 58 | 11 | 18.97 | 1.16 ± 0.25 |
| | | pHR01 | 65 | 25 | 38.46 | 2.10 ± 0.39 |
| 2 | 25 | pANT1ox | 55 | 773 | 1405.45 | |
| | | pTC217 | 67 | 16 | 23.88 | 1.69 ± 0.27 |
| | | pHR01 | 62 | 26 | 41.94 | 2.98 ± 0.30 |
| 3 | 28 | pANT1ox | 62 | 796 | 1283.87 | |
| | | pTC217 | 63 | 29 | 46.03 | 3.58 ± 0.49 |
| | | pHR01 | 68 | 51 | 75.00 | 5.97 ± 1.16 |
| 4 | 31 | pANT1ox | 54 | 763 | 1412.96 | |
| | | pTC217 | 61 | 34 | 55.74 | 3.99 ± 0.45 |
| | | pHR01 | 64 | 89 | 139.06 | 9.80 ± 1.12 |
*mean±SEM

## Slide 3
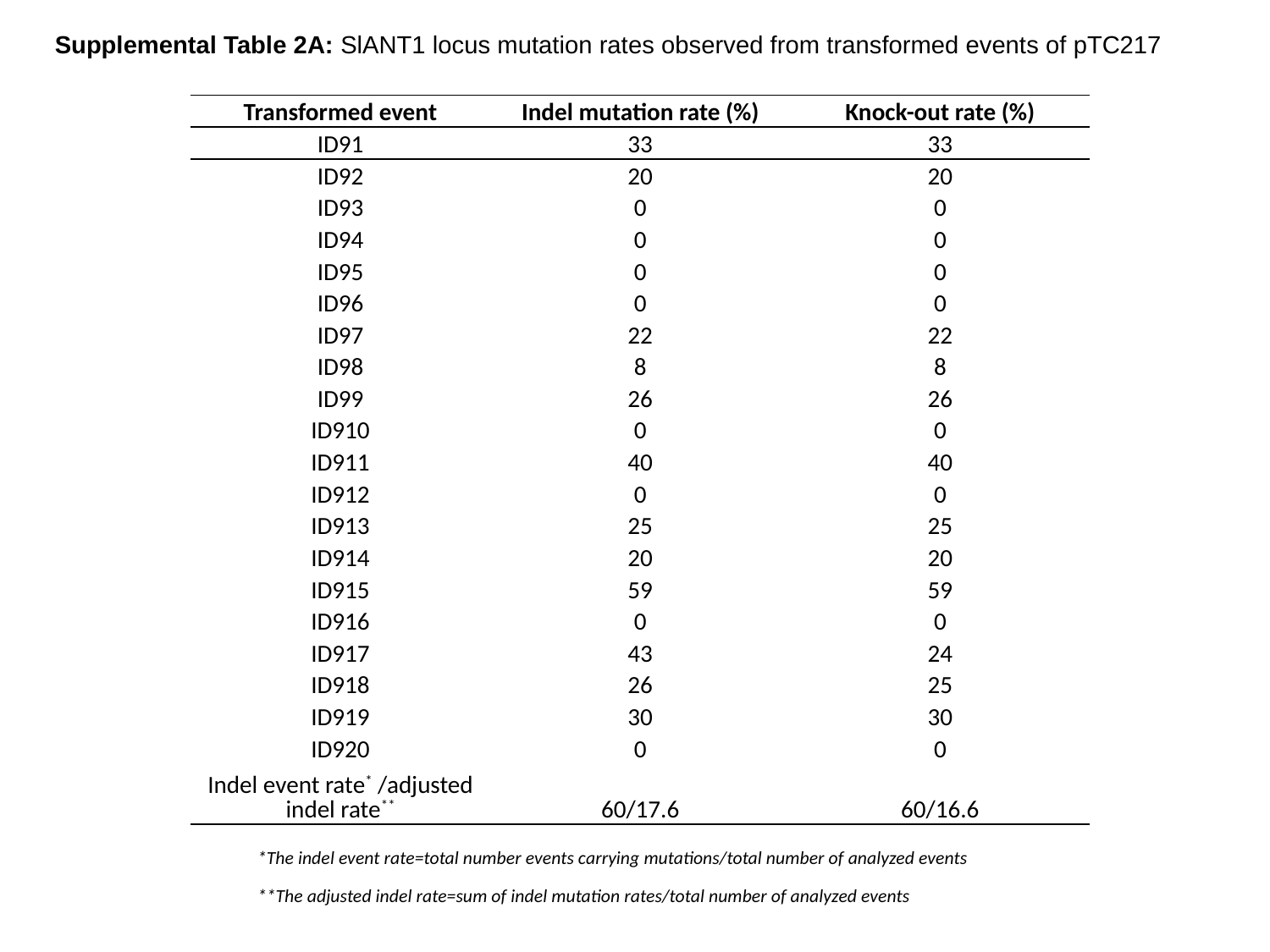

Supplemental Table 2A: SlANT1 locus mutation rates observed from transformed events of pTC217
| Transformed event | Indel mutation rate (%) | Knock-out rate (%) |
| --- | --- | --- |
| ID91 | 33 | 33 |
| ID92 | 20 | 20 |
| ID93 | 0 | 0 |
| ID94 | 0 | 0 |
| ID95 | 0 | 0 |
| ID96 | 0 | 0 |
| ID97 | 22 | 22 |
| ID98 | 8 | 8 |
| ID99 | 26 | 26 |
| ID910 | 0 | 0 |
| ID911 | 40 | 40 |
| ID912 | 0 | 0 |
| ID913 | 25 | 25 |
| ID914 | 20 | 20 |
| ID915 | 59 | 59 |
| ID916 | 0 | 0 |
| ID917 | 43 | 24 |
| ID918 | 26 | 25 |
| ID919 | 30 | 30 |
| ID920 | 0 | 0 |
| Indel event rate\* /adjusted indel rate\*\* | 60/17.6 | 60/16.6 |
*The indel event rate=total number events carrying mutations/total number of analyzed events
**The adjusted indel rate=sum of indel mutation rates/total number of analyzed events

## Slide 4
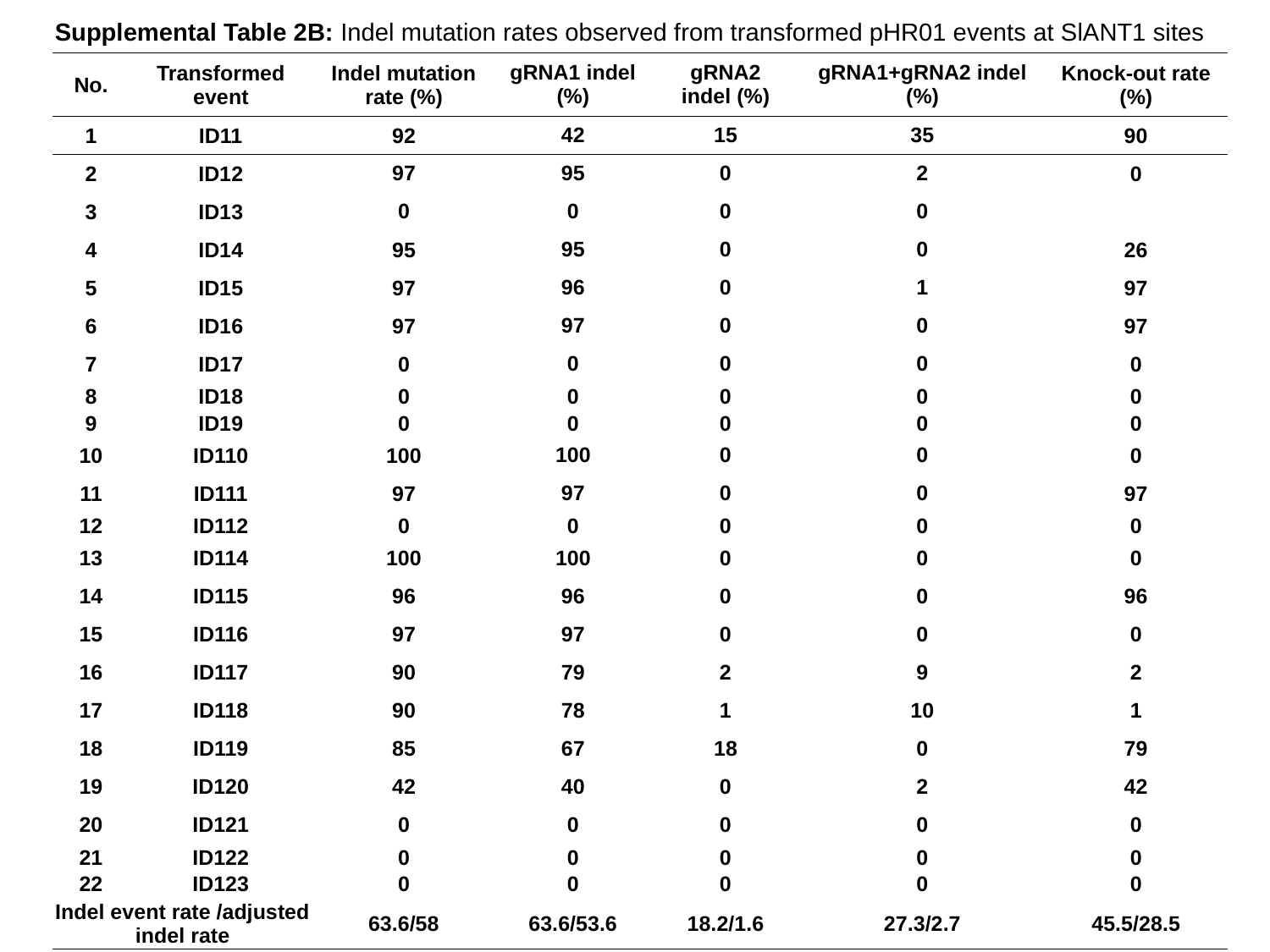

Supplemental Table 2B: Indel mutation rates observed from transformed pHR01 events at SlANT1 sites
| No. | Transformed event | Indel mutation rate (%) | gRNA1 indel (%) | gRNA2 indel (%) | gRNA1+gRNA2 indel (%) | Knock-out rate (%) |
| --- | --- | --- | --- | --- | --- | --- |
| 1 | ID11 | 92 | 42 | 15 | 35 | 90 |
| 2 | ID12 | 97 | 95 | 0 | 2 | 0 |
| 3 | ID13 | 0 | 0 | 0 | 0 | |
| 4 | ID14 | 95 | 95 | 0 | 0 | 26 |
| 5 | ID15 | 97 | 96 | 0 | 1 | 97 |
| 6 | ID16 | 97 | 97 | 0 | 0 | 97 |
| 7 | ID17 | 0 | 0 | 0 | 0 | 0 |
| 8 | ID18 | 0 | 0 | 0 | 0 | 0 |
| 9 | ID19 | 0 | 0 | 0 | 0 | 0 |
| 10 | ID110 | 100 | 100 | 0 | 0 | 0 |
| 11 | ID111 | 97 | 97 | 0 | 0 | 97 |
| 12 | ID112 | 0 | 0 | 0 | 0 | 0 |
| 13 | ID114 | 100 | 100 | 0 | 0 | 0 |
| 14 | ID115 | 96 | 96 | 0 | 0 | 96 |
| 15 | ID116 | 97 | 97 | 0 | 0 | 0 |
| 16 | ID117 | 90 | 79 | 2 | 9 | 2 |
| 17 | ID118 | 90 | 78 | 1 | 10 | 1 |
| 18 | ID119 | 85 | 67 | 18 | 0 | 79 |
| 19 | ID120 | 42 | 40 | 0 | 2 | 42 |
| 20 | ID121 | 0 | 0 | 0 | 0 | 0 |
| 21 | ID122 | 0 | 0 | 0 | 0 | 0 |
| 22 | ID123 | 0 | 0 | 0 | 0 | 0 |
| Indel event rate /adjusted indel rate | | 63.6/58 | 63.6/53.6 | 18.2/1.6 | 27.3/2.7 | 45.5/28.5 |

## Slide 5
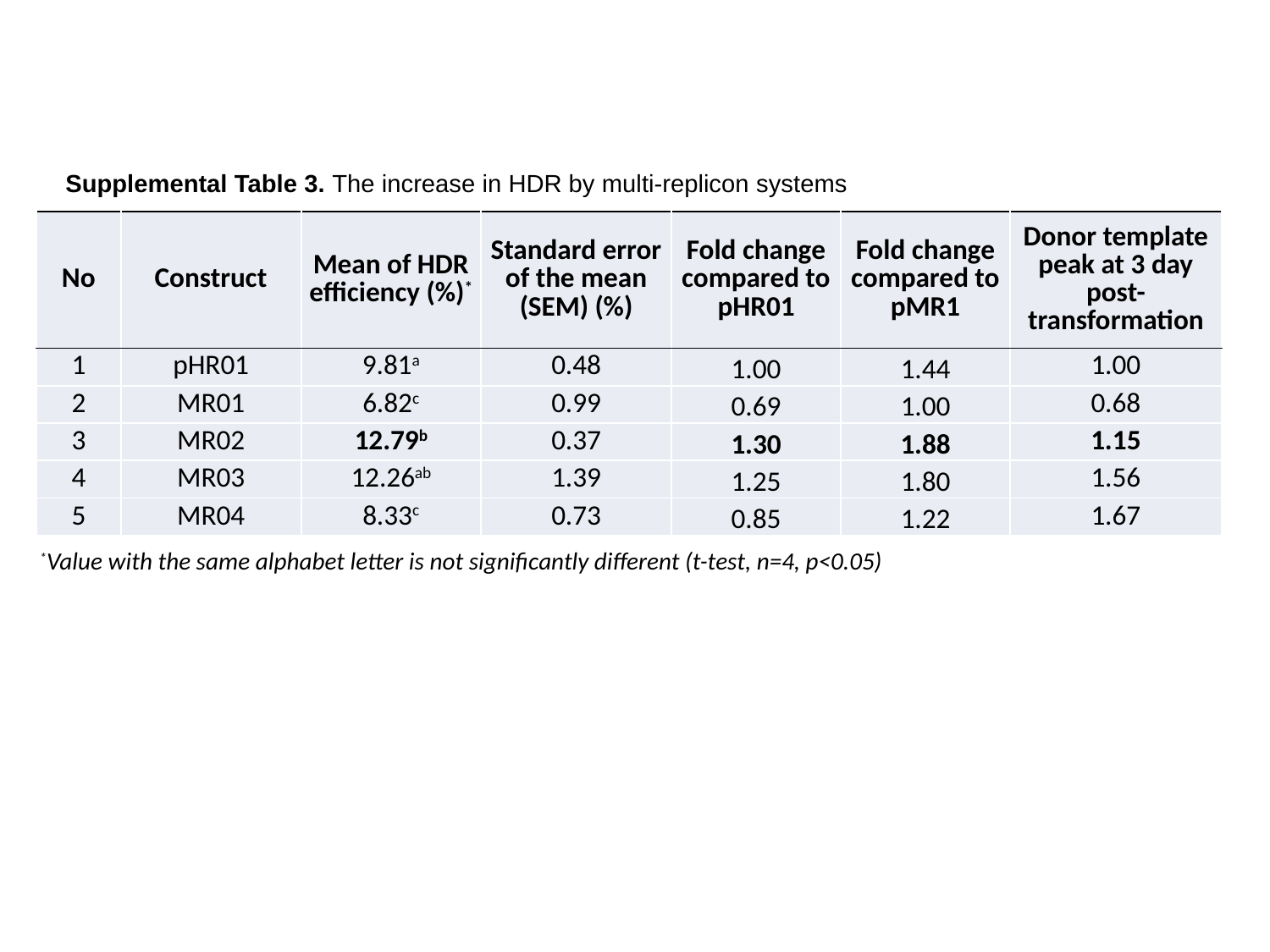

Supplemental Table 3. The increase in HDR by multi-replicon systems
| No | Construct | Mean of HDR efficiency (%)\* | Standard error of the mean (SEM) (%) | Fold change compared to pHR01 | Fold change compared to pMR1 | Donor template peak at 3 day post-transformation |
| --- | --- | --- | --- | --- | --- | --- |
| 1 | pHR01 | 9.81a | 0.48 | 1.00 | 1.44 | 1.00 |
| 2 | MR01 | 6.82c | 0.99 | 0.69 | 1.00 | 0.68 |
| 3 | MR02 | 12.79b | 0.37 | 1.30 | 1.88 | 1.15 |
| 4 | MR03 | 12.26ab | 1.39 | 1.25 | 1.80 | 1.56 |
| 5 | MR04 | 8.33c | 0.73 | 0.85 | 1.22 | 1.67 |
*Value with the same alphabet letter is not significantly different (t-test, n=4, p<0.05)

## Slide 6
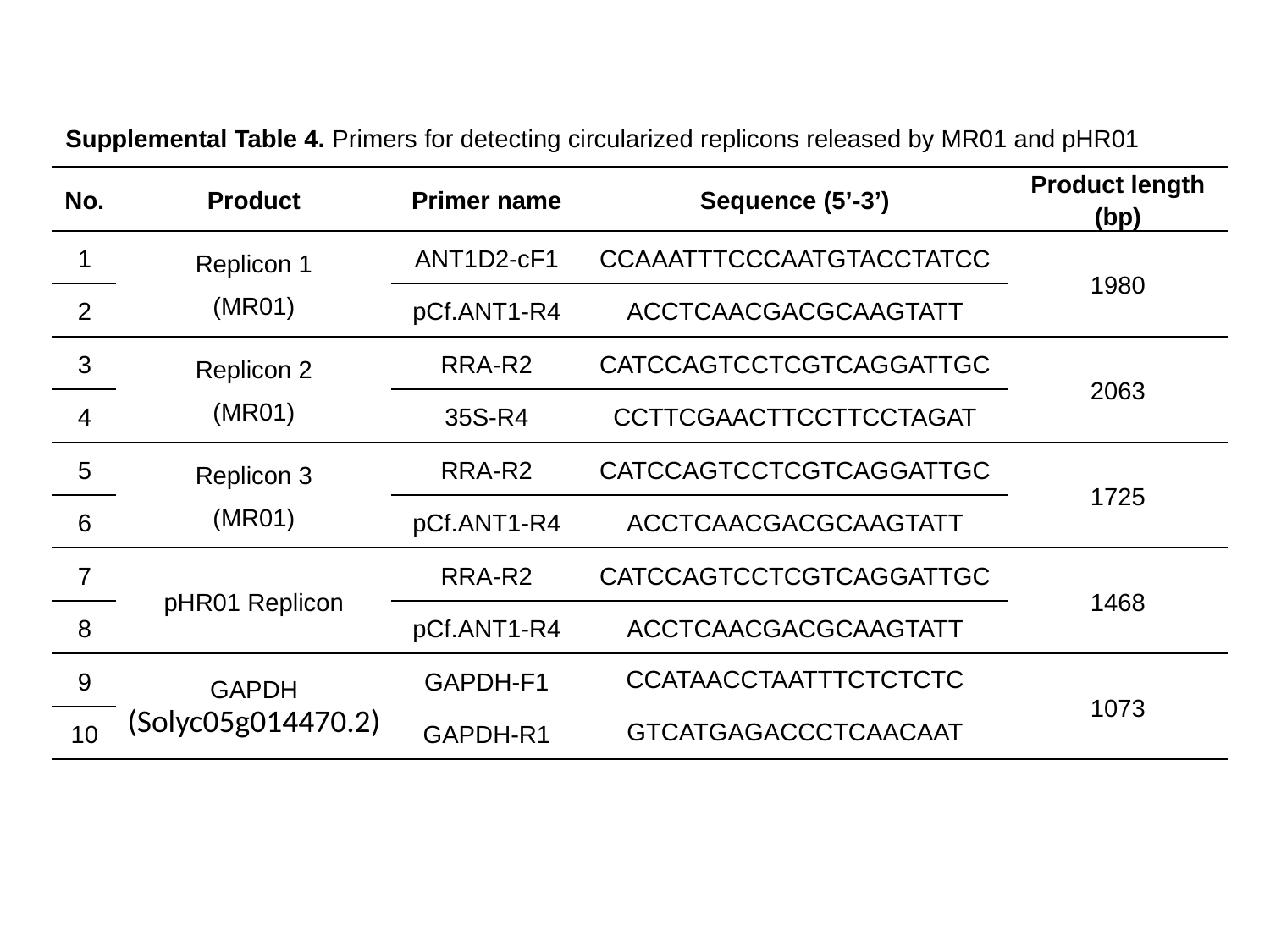

Supplemental Table 4. Primers for detecting circularized replicons released by MR01 and pHR01
| No. | Product | Primer name | Sequence (5’-3’) | Product length (bp) |
| --- | --- | --- | --- | --- |
| 1 | Replicon 1 (MR01) | ANT1D2-cF1 | CCAAATTTCCCAATGTACCTATCC | 1980 |
| 2 | | pCf.ANT1-R4 | ACCTCAACGACGCAAGTATT | |
| 3 | Replicon 2 (MR01) | RRA-R2 | CATCCAGTCCTCGTCAGGATTGC | 2063 |
| 4 | | 35S-R4 | CCTTCGAACTTCCTTCCTAGAT | |
| 5 | Replicon 3 (MR01) | RRA-R2 | CATCCAGTCCTCGTCAGGATTGC | 1725 |
| 6 | | pCf.ANT1-R4 | ACCTCAACGACGCAAGTATT | |
| 7 | pHR01 Replicon | RRA-R2 | CATCCAGTCCTCGTCAGGATTGC | 1468 |
| 8 | | pCf.ANT1-R4 | ACCTCAACGACGCAAGTATT | |
| 9 | GAPDH (Solyc05g014470.2) | GAPDH-F1 | CCATAACCTAATTTCTCTCTC | 1073 |
| 10 | | GAPDH-R1 | GTCATGAGACCCTCAACAAT | |

## Slide 7
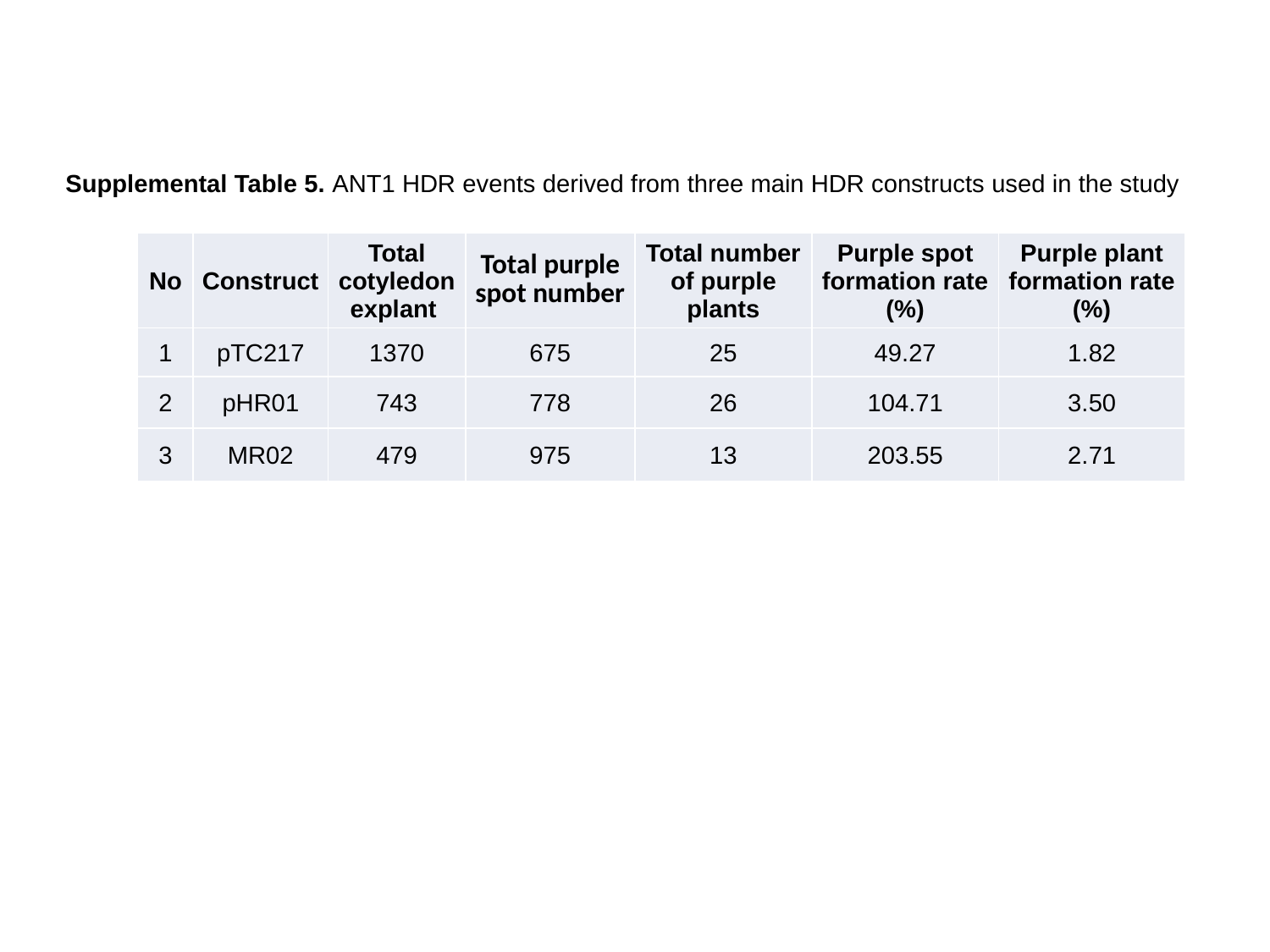

Supplemental Table 5. ANT1 HDR events derived from three main HDR constructs used in the study
| No | Construct | Total cotyledon explant | Total purple spot number | Total number of purple plants | Purple spot formation rate (%) | Purple plant formation rate (%) |
| --- | --- | --- | --- | --- | --- | --- |
| 1 | pTC217 | 1370 | 675 | 25 | 49.27 | 1.82 |
| 2 | pHR01 | 743 | 778 | 26 | 104.71 | 3.50 |
| 3 | MR02 | 479 | 975 | 13 | 203.55 | 2.71 |

## Slide 8
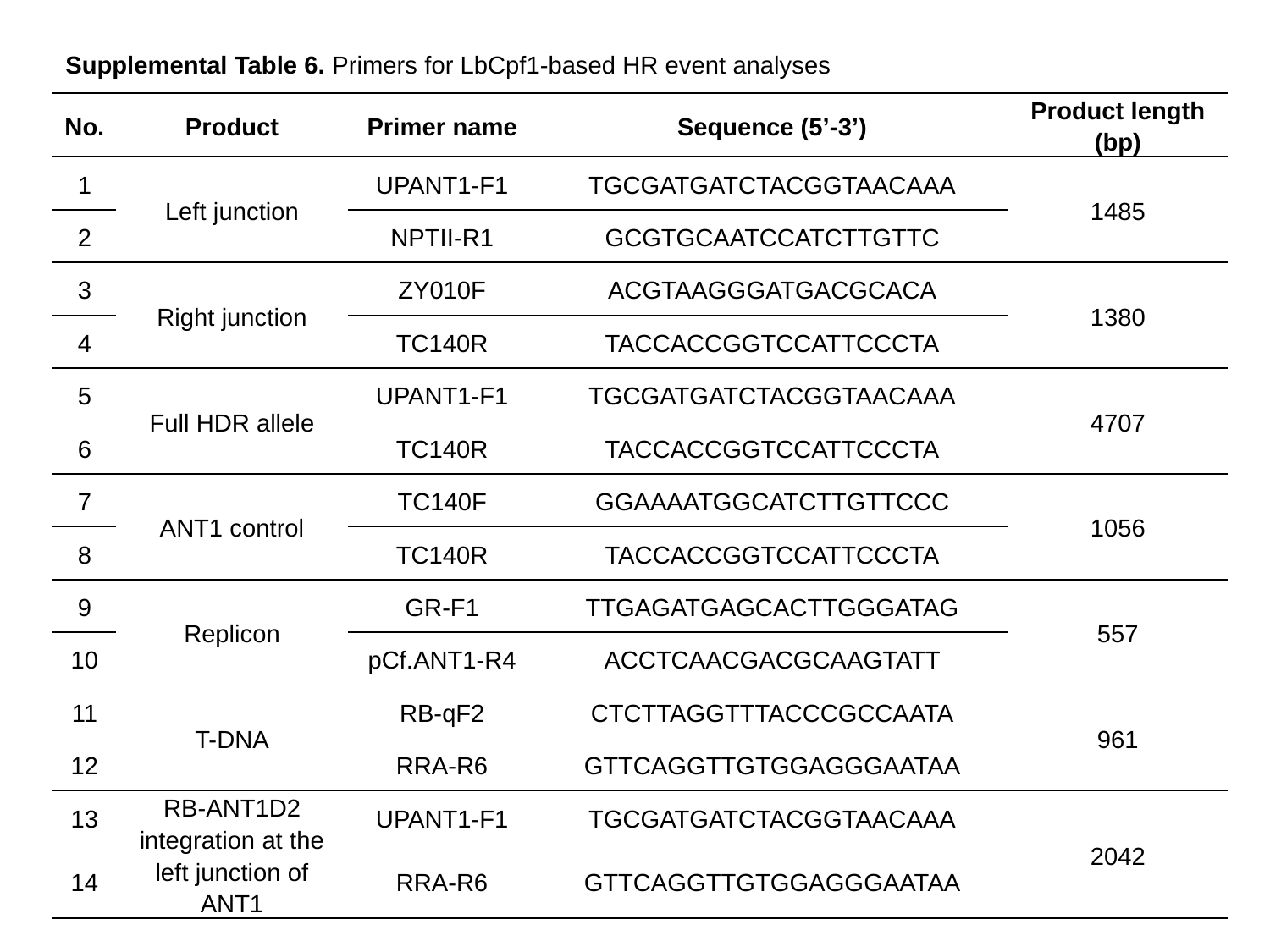

Supplemental Table 6. Primers for LbCpf1-based HR event analyses
| No. | Product | Primer name | Sequence (5’-3’) | Product length (bp) |
| --- | --- | --- | --- | --- |
| 1 | Left junction | UPANT1-F1 | TGCGATGATCTACGGTAACAAA | 1485 |
| 2 | | NPTII-R1 | GCGTGCAATCCATCTTGTTC | |
| 3 | Right junction | ZY010F | ACGTAAGGGATGACGCACA | 1380 |
| 4 | | TC140R | TACCACCGGTCCATTCCCTA | |
| 5 | Full HDR allele | UPANT1-F1 | TGCGATGATCTACGGTAACAAA | 4707 |
| 6 | | TC140R | TACCACCGGTCCATTCCCTA | |
| 7 | ANT1 control | TC140F | GGAAAATGGCATCTTGTTCCC | 1056 |
| 8 | | TC140R | TACCACCGGTCCATTCCCTA | |
| 9 | Replicon | GR-F1 | TTGAGATGAGCACTTGGGATAG | 557 |
| 10 | | pCf.ANT1-R4 | ACCTCAACGACGCAAGTATT | |
| 11 | T-DNA | RB-qF2 | CTCTTAGGTTTACCCGCCAATA | 961 |
| 12 | | RRA-R6 | GTTCAGGTTGTGGAGGGAATAA | |
| 13 | RB-ANT1D2 integration at the left junction of ANT1 | UPANT1-F1 | TGCGATGATCTACGGTAACAAA | 2042 |
| 14 | | RRA-R6 | GTTCAGGTTGTGGAGGGAATAA | |

## Slide 9
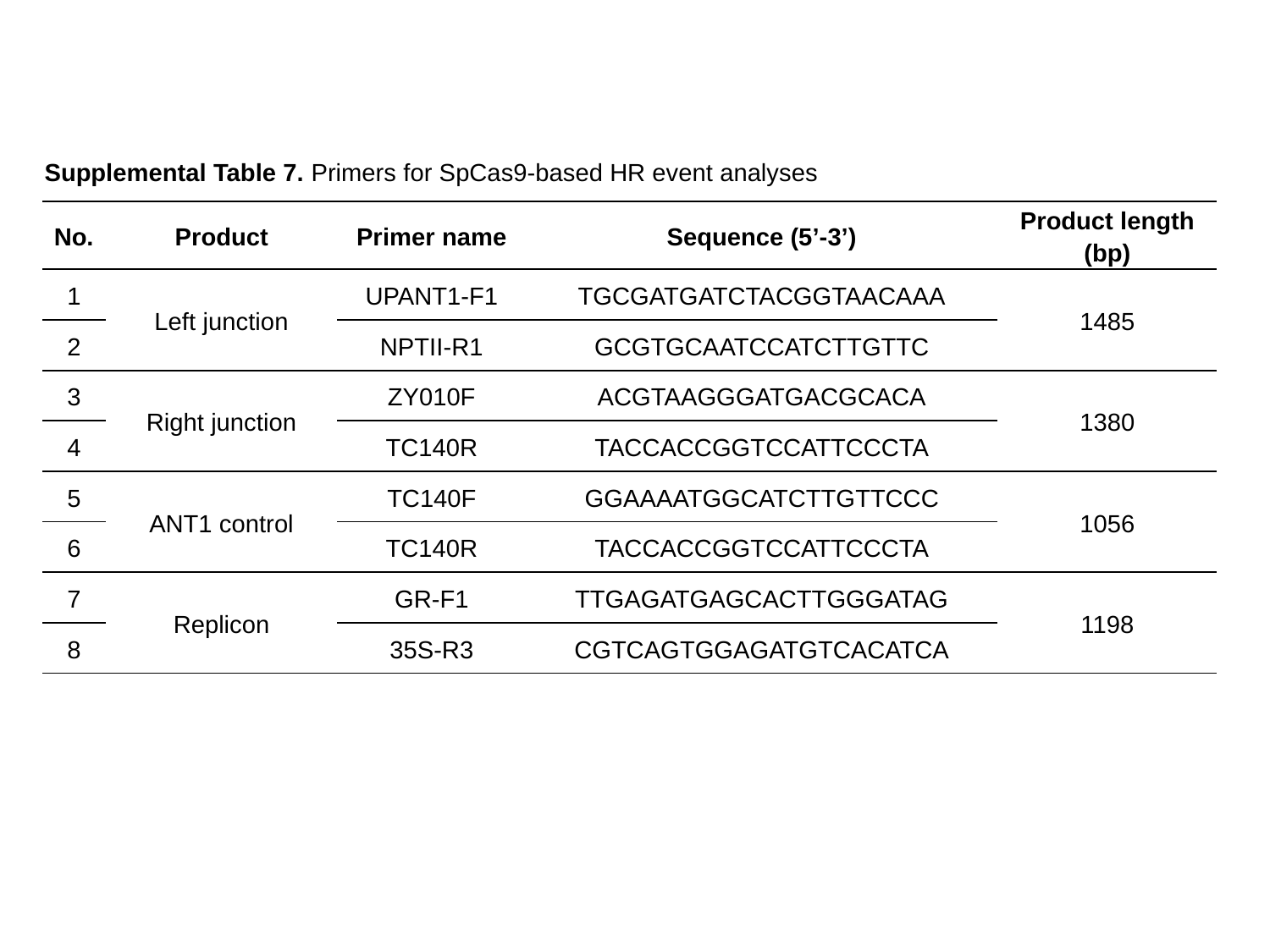

Supplemental Table 7. Primers for SpCas9-based HR event analyses
| No. | Product | Primer name | Sequence (5’-3’) | Product length (bp) |
| --- | --- | --- | --- | --- |
| 1 | Left junction | UPANT1-F1 | TGCGATGATCTACGGTAACAAA | 1485 |
| 2 | | NPTII-R1 | GCGTGCAATCCATCTTGTTC | |
| 3 | Right junction | ZY010F | ACGTAAGGGATGACGCACA | 1380 |
| 4 | | TC140R | TACCACCGGTCCATTCCCTA | |
| 5 | ANT1 control | TC140F | GGAAAATGGCATCTTGTTCCC | 1056 |
| 6 | | TC140R | TACCACCGGTCCATTCCCTA | |
| 7 | Replicon | GR-F1 | TTGAGATGAGCACTTGGGATAG | 1198 |
| 8 | | 35S-R3 | CGTCAGTGGAGATGTCACATCA | |

## Slide 10
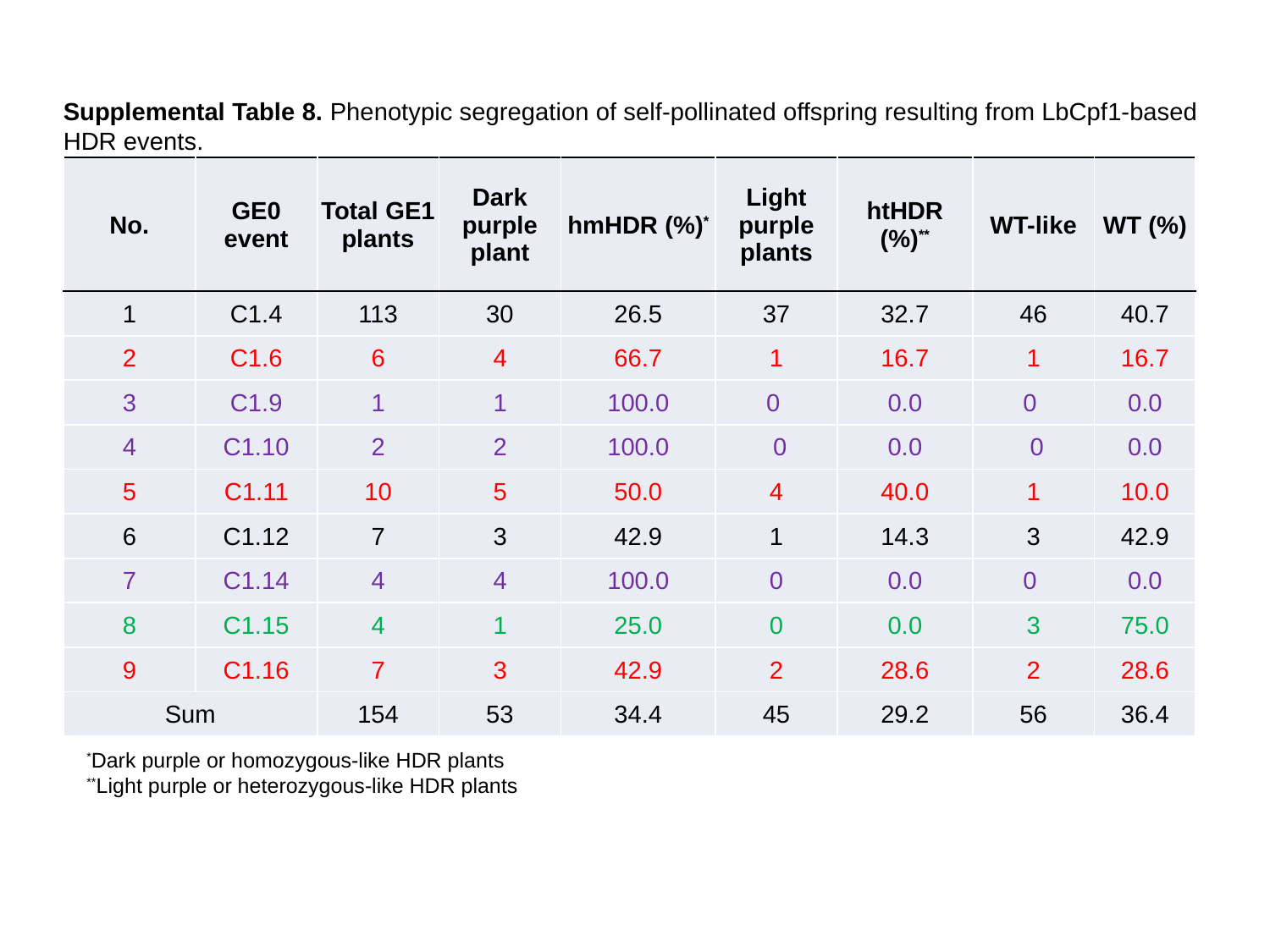

Supplemental Table 8. Phenotypic segregation of self-pollinated offspring resulting from LbCpf1-based HDR events.
| No. | GE0 event | Total GE1 plants | Dark purple plant | hmHDR (%)\* | Light purple plants | htHDR (%)\*\* | WT-like | WT (%) |
| --- | --- | --- | --- | --- | --- | --- | --- | --- |
| 1 | C1.4 | 113 | 30 | 26.5 | 37 | 32.7 | 46 | 40.7 |
| 2 | C1.6 | 6 | 4 | 66.7 | 1 | 16.7 | 1 | 16.7 |
| 3 | C1.9 | 1 | 1 | 100.0 | 0 | 0.0 | 0 | 0.0 |
| 4 | C1.10 | 2 | 2 | 100.0 | 0 | 0.0 | 0 | 0.0 |
| 5 | C1.11 | 10 | 5 | 50.0 | 4 | 40.0 | 1 | 10.0 |
| 6 | C1.12 | 7 | 3 | 42.9 | 1 | 14.3 | 3 | 42.9 |
| 7 | C1.14 | 4 | 4 | 100.0 | 0 | 0.0 | 0 | 0.0 |
| 8 | C1.15 | 4 | 1 | 25.0 | 0 | 0.0 | 3 | 75.0 |
| 9 | C1.16 | 7 | 3 | 42.9 | 2 | 28.6 | 2 | 28.6 |
| Sum | | 154 | 53 | 34.4 | 45 | 29.2 | 56 | 36.4 |
*Dark purple or homozygous-like HDR plants
**Light purple or heterozygous-like HDR plants

## Slide 11
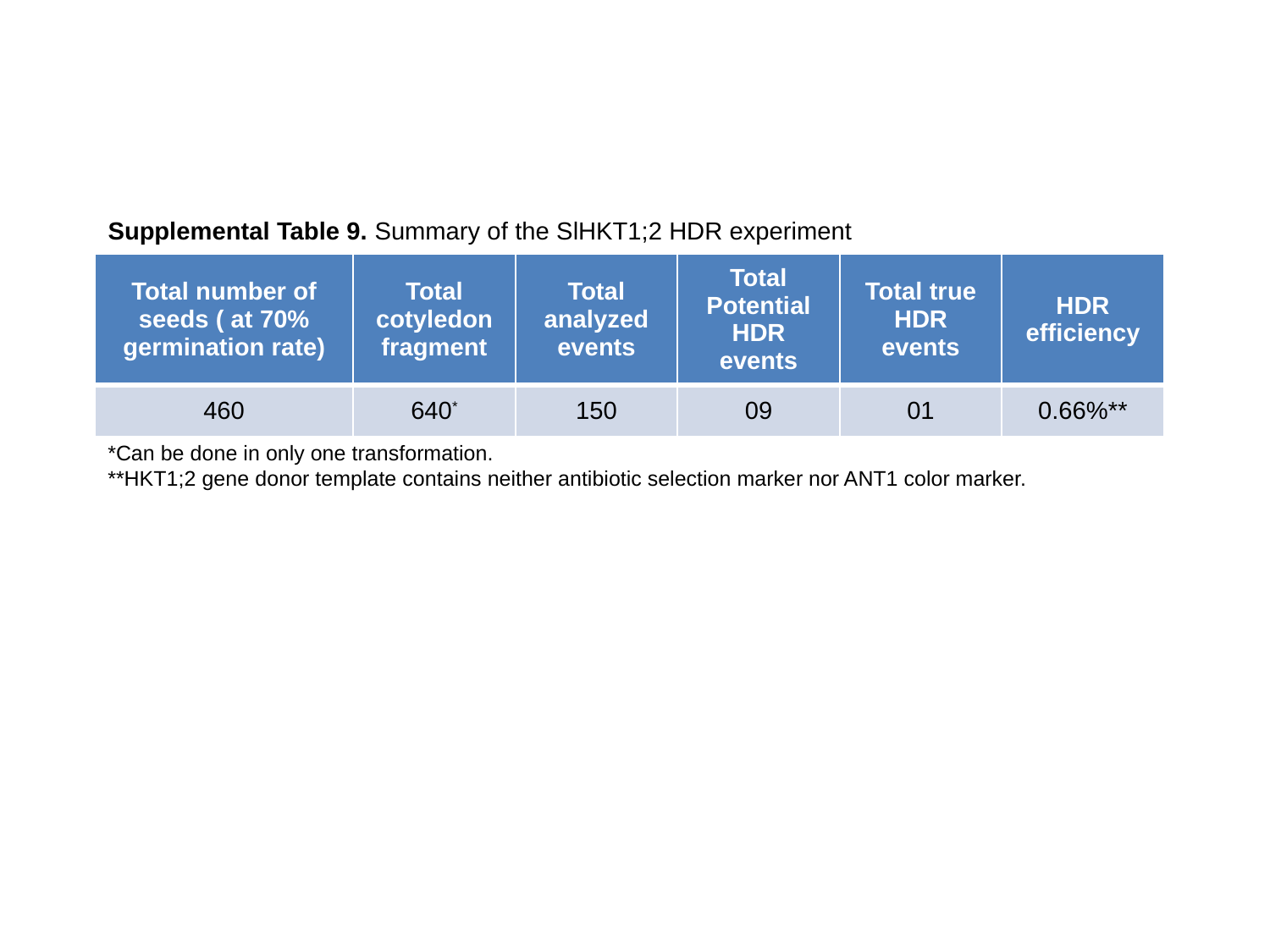

Supplemental Table 9. Summary of the SlHKT1;2 HDR experiment
| Total number of seeds ( at 70% germination rate) | Total cotyledon fragment | Total analyzed events | Total Potential HDR events | Total true HDR events | HDR efficiency |
| --- | --- | --- | --- | --- | --- |
| 460 | 640\* | 150 | 09 | 01 | 0.66%\*\* |
*Can be done in only one transformation.
**HKT1;2 gene donor template contains neither antibiotic selection marker nor ANT1 color marker.

## Slide 12
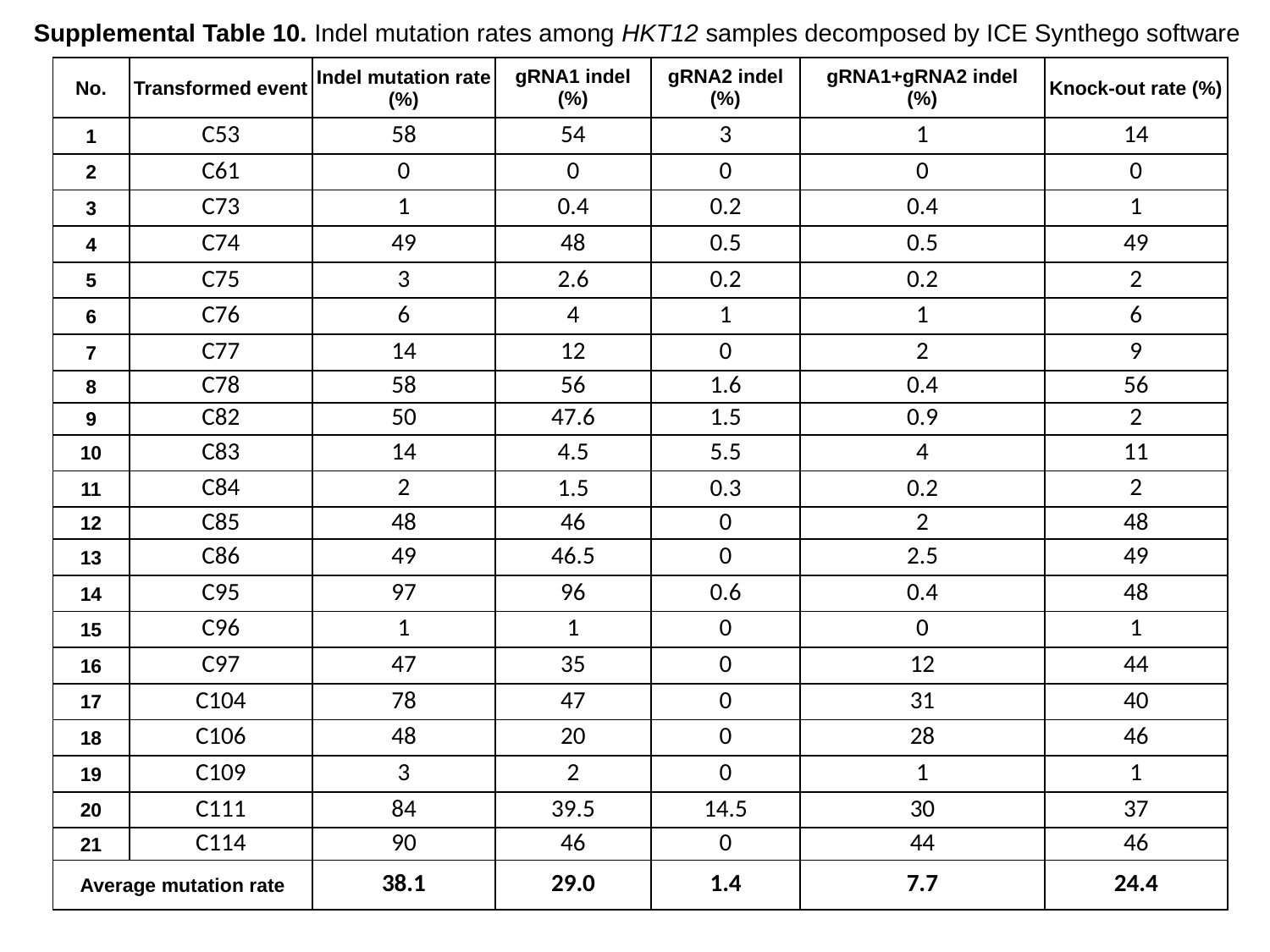

Supplemental Table 10. Indel mutation rates among HKT12 samples decomposed by ICE Synthego software
| No. | Transformed event | Indel mutation rate (%) | gRNA1 indel (%) | gRNA2 indel (%) | gRNA1+gRNA2 indel (%) | Knock-out rate (%) |
| --- | --- | --- | --- | --- | --- | --- |
| 1 | C53 | 58 | 54 | 3 | 1 | 14 |
| 2 | C61 | 0 | 0 | 0 | 0 | 0 |
| 3 | C73 | 1 | 0.4 | 0.2 | 0.4 | 1 |
| 4 | C74 | 49 | 48 | 0.5 | 0.5 | 49 |
| 5 | C75 | 3 | 2.6 | 0.2 | 0.2 | 2 |
| 6 | C76 | 6 | 4 | 1 | 1 | 6 |
| 7 | C77 | 14 | 12 | 0 | 2 | 9 |
| 8 | C78 | 58 | 56 | 1.6 | 0.4 | 56 |
| 9 | C82 | 50 | 47.6 | 1.5 | 0.9 | 2 |
| 10 | C83 | 14 | 4.5 | 5.5 | 4 | 11 |
| 11 | C84 | 2 | 1.5 | 0.3 | 0.2 | 2 |
| 12 | C85 | 48 | 46 | 0 | 2 | 48 |
| 13 | C86 | 49 | 46.5 | 0 | 2.5 | 49 |
| 14 | C95 | 97 | 96 | 0.6 | 0.4 | 48 |
| 15 | C96 | 1 | 1 | 0 | 0 | 1 |
| 16 | C97 | 47 | 35 | 0 | 12 | 44 |
| 17 | C104 | 78 | 47 | 0 | 31 | 40 |
| 18 | C106 | 48 | 20 | 0 | 28 | 46 |
| 19 | C109 | 3 | 2 | 0 | 1 | 1 |
| 20 | C111 | 84 | 39.5 | 14.5 | 30 | 37 |
| 21 | C114 | 90 | 46 | 0 | 44 | 46 |
| Average mutation rate | | 38.1 | 29.0 | 1.4 | 7.7 | 24.4 |
