# Supplementary material for: Highly efficient homology‐directed repair using CRISPR/Cpf1‐geminiviral replicon in tomato
Source: Plant Biotechnol J. 2020 Apr 1;18(10):2133–43. doi: 10.1111/pbi.13373 (PMC7540044; doi:10.1111/pbi.13373)
Supplement: Supplementary file 3 — Data S1 Sequences used in the study. [file PBI-18-2133-s007.docx]

**SEQUENCES USED IN THE STUDY**

- **Single replicon system with Golden gate level 2 acceptor sites**

TGATGGGCTGCCTGTATCGAGTGGTGATTTTGTGCCGAGCTGCCGGTCGGGGAGCTGTTGGCTGGCTGGTGGCAGGATATATTGTGGTGTAAACAAATTGACGCTTAGACAACTTAATAACACATTGCGGACGTTTTTAATGTACTGGGGTTGAACACTCTGTGCCTAGCAGAAGGCATGTTGTTGTGACTCCGAGGGGTTGCCTCAAACTCTATCTTATAACCGGCGTGGAGGCATGGAGGCAGGGGTATTTTGGTCATTTTAATAGATAGTGGAAAATGACGTGGAATTTACTTAAAGACGAAGTCTTTGCGACAAGGGGGGGCCCACGCCGAATTTAATATTACCGGCGTGGCCCCCCCTTATCGCGAGTGCTTTAGCACGAGCGGTCCAGATTTAAAGTAGAAAATTTCCCGCCCACTAGGGTTAAAGGTGTTCACACTATAAAAGCATATACGATGTGATGGTATTTGATGGAGCGTATATTGTATCAGGTATTTCCGTTGGATACGAATTATTCGTACGACCCTCGGCGCGTGCCTTGTCTTCCAGTGGCAAGTGGAAGACAAGGGAAGCTTTGGGTACGTCACGTGGCTCGAGCGCGTAGTCCTCGGTAATATCGCAGAACAAAAGTACCTGATATCGAGTGTACTTCAAGTCAGTGGGAAATCAATAAAATGATTATTTTATGAATATATTTCATTGTGCAAGTAGATAGAAATTACATATGTTACATAACACACGAAATAAACAAAAAAAGACAATCCAAAAACAAACACCCCAAAAAAAATAATCACTTTAGATAAACTCGTATGAGGAGAGGCACGTTGCCTCAGTGACTCGACGATTCCCGAGCAAAAAAAGTCTCCCCGTCACACATGTAGTGGGTGACGCAATTATCTTTAAAGTAATCCTTCTGTTGACTTGTCATTGATAACATCCAGTCCTCGTCAGGATTGCAAAGAATTATAGAAGGGATCCCACCTTTTATTTTCTTCTTTTTTCCATATTTAGGGTTGACAGTGAAATCAGACTGGCAACCTATTAATTGCTTCCACAATGGGACGAACTTGAAGGGGATGTCGTCGATGATATTATAGGTGGCGTGTTCATCGTAGTTGGTGAAATCGATGGTACCGTTCCAATAGTTGTGTCGTCCGAGACTTCTAGCCCAGGTGGTCTTTCCGGTACGAGTTGGTCCGCAGATGTAGAGGCTGGGGTGTCGGATTCCATTCCTTCCATTGTCCTTGTTAAATCGGCCATCCATTCAAGGTCAGATTGAGCTTGTTGGTATGAGACAGGATGTATGTAAGTATAAGCGTCTATGCTTACATGGTATAGATGGGTTTCCCTCCAGGAGTGTAGATCTTCGTGGCAGCGAAGATCTGATTCTGTGAAGGGCGACACATACGGTTCAGGTTGTGGAGGGAATAATTTGTTGGCTGAATATTCCAGCCATTGAAGCTTTGTTGCCCATTCATGAGGGAATTCTTCCTTGATCATGTCAAGATATTCCTCCTTAGACGTTGCAGTCTGGATAATAGTTCTCCATCGTGCGTCAGATTTGCGAGGAGAAACCTTATGATCTCGGAAATCTCCTCTGGTTTTAATATCTCCGTCCTTTGATATGTAATCAAGGACTTGTTTAGAGTTTCTAGCTGGCTGGATATTAGGGTGATTTCCTTCAAAATCGAAAAAAGAAGGATCCCTAATACAAGGTTTTTTATCAAGCTGGAGAAGAGCATGATAGTGGGTAGTGCCATCTTGATGAAGCTCAGAAGCAACACCAAGGAAGAAAATAAGAAAAGGTGTGAGTTTCTCCCAGAGAAACTGGAATAAATCATCTCTTTGAGATGAGCACTTGGGATAGGTAAGGAAAACATATTTAGATTGGAGTCTGAAGTTCTTACTAGCAGAAGGCATTTTGGGAGTTATTGTGACTCCGAGGGGTTGCCTCAAACTCTATCTTATAACCGGCGTGGAGGCATGGAGGCAGGGGTATTTTGGTCATTTTAATAGATAGTGGAAAATGACGTGGAATTTACTTAAAGACGAAGTCTTTGCGACAAGGGGGGGCCCACGCCGAATTTAATATTACCGGCGTGGCCCCCCCTTATCGCGAGTGCTTTAGCACGAGCGGTCCAGATTTAAAGTAGAAAATTTCCCGCCCACTAGGGTTAAAGGTGTTCACACTATAAAAGCATATACGATGTGATGGTATTTGATGGAGCGTATATTGTATCAGGTATTTCCGTTGGATACGAATTATTCGTACGACCCTCACTAGAGGATGCACATGTGACCGAGGGACACGAAGTGATCCGTTTAAACTATCAGTGTTTGACAGGATATATTGGCGGGTAAACCTAAGAGAAAAGAGCGTTTATTAGAATAATCGGATATTTAAAAGGGCGTGAAAAGGTTTATCCGTTCGTCCATTTGTATGTGCC

Orange font: LB; red font: LIR sequences; purple font: RB; olive green: SIR; dark blue font: reverse complement sequence of Rep/RepA; yellow highlighted font: Golden gate level 2 cloning sites (BpiI) with TGCC-GGGA flanking sites; black font: spacer sequences.

- **In pHR01**
- The dual crRNA expression cassette:

TGATCAAAAGTCCCACATCGATCAGGTGATATATAGCAGCTTAGTTTATATAATGATAGAGTCGACATAGCGATTGTAATTTCTACTAAGTGTAGATTAGAAGGCTCTCTACAAGTTGGTTAATTTCTACTAAGTGTAGATATACACCTTTTAGGCACGTGTATTTTTTT

Red font: AtU6 promoter; green font: LbCpf1 scaffold; purple font: LbCpf1_gRNA1; orange font: LbCpf1_gRNA2; black G: transcription start; black TTTTTT: termination sequence.

- ANT1D2 donor sequence

TTGGTCCCCAAGTACTTAAATTGGTCCCCAAGTACTTACCACAACACTTGTCGGTGAGATTATTTAATGCTGATTAGATTAGACAAAAATTAATTAGTTTTGAGTAGTGGCGTAAGTGTAAATAATTAGTCTCTTTTTTAACTTAGAAAATAGTTTAATCCTTAGTATAAATAGTCAAAATCACTGGAATGAAAAACAGTTTTTAATTTTTCCAAATTTGATTCTGATACCATGTTAAATTCGTGGTTCAAAATCACTGCAATGAAAAGAGCAATATTGTTTAACTTTTTTTAGGAAAATCGAATTGATTTATAGTCAGTTGATATAGAGTGAATACATAAGGAACATATACAGTTGATACAATTGTATAATTCGTTCATACACTTAATACAAAGTGAACCCACAAGGAACATATACACTTAATATAATTGTATTCCTTGATACAAACCAATTTTGTTCGTGTCTCTACTCTCTATTTCAATTTCGCTTGACTCTTTACTTTTTCTAATATGTAGCTATAAATCGTAATTAAACAATACTATATCTCTAAATCTCTTATTAAGCTCAAACTATGGTCATATTCGAAAAAATCCTTTTAAATATTGGTCCCCTCTCACGATTAATGATAGTTATAACTAACATTCAAATTTTAGTTGTACTTGACATCTAAAACTTAAAAAATAGTACAAGTTAACTTTTTCTTTTTTTTAAAAAAAGGAAATACTTGTATTTATTTTTTTAATATATAGTTATATTTTTGGTTATTTGAAAATACTTGATCTGTCATGTATGCTCAGTTAAATATCGTCACATTATAGAGAAAAAAGTAATAGGAGAAAAAAATTAAAAATTATTTCGAAAAATCAAAATTTTTTTTTGATTGAAATGAAAGATGGGTTTCCCAATCGAGGCTGGCAGGATAGGTACATTGGGAAATTTGGATTTGTGTGTTGAAAATGATTGTTCAATTTGGCTTTTATAACATTTGTCGTTTATAAGGTGTAGAAGGCTCTCTACAAGTTGGTAGTTACAATTTAATACACCTGAATTCGGATCCGGAGCGGAGAATTAAGGGAGTCACGTTATGACCCCCGCCGATGACGCGGGACAAGCCGTTTTACGTTTGGAACTGACAGAACCGCAACGTTGAAGGAGCCACTGAGCCGCGGGTTTCTGGAGTTTAATGAGCTAAGCACATACGTCAGAAACCATTATTGCGCGTTCAAAAGTCGCCTAAGGTCACTATCAGCTAGCAAATATTTCTTGTCAAAAATGCTCCACTGACGTTCCATAAATTCCCCTCGGTATCCAATTAGAGTCTCATATTCACTCTCCTATTTTTACAACAATTACCAACAACAACAAACAACAAACAACATTACAATTACATTTACAATTACCATGGTTGAACAAGATGGATTGCACGCAGGTTCTCCGGCCGCTTGGGTGGAGAGGCTATTCGGCTATGACTGGGCACAACAGACAATCGGCTGCTCTGATGCCGCCGTGTTCCGGCTGTCAGCGCAGGGGCGCCCGGTTCTTTTTGTCAAGACCGACCTGTCCGGTGCCCTGAATGAACTGCAGGACGAGGCAGCGCGGCTATCGTGGCTGGCCACGACGGGCGTTCCTTGCGCAGCTGTGCTCGACGTTGTCACTGAAGCGGGAAGGGACTGGCTGCTATTGGGCGAAGTGCCGGGGCAGGATCTCCTGTCATCTCACCTTGCTCCTGCCGAGAAAGTATCCATCATGGCTGATGCAATGCGGCGGCTGCATACGCTTGATCCGGCTACCTGCCCATTCGACCACCAAGCGAAACATCGCATCGAGCGAGCACGTACTCGGATGGAAGCCGGTCTTGTCGATCAGGATGATCTGGACGAAGAGCATCAGGGGCTCGCGCCAGCCGAACTGTTCGCCAGGCTCAAGGCGCGCATGCCCGACGGCGAGGATCTCGTCGTGACTCATGGCGATGCCTGCTTGCCGAATATCATGGTGGAAAATGGCCGCTTTTCTGGATTCATCGACTGTGGCCGGCTGGGTGTGGCGGACCGCTATCAGGACATAGCGTTGGCTACCCGTGATATTGCTGAAGAGCTTGGCGGCGAATGGGCTGACCGCTTCCTCGTGCTTTACGGTATCGCCGCTCCCGATTCGCAGCGCATCGCCTTCTATCGCCTTCTTGACGAGTTCTTCTGAGCGGGACTCTGGGGTTCGCTAGAGTCCTGCTTTAATGAGATATGCGAGACGCCTATGATCGCATGATATTTGCTTTCAATTCTGTTGTGCACGTTGTAAAAAACCTGAGCATGTGTAGCTCAGATCCTTACCGCCGGTTTCGGTTCATTCTAATGAATATATCACCCGTTACTATCGTATTTTTATGAATAATATTCTCCGTTCAATTTACTGATTGTACCCTACTACTTATATGTACAATATTAAAATGAAAACAATATATTGTGCTGAATAGGTTTATAGCGACATCTATGATAGAGCGCCACAATAACAAACAATTGCGTTTTATTATTACAAATCCAATTTTAAAAAAAGCGGCAGAACCGGTCAAACCTAAAAGACTGATTACATAAATCTTATTCAAATTTCAAAAGTGCCCCAGGGGCTAGTATCTACGACACACCGAGCGGCGAACTAATAACGCTCACTGAAGGGAACTCCGGTTCCCCGCCGGCGCGCATGGGTGAGATTCCTTGAAGTTGAGTATTGGCCGTCCGCTCTACCGAAAGTTACGGGCACCATTCAACCCGGTCCAGCACGGCGGCCGGGTAACCGACTTGCTGCCCCGAGAATTATGCAGCATTTTTTTGGTGTATGTGGGCCCCAAATGAAGTGCAGGTCAAACCTTGACAGTGACGACAAATCGTTGGGCGGGTCCAGGGCGAATTTTGCGACAACATGTCGAGGCTCAGCCGCTGTCAGTCAACATGGTGGAGCACGACACTCTGGTCTACTCCAAAAATGTCAAAGATACAGTCTCAGAAGATCAAAGGGCTATTGAGACTTTTCAACAAAGGATAATTTCGGGAAACCTCCTCGGATTCCATTGCCCAGCTATCTGTCACTTCATCGAAAGGACAGTAGAAAAGGAAGGTGGCTCCTACAAATGCCATCATTGCGATAAAGGAAAGGCTATCATTCAAGATCTCTCTGCCGACAGTGGTCCCAAAGATGGACCCCCACCCACGAGGAGCATCGTGGAAAAAGAAGAGGTTCCAACCACGTCTACAAAGCAAGTGGATTGATGTGACATCTCCACTGACGTAAGGGATGACGCACAATCACACTATCCTTCGCAAGACCCTTCCTCTATATAAGGAAGTTCATTTCATTTGGAGAGGACACGCTCGAGTATAAGAGCTCATTTTTACAACAATTACCAACAACAACAAACAACAAACAACATTACAATTACATTTACAATTATCGATACAATGAAGTATATATAGACAATAAAAAAGTAGTATAATATATTATCAAATTATTATGAACAGTACATCTATGTCCTCATTGGGAGTGAGAAAAGGTTCATGGACTGATGAAGAAGATTTTCTTCTAAGAAAATGTATTGATAAGTATGGTGAAGGAAAATGGCATCTTGTTCCCATAAGAGCTGGTAACTATTAAATTAACTATCACGTTATTTTTATTTGTCTTTCTGTCTCATTTTATTTGACGTTATTACGAATATCATCTGAAAATGTACGTGCAGGTCTGAATAGATGTCGGAAAAGTTGTAGATTGAGGTGGCTGAATTATCTAAGGCCACATATCAAGAGAGGTGACTTTGAACAAGATGAAGTGGATCTCATTTTGAGGCTTCATAAGCTCTTAGGCAACAGGCATGCAAGTTTATGTTTTGACAAAATTTGATTAGTATATATTATATATACGTGTGACTATTTCATCTAAATGTTACGTTATTTTACGTAGATGGTCACTTATTGCTGGTAGACTTCCCGGAAGGACAGCTAACGATGTGAAAAACTATTGGAACACTAATCTTCTAAGGAAGTTAAATACTACTAAAATTGTTCCTCGCGAAAAGATTAACAATAAGTGTGGAGAAATTAGTACTAAGATTGAAATTATAAAACCTCAACGACGCAAGTATTTCTCAAGCACAATGAAGAATGTTACAAACAATAATGTAATTTTGGACGAGGAGGAACA

Blue font: 2x TALE binding site (scTALE ABL1 R); green font: upstream homologous arm; red font: pNOS-NptII-tOCS; orange font: CaMV 35S promoter; purple font: downstream homologous arm.

- LbCpf1 expression cassette:

GAATTCCAATCCCACAAAAATCTGAGCTTAACAGCACAGTTGCTCCTCTCAGAGCAGAATCGGGTATTCAACACCCTCATATCAACTACTACGTTGTGTATAACGGTCCACATGCCGGTATATACGATGACTGGGGTTGTACAAAGGCGGCAACAAACGGCGTTCCCGGAGTTGCACACAAGAAATTTGCCACTATTACAGAGGCAAGAGCAGCAGCTGACGCGTACACAACAAGTCAGCAAACAGACAGGTTGAACTTCATCCCCAAAGGAGAAGCTCAACTCAAGCCCAAGAGCTTTGCTAAGGCCCTAACAAGCCCACCAAAGCAAAAAGCCCACTGGCTCACGCTAGGAACCAAAAGGCCCAGCAGTGATCCAGCCCCAAAAGAGATCTCCTTTGCCCCGGAGATTACAATGGACGATTTCCTCTATCTTTACGATCTAGGAAGGAAGTTCGAAGGTGAAGGTGACGACACTATGTTCACCACTGATAATGAGAAGGTTAGCCTCTTCAATTTCAGAAAGAATGCTGACCCACAGATGGTTAGAGAGGCCTACGCAGCAAGTCTCATCAAGACGATCTACCCGAGTAACAATCTCCAGGAGATCAAATACCTTCCCAAGAAGGTTAAAGATGCAGTCAAAAGATTCAGGACTAATTGCATCAAGAACACAGAGAAAGACATATTTCTCAAGATCAGAAGTACTATTCCAGTATGGACGATTCAAGGCTTGCTTCATAAACCAAGGCAAGTAATAGAGATTGGAGTCTCTAAAAAGGTAGTTCCTACTGAATCTAAGGCCATGCATGGAGTCTAAGATTCAAATCGAGGATCTAACAGAACTCGCCGTCAAGACTGGCGAACAGTTCATACAGAGTCTTTTACGACTCAATGACAAGAAGAAAATCTTCGTCAACATGGTGGAGCACGACACTCTGGTCTACTCCAAAAATGTCAAAGATACAGTCTCAGAAGATCAAAGGGCTATTGAGACTTTTCAACAAAGGATAATTTCGGGAAACCTCCTCGGATTCCATTGCCCAGCTATCTGTCACTTCATCGAAAGGACAGTAGAAAAGGAAGGTGGCTCCTACAAATGCCATCATTGCGATAAAGGAAAGGCTATCATTCAAGATCTCTCTGCCGACAGTGGTCCCAAAGATGGACCCCCACCCACGAGGAGCATCGTGGAAAAAGAAGAGGTTCCAACCACGTCTACAAAGCAAGTGGATTGATGTGACATCTCCACTGACGTAAGGGATGACGCACAATCCCACTATCCTTCGCAAGACCCTTCCTCTATATAAGGAAGTTCATTTCATTTGGAGAGGACACGCTCGAGTATAAGGTAAATTTCTGTGTTCCTTATTCTCTCAAAATCTTCGATTTTGTTTTCGTTCGATCCCAATTTCGTATATGTTCTTTGGTTTAGATTCTGTTAATCTTAGATCGAAGATGATTTTCTGGGTTTGATCGTTAGATATCATCTTAATTCTCGATTAGGGTTTCATAGATATCATCCGATTTGTTCAAATAATTTGAGTTTTGTCGAATAATTACTCTTCGATTTGTGATTTCTATCTAGATCTGGTGTTAGTTTCTAGTTTGTGCGATCGAATTTGTCGATTAATCTGAGTTTTTCTGATTAACAGGAGCTCATTTTTACAACAATTACCAACAACAACAAACAACAAACAACATTACAATTACATTTACAATTATCGATACAATGATGCCCAAGAAGAAGCGCAAGGTGGGACGCGTCTGCAGGATATCAAGCTTGCGGTACCGCGGGCCCGGGATCGCCACCATGAGCAAGCTGGAGAAGTTTACAAACTGCTACTCCCTGTCTAAGACCCTGAGGTTCAAGGTAAAGCCTCGATTTTTGGGTTTAGGTGTCTGCTTATTAGAGTAAAAACACATCCTTTGAAATTGTTTGTGGTCATTTGATTGTGCTCTTGATCCATTGAATTGCTGCAGGCCATCCCTGTGGGCAAGACCCAGGAGAACATCGACAATAAGCGGCTGCTGGTGGAGGACGAGAAGAGAGCCGAGGATTATAAGGGCGTGAAGAAGCTGCTGGATCGCTACTATCTGTCTTTTATCAACGACGTGCTGCACAGCATCAAGCTGAAGAATCTGAACAATTACATCAGCCTGTTCCGGAAGAAAACCAGAACCGAGAAGGAGAATAAGGAGCTGGAGAACCTGGAGATCAATCTGCGGAAGGAGATCGCCAAGGCCTTCAAGGGCAACGAGGGCTACAAGTCCCTGTTTAAGAAGGATATCATCGAGACAATCCTGCCAGAGTTCCTGGACGATAAGGACGAGATCGCCCTGGTGAACAGCTTCAATGGCTTTACCACAGCCTTCACCGGCTTCTTTGATAACAGAGAGAATATGTTTTCCGAGGAGGCCAAGAGCACATCCATCGCCTTCAGGTGTATCAACGAGAATCTGACCCGCTACATCTCTAATATGGACATCTTCGAGAAGGTGGACGCCATCTTTGATAAGCACGAGGTGCAGGAGATCAAGGAGAAGATCCTGAACAGCGACTATGATGTGGAGGATTTCTTTGAGGGCGAGTTCTTTAACTTTGTGCTGACACAGGAGGGCATCGACGTGTATAACGCCATCATCGGCGGCTTCGTGACCGAGAGCGGCGAGAAGATCAAGGGCCTGAACGAGTACATCAACCTGTATAATCAGAAAACCAAGCAGAAGCTGCCTAAGTTTAAGCCACTGTATAAGCAGGTGCTGAGCGATCGGGAGTCTCTGAGCTTCTACGGCGAGGGCTATACATCCGATGAGGAGGTGCTGGAGGTGTTTAGAAACACCCTGAACAAGAACAGCGAGATCTTCAGCTCCATCAAGAAGCTGGAGAAGCTGTTCAAGAATTTTGACGAGTACTCTAGCGCCGGCATCTTTGTGAAGAACGGCCCCGCCATCAGCACAATCTCCAAGGATATCTTCGGCGAGTGGAACGTGATCCGGGACAAGTGGAATGCCGAGTATGACGATATCCACCTGAAGAAGAAGGCCGTGGTGACCGAGAAGTACGAGGACGATCGGAGAAAGTCCTTCAAGAAGATCGGCTCCTTTTCTCTGGAGCAGCTGCAGGAGTACGCCGACGCCGATCTGTCTGTGGTGGAGAAGCTGAAGGAGATCATCATCCAGAAGGTGGATGAGATCTACAAGGTGTATGGCTCCTCTGAGAAGCTGTTCGACGCCGATTTTGTGCTGGAGAAGAGCCTGAAGAAGAACGACGCCGTGGTGGCCATCATGAAGGACCTGCTGGATTCTGTGAAGAGCTTCGAGAATTACATCAAGGCCTTCTTTGGCGAGGGCAAGGAGACAAACAGGGACGAGTCCTTCTATGGCGATTTTGTGCTGGCCTACGACATCCTGCTGAAGGTGGACCACATCTACGATGCCATCCGCAATTATGTGACCCAGAAGCCCTACTCTAAGGATAAGTTCAAGCTGTATTTTCAGAACCCTCAGTTCATGGGCGGCTGGGACAAGGATAAGGAGACAGACTATCGGGCCACCATCCTGAGATACGGCTCCAAGTACTATCTGGCCATCATGGATAAGAAGTACGCCAAGTGCCTGCAGAAGATCGACAAGGACGATGTGAACGGCAATTACGAGAAGATCAACTATAAGCTGCTGCCCGGCCCTAATAAGATGCTGCCAAAGGTGTTCTTTTCTAAGAAGTGGATGGCCTACTATAACCCCAGCGAGGACATCCAGAAGATCTACAAGAATGGCACATTCAAGAAGGGCGATATGTTTAACCTGAATGACTGTCACAAGCTGATCGACTTCTTTAAGGATAGCATCTCCCGGTATCCAAAGTGGTCCAATGCCTACGATTTCAACTTTTCTGAGACAGAGAAGTATAAGGACATCGCCGGCTTTTACAGAGAGGTGGAGGAGCAGGGCTATAAGGTGAGCTTCGAGTCTGCCAGCAAGAAGGAGGTGGATAAGCTGGTGGAGGAGGGCAAGCTGTATATGTTCCAGATCTATAACAAGGACTTTTCCGATAAGTCTCACGGCACACCCAATCTGCACACCATGTACTTCAAGCTGCTGTTTGACGAGAACAATCACGGACAGATCAGGCTGAGCGGAGGAGCAGAGCTGTTCATGAGGCGCGCCTCCCTGAAGAAGGAGGAGCTGGTGGTGCACCCAGCCAACTCCCCTATCGCCAACAAGAATCCAGATAATCCCAAGAAAACCACAACCCTGTCCTACGACGTGTATAAGGATAAGAGGTTTTCTGAGGACCAGTACGAGCTGCACATCCCAATCGCCATCAATAAGTGCCCCAAGAACATCTTCAAGATCAATACAGAGGTGCGCGTGCTGCTGAAGCACGACGATAACCCCTATGTGATCGGCATCGATAGGGGCGAGCGCAATCTGCTGTATATCGTGGTGGTGGACGGCAAGGGCAACATCGTGGAGCAGTATTCCCTGAACGAGATCATCAACAACTTCAACGGCATCAGGATCAAGACAGATTACCACTCTCTGCTGGACAAGAAGGAGAAGGAGAGGTTCGAGGCCCGCCAGAACTGGACCTCCATCGAGAATATCAAGGAGCTGAAGGCCGGCTATATCTCTCAGGTGGTGCACAAGATCTGCGAGCTGGTGGAGAAGTACGATGCCGTGATCGCCCTGGAGGACCTGAACTCTGGCTTTAAGAATAGCCGCGTGAAGGTGGAGAAGCAGGTGTATCAGAAGTTCGAGAAGATGCTGATCGATAAGCTGAACTACATGGTGGACAAGAAGTCTAATCCTTGTGCAACAGGCGGCGCCCTGAAGGGCTATCAGATCACCAATAAGTTCGAGAGCTTTAAGTCCATGTCTACCCAGAACGGCTTCATCTTTTACATCCCTGCCTGGCTGACATCCAAGATCGATCCATCTACCGGCTTTGTGAACCTGCTGAAAACCAAGTATACCAGCATCGCCGATTCCAAGAAGTTCATCAGCTCCTTTGACAGGATCATGTACGTGCCCGAGGAGGATCTGTTCGAGTTTGCCCTGGACTATAAGAACTTCTCTCGCACAGACGCCGATTACATCAAGAAGTGGAAGCTGTACTCCTACGGCAACCGGATCAGAATCTTCCGGAATCCTAAGAAGAACAACGTGTTCGACTGGGAGGAGGTGTGCCTGACCAGCGCCTATAAGGAGCTGTTCAACAAGTACGGCATCAATTATCAGCAGGGCGATATCAGAGCCCTGCTGTGCGAGCAGTCCGACAAGGCCTTCTACTCTAGCTTTATGGCCCTGATGAGCCTGATGCTGCAGATGCGGAACAGCATCACAGGCCGCACCGACGTGGATTTTCTGATCAGCCCTGTGAAGAACTCCGACGGCATCTTCTACGATAGCCGGAACTATGAGGCCCAGGAGAATGCCATCCTGCCAAAGAACGCCGACGCCAATGGCGCCTATAACATCGCCAGAAAGGTGCTGTGGGCCATCGGCCAGTTCAAGAAGGCCGAGGACGAGAAGCTGGATAAGGTGAAGATCGCCATCTCTAACAAGGAGTGGCTGGAGTACGCCCAGACCAGCGTGAAGCACGCCTATCCCTATGACGTGCCCGATTATGCCAGCCTGGGCAGCGGCTCCCCCAAGAAAAAACGCAAGGTGGAAGATCCTAAGAAAAAGCGGAAAGTGGACGGCATTGGTAGTGGGAGCTAAGCTTCTCTAGCTAGAGTCGATCGACAAGCTCGAGTTTCTCCATAATAATGTGTGAGTAGTTCCCAGATAAGGGAATTAGGGTTCCTATAGGGTTTCGCTCATGTGTTGAGCATATAAGAAACCCTTAGTATGTATTTGTATTTGTAAAATACTTCTATCAATAAAATTTCTAATTCCTAAAACCAAAATCCAGTACTAAAATCCAGATC

Purple font: SV40 NLS; blue font: LbCpf1; green font: HA tag; orange font: CaMV 35S promoter; tan font: Trp1 intron; dark blue: AtUBQ1 intron; black font: linkers.

- **Multiple replicon** **with Golden gate level 2 acceptor sites**

Map layout of the multiple replicon system:


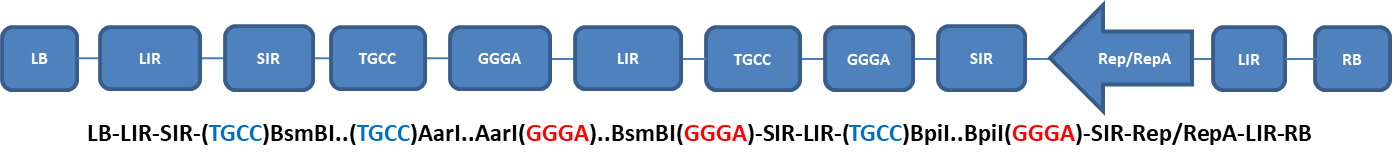


GTTGTTGTGACTCCGAGGGGTTGCCTCAAACTCTATCTTATAACCGGCGTGGAGGCATGGAGGCAGGGGTATTTTGGTCATTTTAATAGATAGTGGAAAATGACGTGGAATTTACTTAAAGACGAAGTCTTTGCGACAAGGGGGGGCCCACGCCGAATTTAATATTACCGGCGTGGCCCCCCCTTATCGCGAGTGCTTTAGCACGAGCGGTCCAGATTTAAAGTAGAAAATTTCCCGCCCACTAGGGTTAAAGGTGTTCACACTATAAAAGCATATACGATGTGATGGTATTTGATGGAGCGTATATTGTATCAGGTATTTCCGTTGGATACGAATTATTCGTACGACCCTCGCTTTGGGTACGTCACGTGGCTCGAGCGCGTAGTCCTCGGTAATATCGCAGAACAAAAGTACCTGATATCGAGTGTACTTCAAGTCAGTGGGAAATCAATAAAATGATTATTTTATGAATATATTTCATTGTGCAAGTAGATAGAAATTACATATGTTACATAACACACGAAATAAACAAAAAAAGACAATCCAAAAACAAACACCCCAAAAAAAATAATCACTTTAGATAAACTCGTATGAGGAGAGGCACGTGATCGCTAGTGCCTGAGACGCCAGTGCCTTAGGCAGGTGCGAGTACGCTCGACACCTGCGGTAGGGATCCACGTCTCAGGGACTTTGGGTATCACGTGGCTCGAGCGCGTAGTCCTCGGTAATATCGCAGAACAAAAGTACCTGATATCGAGTGTACTTCAAGTCAGTGGGAAATCAATAAAATGATTATTTTATGAATATATTTCATTGTGCAAGTAGATAGAAATTACATATGTTACATAACACACGAAATAAACAAAAAAAGACAATCCAAAAACAAACACCCCAAAAAAAATAATCACTTTAGATAAACTCGTATGAGGAGAGGCACGTTGCCGGGTAGCAGAAGGCATGTTGTTGTGACTCCGAGGGGTTGCCTCAAACTCTATCTTATAACCGGCGTGGAGGCATGGAGGCAGGGGTATTTTGGTCATTTTAATAGATAGTGGAAAATGACGTGGAATTTACTTAAAGACGAAGTCTTTGCGACAAGGGGGGGCCCACGCCGAATTTAATATTACCGGCGTGGCCCCCCCTTATCGCGAGTGCTTTAGCACGAGCGGTCCAGATTTAAAGTAGAAAATTTCCCGCCCACTAGGGTTAAAGGTGTTCACACTATAAAAGCATATACGATGTGATGGTATTTGATGGAGCGTATATTGTATCAGGTATTTCCGTTGGATACGAATTATTCGTACGACCCTCGGCGCGTGCCTTGTCTTCGTCGACTAGTCTAGAAGACAAGGGAAGCTTTGGGTACGTCACGTGGCTCGAGCGCGTAGTCCTCGGTAATATCGCAGAACAAAAGTACCTGATATCGAGTGTACTTCAAGTCAGTGGGAAATCAATAAAATGATTATTTTATGAATATATTTCATTGTGCAAGTAGATAGAAATTACATATGTTACATAACACACGAAATAAACAAAAAAAGACAATCCAAAAACAAACACCCCAAAAAAAATAATCACTTTAGATAAACTCGTATGAGGAGAGGCACGTTGCCTCAGTGACTCGACGATTCCCGAGCAAAAAAAGTCTCCCCGTCACACATGTAGTGGGTGACGCAATTATCTTTAAAGTAATCCTTCTGTTGACTTGTCATTGATAACATCCAGTCCTCGTCAGGATTGCAAAGAATTATAGAAGGGATCCCACCTTTTATTTTCTTCTTTTTTCCATATTTAGGGTTGACAGTGAAATCAGACTGGCAACCTATTAATTGCTTCCACAATGGGACGAACTTGAAGGGGATGTCGTCGATGATATTATAGGTGGCGTGTTCATCGTAGTTGGTGAAATCGATGGTACCGTTCCAATAGTTGTGTCGTCCGAGACTTCTAGCCCAGGTGGTCTTTCCGGTACGAGTTGGTCCGCAGATGTAGAGGCTGGGGTGTCGGATTCCATTCCTTCCATTGTCCTTGTTAAATCGGCCATCCATTCAAGGTCAGATTGAGCTTGTTGGTATGAGACAGGATGTATGTAAGTATAAGCGTCTATGCTTACATGGTATAGATGGGTTTCCCTCCAGGAGTGTAGATCTTCGTGGCAGCGAAGATCTGATTCTGTGAAGGGCGACACATACGGTTCAGGTTGTGGAGGGAATAATTTGTTGGCTGAATATTCCAGCCATTGAAGCTTTGTTGCCCATTCATGAGGGAATTCTTCCTTGATCATGTCAAGATATTCCTCCTTAGACGTTGCAGTCTGGATAATAGTTCTCCATCGTGCGTCAGATTTGCGAGGAGAAACCTTATGATCTCGGAAATCTCCTCTGGTTTTAATATCTCCGTCCTTTGATATGTAATCAAGGACTTGTTTAGAGTTTCTAGCTGGCTGGATATTAGGGTGATTTCCTTCAAAATCGAAAAAAGAAGGATCCCTAATACAAGGTTTTTTATCAAGCTGGAGAAGAGCATGATAGTGGGTAGTGCCATCTTGATGAAGCTCAGAAGCAACACCAAGGAAGAAAATAAGAAAAGGTGTGAGTTTCTCCCAGAGAAACTGGAATAAATCATCTCTTTGAGATGAGCACTTGGGATAGGTAAGGAAAACATATTTAGATTGGAGTCTGAAGTTCTTACTAGCAGAAGGCATTTTGGGAGTTGTTGTGACTCCGAGGGGTTGCCTCAAACTCTATCTTATAACCGGCGTGGAGGCATGGAGGCAGGGGTATTTTGGTCATTTTAATAGATAGTGGAAAATGACGTGGAATTTACTTAAAGACGAAGTCTTTGCGACAAGGGGGGGCCCACGCCGAATTTAATATTACCGGCGTGGCCCCCCCTTATCGCGAGTGCTTTAGCACGAGCGGTCCAGATTTAAAGTAGAAAATTTCCCGCCCACTAGGGTTAAAGGTGTTCACACTATAAAAGCATATACGATGTGATGGTATTTGATGGAGCGTATATTGTATCAGGTATTTCCGTTGGATACGAATTATTCGTACGACCCTC

Red font: LIR sequences; purple font: SIR sequences; green font: Rep/RepA; light blue font: multi-cloning site 1 (Level 2 golden gate cloning site with BpiI ,MCS1); orange font: multi-cloning site 2 (Level 2 golden gate cloning site with BsmBI, MCS2); yellow highlighted font: Golden gate level 2 cloning sites (AarI) with TGCC-GGGA flanking sites; black font: spacer sequences.

- **In pHRHKT12.1 for editing SlHKT1;2 (Fig. 4B)**
- The dual KHT12 crRNA expression cassette:

TGATCAAAAGTCCCACATCGATCAGGTGATATATAGCAGCTTAGTTTATATAATGATAGAGTCGACATAGCGATTGTAATTTCTACTAAGTGTAGATACTATTCACCACAGTATCAACTTTAATTTCTACTAAGTGTAGATCCTACAAATGAAAACATGATGATTTTTTT

Red font: AtU6 promoter; green font: LbCpf1 scaffold; purple font: LbCpf1_gHKT12.1; orange font: LbCpf1_gHKT12.2; black G: transcription start; black TTTTTT: termination sequence.

- HKT1;2 donor sequence

GGAGACTCATGAACTTGAAATGCATAGAATGCCTATTTTAACACGTTTTTAACAAATAATAAATAAGTTGTATAGCATCTTATAATTCCACTAACTCTATGATAAGGAAATATCAGTGATCATTTGGCAAGAATTATTATATTCTAACTCCTTTTGGTACGTACCATATGTTGTTGGGATTAATTTAAATCATTGGTAAGAATTATTATATTCTAACTCCTTTTGATACGTACCATATGTTATTGGGATTAATTTAAAGCTTACACAATGTGTCTAGTATAGGCATTCCACTACACAATTTTTACTATAAATTGAACCCTTGAGTATTATAATAAAACTTAAGAGACAAACAAATTTATTTTACTAATGAAGTCATCACTTTCAATTTCTTTCTTTAAGATTATGATGAGTCTTAGGGTAAAGCCATTTTGGATTGAATTAGGGTATTTCACAACCCTTTCATTGTTAGGTTTCTTAGCTTTGAATTATGTTTCAAAACCTAGAACCCTACCGTCTTTTCGTCCTCAAAACCTAGATGTTCTTTTCACTTCTGTTTCTTCAACCACAGTTTCTAGTATGTCCACTATTGAAATGGAAGTTTTCTCAAATGTTCAACTTGTTTTCATGACCATATTGATGTTTCTTGGTGGGGAAGCTTTTACCTCTTTTCTTAGCCTTAAACTCATCAAGAATAAAGAAAGCAAAGATAAATCTTTTAGTAACAAAGATTATGAGCTAGGGAATGTAATAAACGTTGACAATAAGTTAGAAGATGTGATAATAATAAACCCTATCGAAGATCATATTCATGATCATCACGATGAAATTATAAAGATTAAATCGATAAAATTGTTGAGTAATGTGGTTTTTGGATATATTCTTGTTGTTATTCTTCTTGGTTCATCGTTAGTTTCTCTCTATATAATAATCATCCCTAGCGCCAAACAAATCCTTGACCAAAAAGGCCTTAATTTACATACTTTCTCTCTATTCACCACAGTATCAACTTTTGCAGATTGTGGTTTCTTACCAACAAATGAAAACATGATGATTTTCAAGAAAAATTCAGGACTTCTTCTCATTCTTATCCCTCAAGTCCTTCTAGGGAACACTTTGTTTGCTCCTTGTTTACGCATCGTTATAATGTTCTTATGGAAAATCACAAAGAGACATGAGTATGAGTATATTTTGAAGAACTCAAAATGTGTTGGATATTCACATATTTTTCCAAGTTATGAAACAATTGGTATTGCTATTACTGTTGTGGGATTAATAGTATTTCAATTTGTTATGTTTTGTTCATTGGAGTGGAATTCTGAAGGTACTTCTGGATTAAGTACTTATGAGAAGATTGTGGGATCTTTGTTTGAAGTTGTGAATACAAGGCATGCTGGTCTATCTGTATTTGATCTTTCAACTTTTACTCCATCAATCTTAGTATTGTTTGCCTTAATGATGTAAGTATTCTCTACAATCTATGTTCTTTCGTGTTCTGTCTCAATTTACGTGACATATATACTACTTCTTTGTTTTATATCTGTGTCTCTGGAAGAATTAATGTCAACTTTCTATAGTTAGAAATAACTTAATTTTCAAATTCCCTTTTTATCTTTGATTCACATGAATATCTAAGATTTGCTTTATATTGCAAATTTTCAAATTTGATGTTAAGTTATTAAAAAGTATCACATGAACAAGAGAGAGGAGAAAGTAGTTCATTGTTTAGAAATTTATCATCCACATGCAACTATGTTAATACTAACATATTTGTTTAAGTTGTTGTAATAGGATATGATAAACTTTTAACAACAAATTAAGTTTAACTATTAAACAAATGTGTTAAAAGTGTTA

Purple font: LbCpf1_gHKT12.1; orange font: LbCpf1_gHKT12.2; yellow highlighted font: modified PAM and LbCpf1 gRNA core region to prevent recut after HDR editing; red font: N217D (AAT to GAT).

- **LbCpf1 expression cassette**: Same as used in pHR01
- **Plant selection marker**: pNOS-NptII-tOCS cloned from pICSL11024 (pICH47732::NOSp-NPTII-OCST) (Addgene Plasmid #51144).

Primers used for analyzing SlHKT1;2 events

| **No.** | **Product** | **Primer name** | **Sequence (5’-3’)** | **Product length (bp)** |
| --- | --- | --- | --- | --- |
| 1 | Targeted HKT12 allele | UPHKT12-F1 | TTCACATGCTTTGACCCATAAA | 2034 |
| 2 |  | DNHKT12-R1 | CTCTTCCTATAAACGTGCACTCA |  |
| 3 | Sequencing | HKT12-sF1 | CAAAGATTATGAGCTAGGGAATGT |  |
| 4 | HKT12 Replicon | GR-F1 | TTGAGATGAGCACTTGGGATAG | 483 |
| 5 |  | pNOS-cR1 | AACGTGACTCCCTTAATTCTCC |  |
| 6 | T-DNA | RB-qF2 | CTCTTAGGTTTACCCGCCAATA | 2236 |
| 7 |  | LbCpf1-cR1 | CGAGAAGCTGGATAAGGTGAA |  |
| 8 | GAPDH (Solyc05g014470.2) | GAPDH-F1 | CCATAACCTAATTTCTCTCTC | 1073 |
| 9 |  | GAPDH-R1 | GTCATGAGACCCTCAACAAT |  |

- **pANT1^ox^**
- **SlANT1 overexpression cassette**

TCAACATGGTGGAGCACGACACTCTGGTCTACTCCAAAAATGTCAAAGATACAGTCTCAGAAGATCAAAGGGCTATTGAGACTTTTCAACAAAGGATAATTTCGGGAAACCTCCTCGGATTCCATTGCCCAGCTATCTGTCACTTCATCGAAAGGACAGTAGAAAAGGAAGGTGGCTCCTACAAATGCCATCATTGCGATAAAGGAAAGGCTATCATTCAAGATCTCTCTGCCGACAGTGGTCCCAAAGATGGACCCCCACCCACGAGGAGCATCGTGGAAAAAGAAGAGGTTCCAACCACGTCTACAAAGCAAGTGGATTGATGTGACATCTCCACTGACGTAAGGGATGACGCACAATCCCACTATCCTTCGCAAGACCCTTCCTCTATATAAGGAAGTTCATTTCATTTGGAGAGGACACGCTCGAGTATAAGAGCTCATTTTTACAACAATTACCAACAACAACAAACAACAAACAACATTACAATTACATTTACAATTATCGATACAATGATTATGAACAGTACATCTATGTCATCATTGGGAGTGAGAAAAGGTTCATGGACTGATGAAGAAGATTTTCTTCTAAGAAAATGTATTGATAAGTATGGTGAAGGAAAATGGCATCTTGTTCCCATAAGAGCTGGTAACTATTAAATTAACTATCACGTTATTTTTATTTGTCTTTCTGTCTCATTTTATTTGACGTTATTACGAATATCATCTGAAAATGTACGTGCAGGTCTGAATAGATGTCGGAAAAGTTGTAGATTGAGGTGGCTGAATTATCTAAGGCCACATATCAAGAGAGGTGACTTTGAACAAGATGAAGTGGATCTCATTTTGAGGCTTCATAAGCTCTTAGGCAACAGGCATGCAAGTTTATGTTTTGACAAAATTTGATTAGTATATATTATATATACGTGTGACTATTTCATCTAAATGTTACGTTATTTTACGTAGATGGTCACTTATTGCTGGTAGACTTCCCGGAAGGACAGCTAACGATGTGAAAAACTATTGGAACACTAATCTTCTAAGGAAGTTAAATACTACTAAAATTGTTCCTCGCGAAAAGATTAACAATAAGTGTGGAGAAATTAGTACTAAGATTGAAATTATAAAACCTCAACGACGCAAGTATTTCTCAAGCACAATGAAGAATGTTACAAACAATAATGTAATTTTGGACGAGGAGGAACATTGCAAGGAAATAATAAGTGAGAAACAAACTCCAGATGCATCGATGGACAACGTAGATCCATGGTGGATAAATTTACTGGAAAATTGCAATGACGATATTGAAGAAGATGAAGAGGTTGTAATTAATTATGAAAAAACACTAACAAGTTTGTTACATGAAGAAATATCACCACCATTAAATATTGGTGAAGGTAACTCCATGCAACAAGGACAAATAAGTCATGAAAATTGGGGTGAATTTTCTCTTAATTTACCACCCATGCAACAAGGAGTACAAAATGATGATTTTTCTGCTGAAATTGACTTATGGAATCTACTTGATTAAGCTTGTCAAGCAGATCGTTCAAACATTTGGCAATAAAGTTTCTTAAGATTGAATCCTGTTGCCGGTCTTGCGATGATTATCATATAATTTCTGTTGAATTACGTTAAGCATGTAATAATTAACATGTAATGCATGACGTTATTTATGAGATGGGTTTTTATGATTAGAGTCCCGCAATTATACATTTAATACGCGATAGAAAACAAAATATAGCGCGCAAACTAGGATAAATTATCGCGCGCGGTGTCATCTATGTTACTAGATCGA

Green font: CaMV 35S short promoter; purple font: SlANT1 CDS; red font: NOS terminator;

- **Plant selection marker**: pNOS-NptII-tOCS cloned from pICSL11024 (pICH47732::NOSp-NPTII-OCST) (Addgene Plasmid #51144).
- **SlRAD51 expression cassette of MR03**

GAATTCCAATCCCACAAAAATCTGAGCTTAACAGCACAGTTGCTCCTCTCAGAGCAGAATCGGGTATTCAACACCCTCATATCAACTACTACGTTGTGTATAACGGTCCACATGCCGGTATATACGATGACTGGGGTTGTACAAAGGCGGCAACAAACGGCGTTCCCGGAGTTGCACACAAGAAATTTGCCACTATTACAGAGGCAAGAGCAGCAGCTGACGCGTACACAACAAGTCAGCAAACAGACAGGTTGAACTTCATCCCCAAAGGAGAAGCTCAACTCAAGCCCAAGAGCTTTGCTAAGGCCCTAACAAGCCCACCAAAGCAAAAAGCCCACTGGCTCACGCTAGGAACCAAAAGGCCCAGCAGTGATCCAGCCCCAAAAGAGATCTCCTTTGCCCCGGAGATTACAATGGACGATTTCCTCTATCTTTACGATCTAGGAAGGAAGTTCGAAGGTGAAGGTGACGACACTATGTTCACCACTGATAATGAGAAGGTTAGCCTCTTCAATTTCAGAAAGAATGCTGACCCACAGATGGTTAGAGAGGCCTACGCAGCAAGTCTCATCAAGACGATCTACCCGAGTAACAATCTCCAGGAGATCAAATACCTTCCCAAGAAGGTTAAAGATGCAGTCAAAAGATTCAGGACTAATTGCATCAAGAACACAGAGAAAGACATATTTCTCAAGATCAGAAGTACTATTCCAGTATGGACGATTCAAGGCTTGCTTCATAAACCAAGGCAAGTAATAGAGATTGGAGTCTCTAAAAAGGTAGTTCCTACTGAATCTAAGGCCATGCATGGAGTCTAAGATTCAAATCGAGGATCTAACAGAACTCGCCGTCAAGACTGGCGAACAGTTCATACAGAGTCTTTTACGACTCAATGACAAGAAGAAAATCTTCGTCAACATGGTGGAGCACGACACTCTGGTCTACTCCAAAAATGTCAAAGATACAGTCTCAGAAGATCAAAGGGCTATTGAGACTTTTCAACAAAGGATAATTTCGGGAAACCTCCTCGGATTCCATTGCCCAGCTATCTGTCACTTCATCGAAAGGACAGTAGAAAAGGAAGGTGGCTCCTACAAATGCCATCATTGCGATAAAGGAAAGGCTATCATTCAAGATCTCTCTGCCGACAGTGGTCCCAAAGATGGACCCCCACCCACGAGGAGCATCGTGGAAAAAGAAGAGGTTCCAACCACGTCTACAAAGCAAGTGGATTGATGTGACATCTCCACTGACGTAAGGGATGACGCACAATCCCACTATCCTTCGCAAGACCCTTCCTCTATATAAGGAAGTTCATTTCATTTGGAGAGGACACGCTCGAGTATAAGGTAAATTTCTGTGTTCCTTATTCTCTCAAAATCTTCGATTTTGTTTTCGTTCGATCCCAATTTCGTATATGTTCTTTGGTTTAGATTCTGTTAATCTTAGATCGAAGATGATTTTCTGGGTTTGATCGTTAGATATCATCTTAATTCTCGATTAGGGTTTCATAGATATCATCCGATTTGTTCAAATAATTTGAGTTTTGTCGAATAATTACTCTTCGATTTGTGATTTCTATCTAGATCTGGTGTTAGTTTCTAGTTTGTGCGATCGAATTTGTCGATTAATCTGAGTTTTTCTGATTAACAGGAGCTCATTTTTACAACAATTACCAACAACAACAAACAACAAACAACATTACAATTACATTTACAATTATCGATACAATGGAGCAGCAGCACAGGAATCAGAAGTCGATGCAAGACCAAAATGATGAAATCGAGGATGTTCAACACGGCCCTTTTCCAGTTGAACAACTTCAGGTTTCCCCCCTTTTCTTCTCTTAATTGAGGAGTTAATTCAATCCTTTTTATATATCAAGTGTCCTAAATCTTGGATTGGGGTAGTTGTTTTGGTGATAGTGATTGTTTATGATTTCAGATTCAGTGTAGCTGGGTCTGATCTTGAATCTTGAATTGTACTTCGTAGGTTAAATGTTGAATCTTGAAATGTGGTTCTGGGTTGAATCTTGAATGGTAAAAAACTGAATTTTGTGAAACAGCTTTTTTTCAAGGTTGAATCTCATCTGCCTCGCTTATGGGAAATGCTTTTTTTTGTTTTGGGGTTTTCTATACTGTCCCCTTTTTGGCTTCTTGGAATGGGTTCGCTATCATAGTGCTCTTGTGGATTATTTCAAGCTAATATTTTTACAGTTATATCTATCTGTCCTTTACTGCCTTAGTTTTGAAGCTAAATGTTGTAGAATGCTTTGTGAAATGGGTTCTCGAAGCGATACCACTGATCTTGAAAACCTGGATGAGTGTTTTTTTGGTATGATGTTTCGATGGAGTCATGGAGCCCTGCGACAAGCCTAAGTTTAGTATTTAAGTGGAGAATGGTAGAGGAGTGACCCATCATTCCTGAGTTCGAATGCTATTGTTGGCAATAAGGGTTGGCTCAAAACAAATTTCTAGGTCATCAATTAAAAAAAACGATGTTGCTACAATGGTTTTGCTGCCCGTAATTACAACCAGTTTTCATTAGAAATTTGTAGGTTACTATCAGAGTGTACAGAATTGAGGGCACCATGAAAAGCTGCTTCTAGAAAGGCACTTGACACAAAATGATTTACATCATGACGCATGATATGGAGGGTCCAATAGGTGGGCGTTGATGGTCAGATATTTTAGTGTGCATCTGGGTTATGCGGTTGGCTTATCAGCTTTGCTCCTTACAAGATTTTATCAGTTTAGATCTTTTTTTGAAGAATTTTAAGTCTCGAGTGATTAACATTTACAGTAACTCAGATAAGTACTTATCATAAGTTTCTCATACTGCAGATTTTAAAAAGATTTACACGAAGTGAAACCTAAACTCTTGAGGATTTTTTAATCTTACTGGTAGTACATAAAAGCTGGACTAGGCATTTCCTAGATTTTTTTCTTGGAATTGTTAATATTTAAGAGGCAATATCTTTGAGTATCTTTTTCCAGTCTAGCTTGTAGTATAGTTGTTTCTGTTGAATGACACCCTGTAATTCTTCCAACTTTTAAAGGAAATGGTCCATTAGTTACTGAGGCATATAGCTTTTTTGTCCTCAAGGCATCAGGGATTGCAGCTCTAGATGTAAAAAAACTCAAGGATGCTGGTCTATGTACAGTTGAATCTGTTGTTTATGCTCCAAGAAAGGAACTTCTGCAGATAAAAGGAATTAGTGAAGCTAAAGTTGACAAGATTATTGAGGCAGGCATGTCTCTCTTCATTTAACTGCCATTGCCAGTGCTGATGTTTTTTCCCCTTACCTTCTTTTTTCACCCATTTCTGCAATTTTTTATCAGCAATCGGTACTAAATCTTTTTAATGCTGCATATGAACAGCTTCAAAATTAGTGCCTTTGGGATTCACTAGTGCCAGCCAACTCCATGCACAGAGGCTTGAAATCATACAGATAACTTCTGGATCGAAAGAACTTGACAAGATATTAGAAGGTAGTTATTTATCTAATTTGTGAACTTCTAGAACCGGAATTTGACCTGGCATCACAACCTCATACTTTTTTTGTCACTTGTCAGGAGGAATCGAAACTGGATCTATTACTGAAATTTACGGAGAGTTCCGATGTGGAAAGACTCAGCTGTGTCACACACTATGCGTGACTTGTCAAGTACGATAACATAGACATTTTTTTAATTTTAAAATCCCATAATAAAGTGCACAAGATTCATTCAAATATCATTCTTTATCTGTTATTTCAGCTTCCATTAGATCAGGGAGGTGGTGAAGGGAAAGCAATGTACATTGATGCTGAGGGTACTTTCAGACCACAAAGACTTTTACAAATTGCAGACAGGTATGGATTGAATGGTCCTGATGTCCTGGAGAATGTAGCCTATGCTCGAGCTTATAATACCGATCATCAATCAAGACTTTTGCTTGAGGCAGCCTCAATGATGGTGGAGACTAGGTTTGCTCTCATGATTGTGGACAGTGCTACTGCCCTTTATAGAACTGACTTCTCTGGGAGAGGAGAGTTGTCTGCCAGGCAGATGCATCTTGCAAAGTTTCTGAGAAGCCTTCAGAAGTTAGCAGATGAGGTATTTAGCATGTCCCTCATTTGTGCATATTATCCAAAATGGAGTCTCCTCCTTCCTATTTTTCTCTCTTTGGTGGTGGTTAATGTTTTAGAATTAGATTAGGGGTTCTTGACTTACTTTTTCTATCTATCCTTTACTTTTACACTTGCAGTTTGGTGTTGCTGTTGTTATTACGAACCAAGTTGTTGCTCAAGTGGATGGTTCTGCTGTATTTGCTGGGCCTCAAATAAAACCCATTGGTGGCAACATCATGGCACATGCTTCTACGACGAGGTACGCATAATAGTACAACTATAACTTGCAGGCGCAGCTTATCTTGATAGCTGATATTTTACTCTCTTCAAATTCTGACAGACTAGCTCTGAGGAAGGGTAGGGCCGAGGAGCGGATTTGTAAAGTAGTCAGTTCGCCATGCTTAGCTGAAGCAGAAGCAAGATTTCAAATTTCTGTTGAAGGAGTCACTGATGTAAAGGACTAAGCTTCTCTAGCTAGAGTCGATCGACAAGCTCGAGTTTCTCCATAATAATGTGTGAGTAGTTCCCAGATAAGGGAATTAGGGTTCCTATAGGGTTTCGCTCATGTGTTGAGCATATAAGAAACCCTTAGTATGTATTTGTATTTGTAAAATACTTCTATCAATAAAATTTCTAATTCCTAAAACCAAAATCCAGTACTAAAATCCAGAT

Orange font: CaMV 35S promoter; dark blue: AtUBQ1 intron; purple font: SlRAD51 exons; red font: CaMV 35S terminator.

- **SlRAD54 expression cassette of MR04**

GAATTCCAATCCCACAAAAATCTGAGCTTAACAGCACAGTTGCTCCTCTCAGAGCAGAATCGGGTATTCAACACCCTCATATCAACTACTACGTTGTGTATAACGGTCCACATGCCGGTATATACGATGACTGGGGTTGTACAAAGGCGGCAACAAACGGCGTTCCCGGAGTTGCACACAAGAAATTTGCCACTATTACAGAGGCAAGAGCAGCAGCTGACGCGTACACAACAAGTCAGCAAACAGACAGGTTGAACTTCATCCCCAAAGGAGAAGCTCAACTCAAGCCCAAGAGCTTTGCTAAGGCCCTAACAAGCCCACCAAAGCAAAAAGCCCACTGGCTCACGCTAGGAACCAAAAGGCCCAGCAGTGATCCAGCCCCAAAAGAGATCTCCTTTGCCCCGGAGATTACAATGGACGATTTCCTCTATCTTTACGATCTAGGAAGGAAGTTCGAAGGTGAAGGTGACGACACTATGTTCACCACTGATAATGAGAAGGTTAGCCTCTTCAATTTCAGAAAGAATGCTGACCCACAGATGGTTAGAGAGGCCTACGCAGCAAGTCTCATCAAGACGATCTACCCGAGTAACAATCTCCAGGAGATCAAATACCTTCCCAAGAAGGTTAAAGATGCAGTCAAAAGATTCAGGACTAATTGCATCAAGAACACAGAGAAAGACATATTTCTCAAGATCAGAAGTACTATTCCAGTATGGACGATTCAAGGCTTGCTTCATAAACCAAGGCAAGTAATAGAGATTGGAGTCTCTAAAAAGGTAGTTCCTACTGAATCTAAGGCCATGCATGGAGTCTAAGATTCAAATCGAGGATCTAACAGAACTCGCCGTCAAGACTGGCGAACAGTTCATACAGAGTCTTTTACGACTCAATGACAAGAAGAAAATCTTCGTCAACATGGTGGAGCACGACACTCTGGTCTACTCCAAAAATGTCAAAGATACAGTCTCAGAAGATCAAAGGGCTATTGAGACTTTTCAACAAAGGATAATTTCGGGAAACCTCCTCGGATTCCATTGCCCAGCTATCTGTCACTTCATCGAAAGGACAGTAGAAAAGGAAGGTGGCTCCTACAAATGCCATCATTGCGATAAAGGAAAGGCTATCATTCAAGATCTCTCTGCCGACAGTGGTCCCAAAGATGGACCCCCACCCACGAGGAGCATCGTGGAAAAAGAAGAGGTTCCAACCACGTCTACAAAGCAAGTGGATTGATGTGACATCTCCACTGACGTAAGGGATGACGCACAATCCCACTATCCTTCGCAAGACCCTTCCTCTATATAAGGAAGTTCATTTCATTTGGAGAGGACACGCTCGAGTATAAGGTAAATTTCTGTGTTCCTTATTCTCTCAAAATCTTCGATTTTGTTTTCGTTCGATCCCAATTTCGTATATGTTCTTTGGTTTAGATTCTGTTAATCTTAGATCGAAGATGATTTTCTGGGTTTGATCGTTAGATATCATCTTAATTCTCGATTAGGGTTTCATAGATATCATCCGATTTGTTCAAATAATTTGAGTTTTGTCGAATAATTACTCTTCGATTTGTGATTTCTATCTAGATCTGGTGTTAGTTTCTAGTTTGTGCGATCGAATTTGTCGATTAATCTGAGTTTTTCTGATTAACAGGAGCTCATTTTTACAACAATTACCAACAACAACAAACAACAAACAACATTACAATTACATTTACAATTATCGATACAAATGGGGGCCGAAGGAGAGGCTCTTTCAGCATCGAGCGACGAATCCCTTAGAGTGCCAGAGAAGGACATCGATTGCGTATCTAATAGTGGCGACGACGACGACGATGACGATGAATGGAATGTAGCATCTCAGACGGGTACCAGTTCGCCAGATGAAGATCGCAAATCGCAAAATGTTGATGCTCTTGTGAGGGGTAACCTTATTGTGAAAAGACAGTCACTACTTCCACGAGTCTATTCAGTGACAGATGCAGCAGCCAATCTCCGAAAGCCATTCAAACCTCCAAGCTCCAATGGTTACAGTAGTAGCAATGAGCATCTAGCTCGCCGTCTTTGTGCTCGTAAAAGGTTTGTGCCATGGGGTTCAACAAGTCCAACATTAATTGCTATTACAAACAGGCTGAAAGCTCCAGAAGCTGCTGAGATAGATGTGGTGGAGGACAATTTAGAACTGCCACCTGGTGTTGAACCTCTGGTGTTGTGGCAGCCTGAAGAAATTGTGGAGGAAGGCTATAGTTTAGTTCCTATAATTGTGGATCTGTTGCTTGTTAGGTTCCTTCGTCCTCATCAAAGGTACTTCATATGAGATAGGCAGTATAGGGTGTGTTTGATTATATTTATGTGATATTTAAAGGCGTATTGTTTAACCAAGTGCATGAATGAAAAAGCAATGTTAGAATTGGAAGCTATGGATATTTAAAATGTATCATAGGTGAAGCACTCAAAAGAATTCTTTATATCTTCTCATTAAGTTACTTTGTGCCATTTTATTTCATGACATTCAAGTTGAATTTGCAACATTAGTTTCTGAAGATATACATCAGTAAGTTGATAACCTGTTCACTCCTCCATCATAACTCCAAAAAGAACATTTCTTCCTTATTTCATTAAAAGAAAGACTCCAAAAAAGAACATTTCTATTATTCGCCAAACTTCAACCTCTTAGTTTTATCTTTGATGCCATATGTGTTTTAATCTGTTTTTAAATGAATTTCAATCAAGTTAAGCAACATTGAGAAACTCTCAAATTTGGCTATGTTTGCAGCATGCTGCAGCATCTATATCCTCTTAAAAAACTCTTCATGATTTTACTTGCAGGGAAGGGGTCCAGTTCATGTTTGACTGTGTGTCAGGTGCACTTAGTAGCTTCAACATTAATGGATGCATTTTAGCTGATGATATGGGGTAAATTATTTTTTGTTATTCTCCCTGACTATGTGTTTCTAAGTTGAGTCATCTTAGGTGTTTCATTTTGGCTTATTCACTGCTCTTTGCGTTTTAGTGCAGTTTGGGGAAAACTCTGCAATCAATTACACTACTTTACACTCTTCTTCGCCAGGGATTTGATGGAAAACCTATGGTCAGGAAGGCAATAATTGCGACCCCTACTAGTCTTGTTAGTAATTGGGAGGCCGAAATCAATAAATGGGTTGGAGAAAGAGTTAAACTTGTTGCTTTATGTGAAAGCACCAGAGATGATGTTGTTTCTGGCATAGAAAGTTTTATCAATCCCCATAGTAATATACAGGTATGTTTTCTGAGAGATTCTAGTTACATTATGATTGAGGGGTATCAATTCATATATTGTTCTCCCAGGTTTTAATTGTTTCCTATGAGACATTTCGGATGCATTCTTCAAAATTCAGTAATTCTGGATCATGCGACCTTCTCATATGTGATGAGGCTCACAGATTGAAAAATGATCAAACACTGACTAATCGAGTAAGACATGTTTACTTATTTTAGATGACTTAAATTACTTTTTGTGGATGAACTCTGTAATCTTTGTCGAGCCTAATTTAAAACTTGCAATTAGGCATTGGCTTCTCTAGCATGCAAACGCCGGGTTCTGCTATCAGGGACCCCAATGCAAGTGAGATCCTATCTGACTTTTCTTTAGGCTTCTCTAGCTAGAGTCGATCGACAAGCTCGAGTTTCTCCATAATAATGTGTGAGTAGTTCCCAGATAAGGGAATTAGGGTTCCTATAGGGTTTCGCTCATGTGTTGAGCATATAAGAAACCCTTAGTATGTATTTGTATTTGTAAAATACTTCTATCAATAAAATTTCTAATTCCTAAAACCAAAATCCAGTACTAAAATCCAGAT

Orange font: CaMV 35S promoter; dark blue: AtUBQ1 intron; purple font: SlRAD54 exons; red font: CaMV 35S terminator.
