# Supplementary material for: Highly efficient homology‐directed repair using CRISPR/Cpf1‐geminiviral replicon in tomato
Source: Plant Biotechnol J. 2020 Apr 1;18(10):2133–43. doi: 10.1111/pbi.13373 (PMC7540044; doi:10.1111/pbi.13373)
Supplement: Supplementary file 4 — Data S2 Analysis of guide RNA activity. [file PBI-18-2133-s001.docx]

- **Assessment of guide RNA activity via indel mutation traces of PCR products flanking targeted sites decomposed by ICE Synthego software**

**Map of targeted site:**


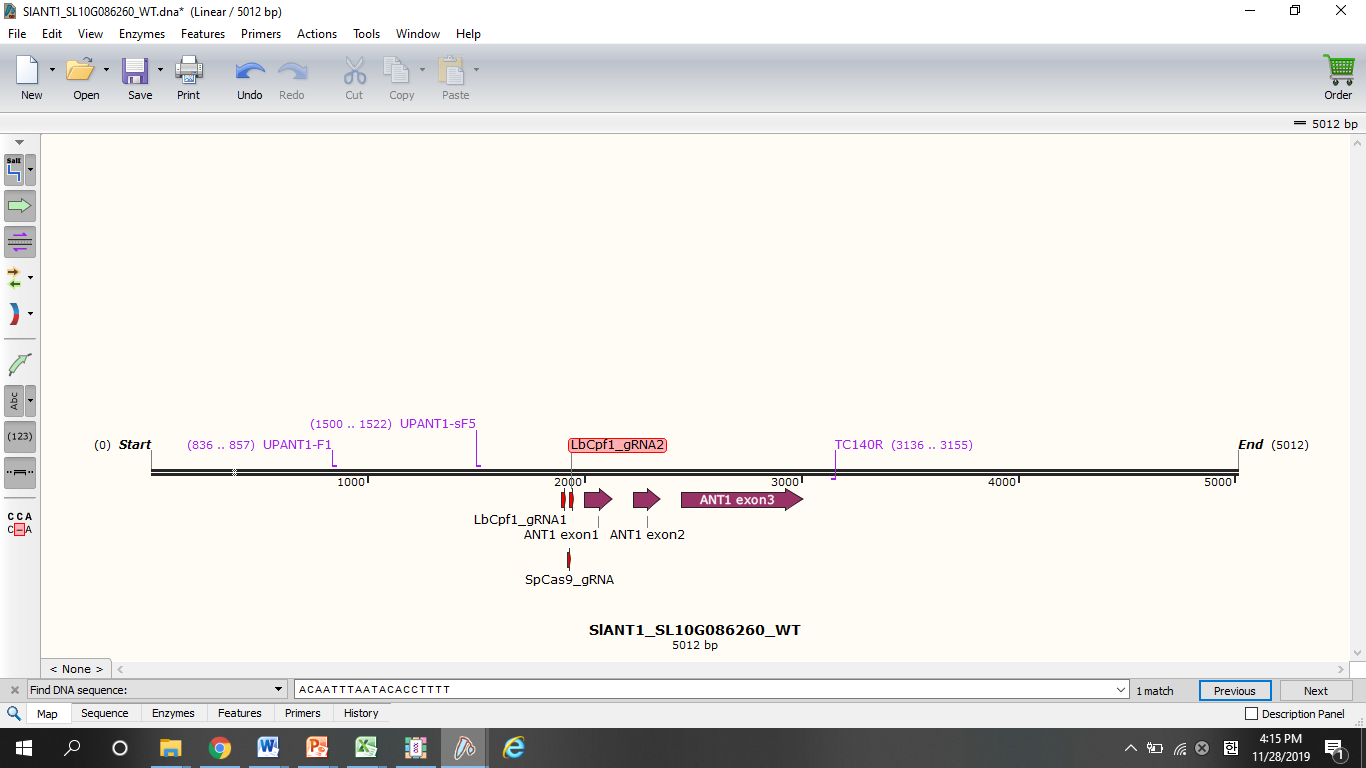


- Sequences of the guide RNAs:

| **Name** | **Sequence (5’-3’)** | **Expression vector** |
| --- | --- | --- |
| LbCpf1_gRNA1 | TAGAAGGCTCTCTACAAGTTGGT | pHR01 |
| LbCpf1_gRNA2 | ATACACCTTTTAGGCACGTGTAT |  |
| SpCas9_gRNA | ACAATTTAATACACCTTTT | pTC217 |

- **Method used for assessment of activity of the guide RNAs**
- **Decomposition of Sanger sequencing data using ICE Synthego software**

Method: PCR products amplified using the primers (see table below) flanking the targeted sites of SlANT1 in pHR01 (ID11 to ID121) and pTC217 (ID91 to ID920)-transformed events and WT were purified on 0.8% agarose gel and subjected to Sanger sequencing and the sequencing data files (.ab1 extension) were decomposed using ICE Synthego (Hsiau et al., 2019). The WT sequencing ab1 file was used as the reference sequence for assessment of any DNA modification at the flanking site of that of the ANT1 events (Figure A and B). The results are summarized in Table A and B.

- **Primers used for amplifying flanking region of targeted sites**

| **Name** | **Sequence (5’-3’)** | **Product size (bp)** | **Targeted locus** |
| --- | --- | --- | --- |
| UPANT1-F1 | TGCGATGATCTACGGTAACAAA | 2320 | SlANT1 (Solyc10g086260.1) |
| TC140R | TACCACCGGTCCATTCCCTA |  |  |
| UPANT1-sF5 | CCCTCTCACGATTAATGATAGTT | Sequencing |  |


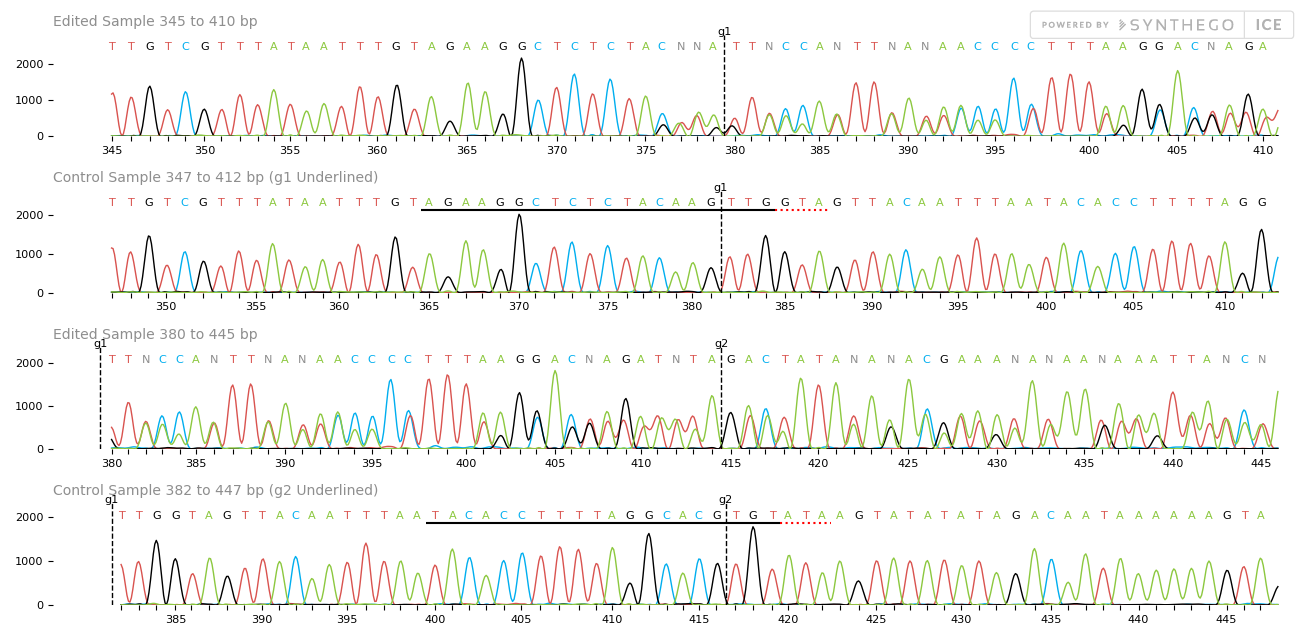


Fig. A. A representative diagram showing ICE Synthego sequence comparison of ID11 event (the first row for LbCpf1_gRNA1 and the third row for LbCpf1_gRNA2) and control sequence (the second and fourth rows). The vertical discontinuous lines denote hypothetical cutting site at 18bp downstream of the PAM sites (red boxes)


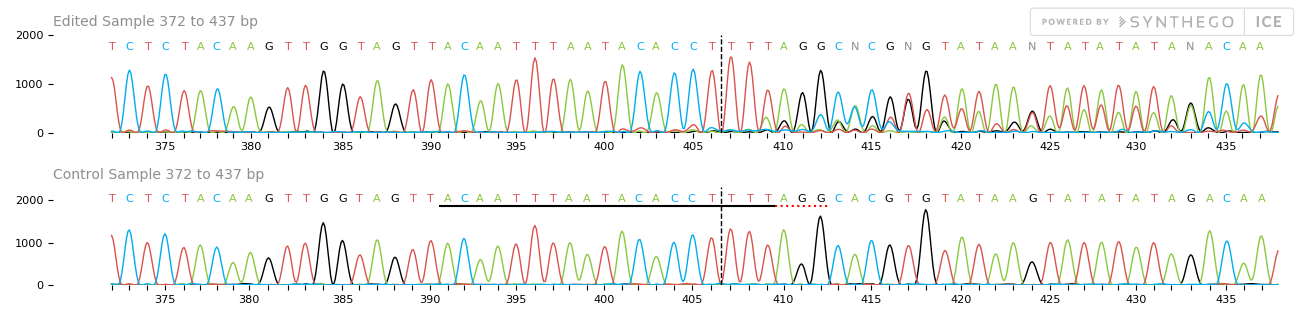


Fig. B. A representative diagram showing ICE Synthego sequence comparison of ID91 event (upper row) and control sequence. The vertical discontinuous lines denote hypothetical cutting site at 3 bp upstream of the PAM sites (red boxes).

Table A: Indel mutation rates among SpCas9-based samples decomposed by ICE Synthego software

| **No.** | **Transformed event** | **Indel mutation rate (%)** | **Knock-out rate (%)** |
| --- | --- | --- | --- |
| 1 | ID91 | 33 | 33 |
| 2 | ID92 | 20 | 20 |
| 3 | ID93 | 0 | 0 |
| 4 | ID94 | 0 | 0 |
| 5 | ID95 | 0 | 0 |
| 6 | ID96 | 0 | 0 |
| 7 | ID97 | 22 | 22 |
| 8 | ID98 | 8 | 8 |
| 9 | ID99 | 26 | 26 |
| 10 | ID910 | 0 | 0 |
| 11 | ID911 | 40 | 40 |
| 12 | ID912 | 0 | 0 |
| 13 | ID913 | 25 | 25 |
| 14 | ID914 | 20 | 20 |
| 15 | ID915 | 59 | 59 |
| 16 | ID916 | 0 | 0 |
| 17 | ID917 | 43 | 24 |
| 18 | ID918 | 26 | 25 |
| 19 | ID919 | 30 | 30 |
| 20 | ID920 | 0 | 0 |
| Average rate /adjusted rate | | 60/17.6 | 60/16.6 |

^Average rate = sum of events containing mofidication/total number of events; Adjusted rate= sum of all rates/total number of events^

Table B: Indel mutation rates at SlANT1 locus recorded from pHR01 transformed events reported by ICE Synthego

| **No.** | **Transformed event** | **Indel mutation rate (%)** | **Knock-out rate (%)** |
| --- | --- | --- | --- |
| 1 | ID11 | 92 | 90 |
| 2 | ID13 | 0 | 0 |
| 3 | ID14 | 95 | 26 |
| 4 | ID15 | 97 | 97 |
| 5 | ID16 | 97 | 97 |
| 6 | ID17 | 0 | 0 |
| 7 | ID18 | 0 | 0 |
| 8 | ID19 | 0 | 0 |
| 9 | ID110 | 100 | 0 |
| 10 | ID111 | 97 | 97 |
| 11 | ID112 | 0 | 0 |
| 12 | ID114 | 100 | 0 |
| 13 | ID115 | 96 | 96 |
| 14 | ID116 | 97 | 0 |
| 15 | ID117 | 90 | 2 |
| 16 | ID118 | 90 | 1 |
| 17 | ID119 | 85 | 79 |
| 18 | ID120 | 42 | 42 |
| 19 | ID121 | 0 | 0 |
| 20 | ID122 | 0 | 0 |
| 21 | ID123 | 0 | 0 |
| Average rate /adjusted rate | | 61.9/56.1 | 38.1/29.9 |

^Average rate = sum of events containing mofidication/total number of events; Adjusted rate= sum of all rates/total number of events^

**Reference**

Hsiau T, Conant D, Rossi N, Maures T, Waite K, Yang J, Joshi S, Kelso R, Holden K, Enzmann BL, Stoner R (2019) bioRxiv 251082; doi: https://doi.org/10.1101/251082.
