# Supplementary material for: Highly efficient homology‐directed repair using CRISPR/Cpf1‐geminiviral replicon in tomato
Source: Plant Biotechnol J. 2020 Apr 1;18(10):2133–43. doi: 10.1111/pbi.13373 (PMC7540044; doi:10.1111/pbi.13373)
Supplement: Supplementary file 5 — Data S3 Potential off‐targets of LbCpf1_gRNA1. [file PBI-18-2133-s002.docx]

- **Off-target analysis for LbCpf1_gRNA1 and LbCpf1_gRNA2 using Cas-OFFinder (Bae et al., 2014)**

Critetia for searching for off-target: 5’-TTTV-3’ PAM; maximum 4 mismatches; Tomato genome database: *Solanum lycopersicum* (SL2.4)

**For LbCpf1_gRNA1:**

| **Target** | **Chromosome** | **Position** | **Direction** | **Mismatches** | **Note** |
| --- | --- | --- | --- | --- | --- |
| crRNA: TTTVTAGAAGGCTCTCTACAAGTTGGT   DNA: TTTGgAGAAGatTCTCTACcAGTTGGT | SL2.40ch03 | 46387517 | + | 4 | Potential off-target #1 |
| crRNA: TTTVTAGAAGGCTCTCTACAAGTTGGT   DNA: TTTGTtGtAGGtTCTCTACAAtTTGGT | SL2.40ch04 | 2294364 | - | 4 | Potential off-target #2 |
| crRNA: TTTVTAGAAGGCTCTCTACAAGTTGGT   DNA: TTTGTAGAAGGCTCTCTACAAGTTGGT | SL2.40ch10 | 64463280 | - | 0 | On-target |

Red font: mismatches between LbCpf1_gRNA1 and the off-target site

**For LbCpf1_gRNA2:**

| Target | Chromosome | Position | Direction | Mismatches | Note |
| --- | --- | --- | --- | --- | --- |
| crRNA: TTTVATACACCTTTTAGGCACGTGTAT   DNA: TTTAATACACCTTTTAGGCACGTGTAT | SL2.40ch10 | 64463245 | - | 0 | On-target |

- **Potential off-target #1 of LbCpf1_gRNA1: Tomato build SL2.40-SL2.40ch03-46386519..46388519**

CGTACTTTTCATCACGTCAATGTATTCTAAAACTTTAACGAGTTACAAATTTTGGCGTTCTTCAAATAAATTCCAACTTACCATCTATATATTTTTTAAAATCCCTTTTTGTTGCATTTAGTAAAGTTGTTTCCTGTTTACTACTCAGCAACCTTTCCTTGAAAACTAAAATCAGATGAACTCTTTTATTTCTTTACTTTCCGTTTTTGATTTTTTTTATATTAAGTTACTTGCCCTTATGACTTAAGTTAGACTAAATTAATGCGCTCAAGAATTGTTAGCTACTTCCATTAAGAAGATATGAAGCATGGAAATAAAAATAGATAGAACTTTTCACAACTTAATTATATGAAATAATTTAAGGGTTTGGCTGAGTGTGTGTGTGGCTTTTAGTTTAAGAGGGTCTTTAGGTCCTTTACATGATTTTCTTCCCTTCGATTAATCAAGTAGAATATTACTTATGGTAAATGTACTTTTTCCAAACCCTTTTTAAAAAGAAGAATTCCGTTACATTTTGATACATTACAACCGATTCTGAAAAGGCTTTCACTTTTCAATACTCAGATATCAGTTTTTATAGAATAATATAAAATAGGGGCAAATTGTTCTTAAGAATTAAACTGTGCAAACCTTAAGCTTCTTTGTAGGGCTTTGTTCTTTGAGTACTGCTTTAACACTCATTTATCTTTCAGAGGTGAAGAAGGCCGTATGCTACTGGAGGAGTGCTTGTTGATAACTGAGAAGTTCAGGGGTGAAGGGCATCCCAGTTCAGTCTCTCATCTTGTGAATCTAGCCACTTCTTATTCACAGTCCAAACTTTTTGCAGAAGCTGAGCGCTTGCTAAGGATGAGTTTGCAGATTATGTATCAGAATGTTTCACCTGATGATCAATCCATTACTTTCCCAATGCTACATCTTGCAGTTACTCTCTACAATCTAAACCGTAATGAGGAGGCCGAAAAACACGCATTAGAGGTTCTGCGAATCCGGGAGAGGGCATTTGGAGAAGATTCTCTACCAGTTGGTGAGCTTGTCCTTTTCCCTATTGCTTTATGCTAAATGCTAAGCAGTATCTTTTGATCTGTTAGTAAAAAATGTTTGCCATCAATATGTTTGCAAGCTGTTTCACCATTCATGTACTATTTGCTTACCCTCTTTATCTTTATCAAACAAAAAAAGAAGTAACTGGTTTGGTGTTTTCAGGTTCTTTTCTTTCATCTCTCCCCTTTTGGCTAAAGAGTTTATGTATGCAGGGGAGGCTCTTGACTGTTTGGTCTCCATTCAGACCCAGCTGGGGAAGGATGATGGTGAATTGCTGGAGTTGCTCAAGAGAGTACTGAAAATTCAAGAGAAAGCTTTCGGTACTGACAGTGAGGAAGTCATGGAAACACTTAAAAAAGTTGTATATTACTTGGAGAAGATGGGGAGGAAACATGAGGGACTCCCTCTGGAAAGAAGATTATCAAAGCTCCGAACGAAATTTAAGCAAATGGTTCAATATTAAAGATCCTACAAGGCCGCATGTTTGACATGTAATTATTTTCATGCTATTAACAGTTCAGATCATTGTGGTTGCAAGATAAAAATGTCCTTCTACAGGGAGTTCGCATGCCACAAAAGGAAAAGAGACACTGGACTATAGCACAAGACCGGGTTTCTTCCTGCCATGGTGGACAAGGAAGACAGCCAAAACTAACAAATTTTCAGGCATATGAAAGAAATTCTTTTTAGCTATATCTATCCAAGAGTGTAGTGATATTTCAGAGAACATTCAGAACCAGATATGAAAAGCTTAGGGGACAGTTACCGTCAAATGAATACTACCAAAGGTATCATATAGGTTCTTGATGCATTGTCTGAAAGTGTTAAAAGAGCCTTGCTTACCATCACTAATCCTGCTACTGCTTCTCTTCTTATAGGTGTGGAATTGCATTGGACATGTCGAACCAAATGGGACGGTGTCCACATTCCAACTACTATCTGATTGACTACAATGTACAA

Yellow highlighted font: potential off-target binding site; Red font: mismatches between LbCpf1_gRNA1 and the off-target site; Purple font: LbCpf1_PAM; Blue font: primer binding site for PCR amplification; orange font: sequencing primer binding site.

- **Potential off-target #2 of LbCpf1_gRNA1: Tomato build SL2.40-SL2.40ch03-46386519..46388519**

CAGTTACCAGTTCAAATAAAAAGGCCCCTGGAAAGCTACATGGAAAAGCGTGGCACCAACAAAGGTGAAATATTTTTCATGGTTTGTAGTGAGAAAAGCATGTCTAACTCATGAAGCACTTAAGAAGAGGAAATCCAAATAGCATCTAGATGCATCTAAGCATGAAGGCAACTGAAACTAACACTCACATATTCCTTCACTGAAAGGTAACATCCCAAATTTGGGCACTCTTCATTTCTGTGACTAGGGAGAATTGGACAATGCTAGAACACACTGCAAACTTACTAAGCCGCTGGATCAGAAGGGGAGTGAGCAAAAGACAACAAAGGTAGTGGAAAACTGTACCAGCATGCATATGGTGGAATACCATAGTTCTGTAAATCGTTCTCTTGAGCTACATGGGCATAGCAACTTTGCGCTAAGTGGAACCACAATCATTATCATCAAACACCAATCTTCTGTTGTTTTAGTTACACCAATCTTATACACGATTAAGTTCTTGTATAATCACAAACTTGTTAACTAACCAAATAGATACTTCTGAGATGTTCATTAATTTTGTTTCACTTAAATGCCGTCGAAGTGGAGACACCTAAGTAGAAAACTTGGCCTTGCAATTTTTGAACAAGCCATTAGGAGCTTTCGCTGGGATCGCAAAACGAAATTGTTCGGTACCTTCTGTAGTTGGATGTGCTAAGCTTGTCTGATTATGTCGCTCCATTTTATATCAATGTTTATTCTGATATAGTTCTTTGAAAAAGTAAGGCAAGTTATTCCTTTTGCAGGTAGAACATGTGCTGGTGGATCGTGTGTGCCAGTTTTTGTCTGATGATTTGCGGGATGTAAAGGTGATCAAATTTGGCATTATTTCAATATTGTTGTGGAAATCTAGATTAAGCTCTCTCTATGTTTGCGTTAATAGAATTAGGTAGCTGTTTTATTTTTTTTGATAAGGCAAGCAATTTTATAAACAATGGAATACCCCAATATACAAGTTGAATACCAAATTGTAGAGAACCTACAACAAAACATGGCTACAGAACACCAATCCACTGTACTTGGTGCCAGCCTAACTTGGCTGTAATTCTGTCAAATGGGTGCCTTTTAGTGGATTTTGACATACAAGGGTTGGTACTTTAATGTCATGTGTCTGATGACTCGTCGCTAATTTTGCAGGAATCCCGTCGCAGGTTTGATAAAGCTGCATCCACTTATGACCAGGTAATGGAGATGTTTCAATAGAAACAAGTTTAAAGCTGCTTTTTTAAATGCTAAGCAGACTTTATGTGCAAAATTCTGTTCATTTGGAAGATAACACTTCCAGTTCTTTTTGTTATTAATTTCACTCCTGAGTTGCTAAACTGCCAGGCACGTGAAAGGTTTTCTTCTCTGAAGAAGAATGCACGGGATGAAGTTGTTACTGAACTTGAAGAAGTAAGCCCTTTCTCTGAAAAAGGTTATCTTTGTGGATTGTTCATCCTCTTATGTGTATCTCTCTATCTGCCTAACTTGATGAGGTGTAACCTAACACAAACTGATAAATGTTGCAAAAATTACCAACCAAAAGTGATTGGTATCCTGATTGTTATGTGTTCAAATAAAATTATGTCCTAAAGAATCTCAAAATTTTGGTCTGCTTTAGGTCTTTTCTTATAGGATCCCTCTGAGGAATCATTTACATGCTTGTTTTCCTTGAGGAAAAAAAATGAGGTGAAAGGTGTTGCTTTGGGAGGAACTTGTAAGAGAATTCATCCTCATGAACATATCACAGATAAATACCTTTGTCCAACCACATCTTCCATAGTAGTACTATTTTTTTATTTTATCGAGATTGTGTAGGGACAGGATGAGAAACTCATTCTGGCATGCTATACGTGAAGAGCATCATCCATATTGCTACCAATTATTTTATAGGTAGGATATTATATTAGCGAAAGTATAAATAACTCAAGCTCATGCTCTACTTAAAAAAAAAAAAGAAGAAACTCAGCCTCATGCTTT

Yellow highlighted font: potential off-target binding site; Red font: mismatches between LbCpf1_gRNA1 and the off-target site; Green font: LbCpf1_PAM binding site; Blue font: primer binding site for PCR amplification; orange font: sequencing primer binding site.

- **Primers used for potential off-target analysis in this study**

| **Name** | **Sequence (5’-3’)** | **Product size (bp)** | **LbCpf1_gRNA1 potential off-target** |
| --- | --- | --- | --- |
| ANT1po1-F1 | ACTACTCAGCAACCTTTCCTTG | 1382 | Potential off-target #1 |
| ANT1po1-R1 | GCGGCCTTGTAGGATCTTTAAT |  |  |
| ANT1po1-sF1 | CATCCCAGTTCAGTCTCTCATC | Sequencing |  |
| ANT1po2-F1 | CTCTTGAGCTACATGGGCATAG | 1469 | Potential off-target #2 |
| ANT1po2-R1 | GTTTCTCATCCTGTCCCTACAC |  |  |
| ANT1po2-sF1 | TGTCTGATTATGTCGCTCCATTT | Sequencing |  |

- **Decomposition of Sanger sequencing data using ICE Synthego software**

Method: PCR products (only single band at the expected size was observed) amplified using the primers with the ANT1 HDR events #C11; C12; C13; C141; C142; C18; C19; C110; C111; C112; C113; C114; C115; C116; C117 and WT were purified on 0.8% agarose gel and subjected to Sanger sequencing and the sequencing data files (.ab1 extension) were decomposed using ICE Synthego (Hsiau et al., 2019). The WT sequencing ab1 file was used as the reference sequence for assessment of any DNA modification at the flanking site of that of the ANT1 HDR events.

No off-targeted modification was found at both the potential off-target sites in all the tested samples:


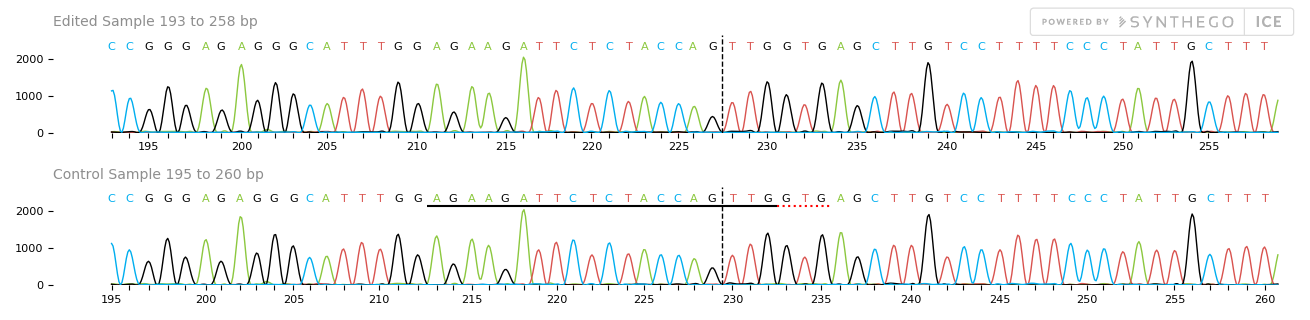


Fig. A. A representative diagram showing ICE Synthego sequence comparison of C11 event (upper row) and control sequence at the potential off-target site #1 of LbCpf1_gRNA1. The vertical discontinuous lines denote hypothetical cutting site at 18bp downstream of the sequence containing PAM sites (red boxes).


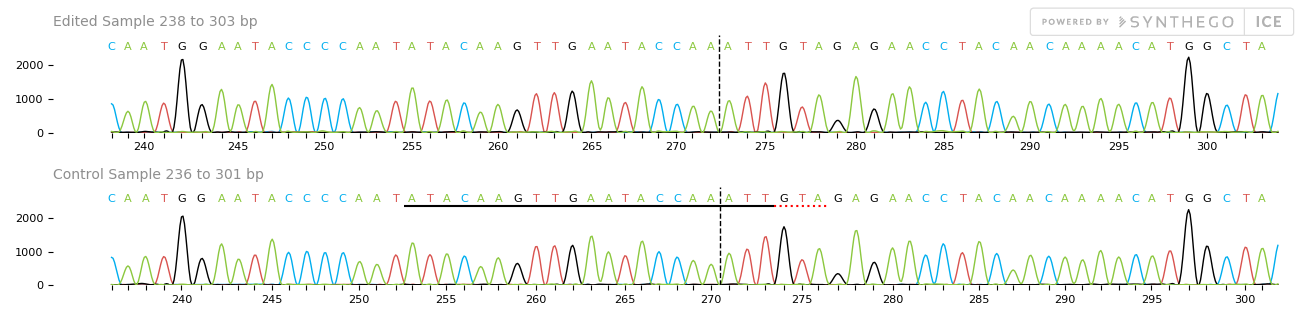


Fig. B. A representative diagram showing ICE Synthego sequence comparison of C11 event (upper row) and control sequence at the potential off-target site #1 of LbCpf1_gRNA1. The vertical discontinuous lines denote hypothetical cutting site at 18bp downstream of the sequence containing PAM sites (red boxes)

Table A: Knock-out scores of the potential off-target site #1 reported by ICE Synthego

| **Sample** | **Indel mutation rate** | **KO-Score** |
| --- | --- | --- |
| C11-po1 | 0 | 0 |
| C12-po1 | 0 | 0 |
| C13-po1 | 0 | 0 |
| C141-po1 | 0 | 0 |
| C142-po1 | 0 | 0 |
| C18-po1 | 0 | 0 |
| C19-po1 | 0 | 0 |
| C110-po1 | 0 | 0 |
| C111-po1 | 0 | 0 |
| C112-po1 | 0 | 0 |
| C113-po1 | 0 | 0 |
| C114-po1 | 0 | 0 |
| C115-po1 | 0 | 0 |
| C116-po1 | 0 | 0 |
| C117-po1 | 0 | 0 |

Table B: Knock-out scores of the potential off-target site #2 reported by ICE Synthego

| **Sample** | **Indel mutation rate** | **KO-Score** |
| --- | --- | --- |
| C11-po2 | 0 | 0 |
| C12-po2 | 0 | 0 |
| C13-po2 | 0 | 0 |
| C141-po2 | 0 | 0 |
| C142-po2 | 0 | 0 |
| C18-po2 | 0 | 0 |
| C19-po2 | 0 | 0 |
| C110-po2 | 0 | 0 |
| C111-po2 | 0 | 0 |
| C112-po2 | 0 | 0 |
| C113-po2 | 0 | 0 |
| C114-po2 | 0 | 0 |
| C115-po2 | 0 | 0 |
| C116-po2 | 0 | 0 |
| C117-po2 | 0 | 0 |

**Reference**

Bae S., Park J., & Kim J.-S. Cas-OFFinder: A fast and versatile algorithm that searches for potential off-target sites of Cas9 RNA-guided endonucleases. Bioinformatics 30, 1473-1475 (2014).

Hsiau T, Conant D, Rossi N, Maures T, Waite K, Yang J, Joshi S, Kelso R, Holden K, Enzmann BL, Stoner R (2019) bioRxiv 251082; doi: https://doi.org/10.1101/251082.
