# Supplementary material for: Highly efficient homology‐directed repair using CRISPR/Cpf1‐geminiviral replicon in tomato
Source: Plant Biotechnol J. 2020 Apr 1;18(10):2133–43. doi: 10.1111/pbi.13373 (PMC7540044; doi:10.1111/pbi.13373)
Supplement: Supplementary file 6 — Data S4 qRT‐PCR analyses of SlRAD51 and SlRAD54 mRNA levels. [file PBI-18-2133-s003.docx]

**Analysis of RNA transcript levels of RAD51 and RAD54 expressed in pMR03 or pMR04 transformed events by qRT-PCRs**

- **Linearized maps of SlRAD51 and SlRAD54 loci and oligos used for qRT-PCRs.**

**
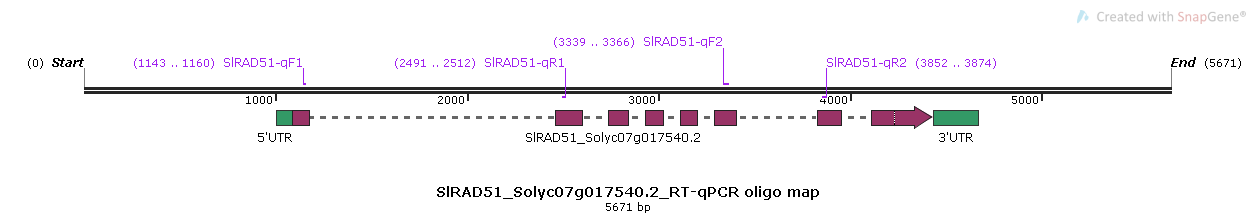
**

**
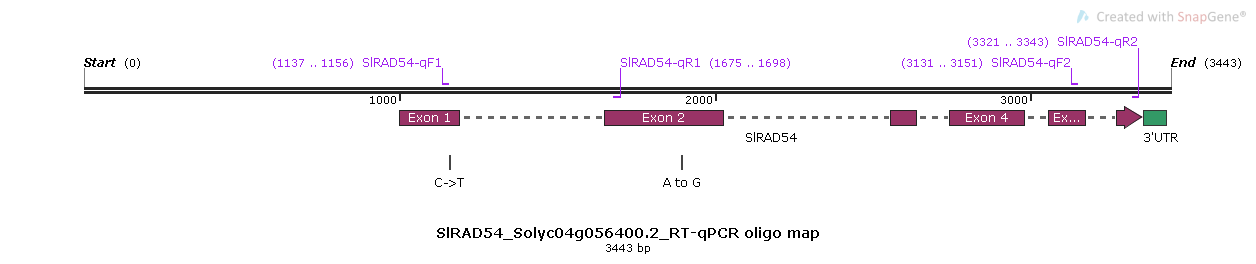
**

- We designed and evaluated two pairs of primers for each of the locus that helped amplifying exon region.
- **Primers used in this analysis**

| **No.** | **Primer name** | **Sequence (5’-3’)** | **Gene name (locus name)** | **Binding sites** | **Product (bp)** |
| --- | --- | --- | --- | --- | --- |
| 1 | SlRAD51-qF1 | ATGTTCAACACGGCCCTT | SlRAD51 (Solyc07g017540.2) | Exon 1-exon 2 | 91 |
| 2 | SlRAD51-qR1 | CATAGACCAGCATCCTTGAGTT |  |  |  |
| 3 | SlRAD51-qF2 | GAGCTTATAATACCGATCATCAATCAAG |  | Exon 6-Exon 7 | 114 |
| 4 | SlRAD51-qR2 | CAGTTCTATAAAGGGCAGTAGCA |  |  |  |
| 5 | SlRAD54-qF1 | GTACCAGTTCGCCAGATGAA | SlRAD54 (Solyc04g056400.2) | Exon 1-exon 2 | 105 |
| 6 | SlRAD54-qR1 | CTGAATAGACTCGTGGAAGTAGTG |  |  |  |
| 7 | SlRAD54-qF2 | TGTGATGAGGCTCACAGATTG |  | Exon 5-Exon 6 | 119 |
| 8 | SlRAD54-qR2 | AGATAGGATCTCACTTGCATTGG |  |  |  |
| 9 | SlPDS-qF1 | GAGTCCAAGGTAGTTCAGCTTAT | SlPDS (Solyc03g123760.2) | Exon 1-exon 1 | 83 |
| 10 | SlPDS-qR1 | CCTTTGCAAGCAACCATCTC |  |  |  |
| 11 | SlPDS-qF2 | GGTAGCGAATCAATGGGTCATA |  | Exon 1-exon 2 | 122 |
| 12 | SlPDS-qR2 | TCCAGCTCTGGTCTTGGATA |  |  |  |
| 13 | GR-F1 | TTGAGATGAGCACTTGGGATAG | Circularized Replicon | | 501 |
| 14 | pCf.ANT1-R4 | ACCTCAACGACGCAAGTATT |  |  |  |
| 15 | RB-qF2 | CTCTTAGGTTTACCCGCCAATA | T-DNA | | 557 |
| 16 | GR-F1 | TTGAGATGAGCACTTGGGATAG |  |  |  |
| 17 | GAPDH-F1 | CCATAACCTAATTTCTCTCTC | GAPDH (Solyc05g014470.2) | | 1073 |
| 18 | GAPDH-R1 | GTCATGAGACCCTCAACAAT |  |  |  |

- **qRT-PCR procedure and analysis of data**

All of the qPCR and qRT-PCR analyses were performed following the MIQE guideline (Bustin et al., 2009). Briefly total RNAs were isolated from plant tissues using RNeasy mini Qiagen kits (cat. no. 74104, Qiagen, USA) and subjected to reverse transcription for synthesizing 1^st^ cDNA strands using QuantiTect Reverse Transcription Kit and protocol (cat. no. 205311, Qiagen, USA). At least two pairs of primers were tested for each target gene/cDNA for evaluating their efficiencies and primers pairs with ~ 100% efficiency were ultimately used for qPCR/qRT-PCR. The similar assessment was also applied for the internal genes for normalizing the amplicon levels. qPCR/qRT-PCRs were performed using intercalating dyes (KAPA SYBR FAST Universal, cat. No. KK4601, Sigma, USA) for detecting products. Thermocycling was conducted with Illumina Eco Real-Time PCR System (Illumina, USA). Analyses of amplicon levels were performed using delta delta Cq method (Livak and Schmittgen, 2001) with internal gene/transcript of SlPDS and were plotted using Excel software (Microsoft, USA).

- **SlRAD51 or SlRAD54 transcript levels expressed in transgenic pMR03 or pMR04 events, respectively. pMR02 samples were used as references.**

Table A: Relative transcript levels of SlRAD51 measured in the transformed events of MR03

| **Sample Name** | **Relative RNA levels normalized to SlPDS** | **Fold change** | **SD** | **Note** |  |
| --- | --- | --- | --- | --- | --- |
| MR02 event 1 | 0.52 | 1.00 | 0.90 | transgenic |  |
| MR02 event 2 | 1.07 | 2.06 | 0.90 | transgenic |  |
| MR03 event 1 | 270.94 | 522.18 | 0.58 | transgenic |  |
| MR03 event 2 | 24.21 | 46.66 | 0.90 | transgenic |  |
| MR03 event 3 | 2.11 | 1.26 | 0.91 | Non-transgenic | |

Table B: Relative transcript levels of SlRAD54 measured in the transformed events of MR04

| **Sample Name** | **Relative RNA levels normalized to SlPDS** | **Fold change** | **SD** | **Note** |
| --- | --- | --- | --- | --- |
| MR02 event 1 | 0.03 | 1.00 | 1.10 | transgenic |
| MR02 event 2 | 0.07 | 2.77 | 0.84 | transgenic |
| MR04 event 1 | 0.02 | 0.79 | 1.01 | Non-transgenic |
| MR04 event 2 | 0.49 | 19.35 | 1.00 | transgenic |
| MR04 event 3 | 2.11 | 83.68 | 1.00 | transgenic |

**Reference**

Bustin, S.A., Benes, V., Garson, J.A., Hellemans, J., Huggett, J., Kubista, M., Mueller, R., Nolan, T., Pfaffl, M.W., Shipley, G.L., Vandesompele, J., Wittwer, C.T. 2009. The MIQE guidelines: minimum information for publication of quantitative real-time PCR experiments. Clin Chem 55(4):611-622. doi: 10.1373/clinchem.2008.112797.

Livak, K.J., Schmittgen, T.D. 2001. Analysis of relative gene expression data using real-time quantitative PCR and the 2(-Delta Delta C(T)) Method. Methods. 25(4):402-408.
