# Supplementary material for: Highly efficient homology‐directed repair using CRISPR/Cpf1‐geminiviral replicon in tomato
Source: Plant Biotechnol J. 2020 Apr 1;18(10):2133–43. doi: 10.1111/pbi.13373 (PMC7540044; doi:10.1111/pbi.13373)
Supplement: Supplementary file 7 — Data S5 Southern blot analysis of GE1 plants. [file PBI-18-2133-s006.docx]

**Southern Blot analysis for ANT1 HDR GE1 plants**

The Southern blot technique was implemented using MSU Potato Lab's protocol (https:/msu.edu/course/css/451/LabProtocols/09SOUTHERNprocedit.pdf) with minor modification. Essentially, genomic DNAs (gDNAs) were isolated from tomato leaves using DNeasy Plant Maxi Kit (cat. no. 68163, Qiagen, Gemany). Approximately 800 µl of gDNA was eluted from column and subjected to precipitation by adding 2.5 volume of absolute ethanol and 1/30 volume of sodium acetate 3M, incubating at -80oC for 2h, centrifuging at 13rpm for 20 minutes and drying for 30 minutes in a 37oC incubator to completely remove residual ethanol. The final eluted volume was 60 µl of AE buffer with ~500-1000 ng/µl.

Twenty micrograms of the gDNAs was digested overnight (~18 h) at 37oC with NsiI (NEB, USA) and inactivated at 65oC for 20 minutes. The digested product was precipitated by adding 2.5 volume of absolute ethanol and 1/30 volume of sodium acetate 3M, incubating at -80oC for 2h, centrifuging at 13rpm for 20 minutes and drying for 30 minutes in a 37oC incubator to completely remove residual ethanol. The dried DNA was suspended in 30 ul of EB buffer and 6 ul of DNA loading dye 6x was added for agarose gel running. The digested product was loaded in a 0.8% agarose gel and resolved by running overnight (~18h) at low voltage (30V). Resolved DNA bands were overnight transferred onto a Hybond N+ membrane (GE Healthcare, USA) by capillary transferring system. The blotted membrane was treated two times with high energy UV (1200x100 µJ/cm2) for crosslinking the DNAs to the membrane.

The membrane was then pre-hybridized and overnight hybridized with DIG-labeled Probe specific for the downstream homologous arm (Supplemental Figure 2) in a plastic bag. The probe was amplified by PCR using primers flanking the downstream homologous arm (see primer table below). Probes were DIG-labeled by random priming using a Random Primed DNA Labeling Kit (cat.no. 11004760001, Roche, Switzerland) following the manufacture’s protocol. The probed membrane was washed stringently and bound DIG probe was detected using DIG Nucleic Acid Detection Kit (cat.no. 11175041910, Roche, Switzerland) following the manufacture’s protocol. Below are details of each part conducted in the analysis.

**Primers used for making Probe**

| **Name** | **Sequence (5’-3’)** | **Template** | **Product size (bp)** |
| --- | --- | --- | --- |
| DNANT1-pF1 | TCTATGTCCTCATTGGGAGTG | pHR01 | 675 |
| DNANT1-pR1 | TGTTCCTCCTCGTCCAAAattaca |  |  |

- **Setting up digestion reactions**

| **Component** | **1x (ul)** |
| --- | --- |
| 10x buffer 3.1 | 15 |
| NsiI (10U/ul) | 6 |
| gDNA (20ug) | 53.1 |
| H2O | 61 |
| Sum | 150 |

- Incubated the reaction tubes in a water-bath at 37^o^C for 18h.
- Inactivated the enzymes at 80^o^C for 20 minutes.
- Precipitated the digested gDNA with 1/30 volumes of NaOAc 3M and 2.5 volumes (250ul) of ethanol 100% (molecular biology grade) and incubated at -80^o^C for 4 hour.
- Centrifuged the tubes at 13rpm for 20’, 4^o^C and discarded supernatant.
- Dried DNA pellets at 37^o^C for 2h and add 30ul EB.
- Added 6ul of 6x loading dye for loading.
- **Agarose gel running**
- Gel size: 14.3cmx21.1cm; need to prepare ~250ml TAE 1x, 0.8% agarose
- Run at 30V overnight (18h)
- **Capillary Transferring of DNAs**
- Depurination in 0.25N HCl: 1 time, 20’; agitation 30rpm.
- Rinsed briefly 2 times with H2O.
- Denaturation in SOLUTION D: 2 times, 15’ each; agitation 30rpm.
- Rinsed briefly 2 times with H2O.
- Neutralization in SOLUTION N: 2 times, 15’ each; agitation 30rpm.
- Pre-wetted Whatman paper wick with 20x SSC and put in a plastic tank. Removed air bubbles using plastic pipette.
- Pre-wetted 3 sheets of gel-sized Whatman paper and placed on the wick.
- Pre-wetted gel in 20x SSC and placed upside down on the whatman paper.
- Pre-wetted gel-sized Hybond N+ membrane in 20x SSC and placed on the gel.
- Pre-wetted 3 sheets of gel-sized Whatman paper and placed on the membrane.
- Placed paper towels (~15cm) on the Whatman paper.
- Placed the gel casting tray on the top of the paper towel.
- Placed ~800 g Schott bottle on the top
- **Pre-hybridization**
- Put the membrane in a clean plastic bag. Use gloved hands and blunt ended forceps.
- Warm 40mls of prehybridization solution to 42^o^C.
- Boil 1000ul of Salmon Testes DNA (10mg/ml) for 10min and then place on ice for 2 min. Add the 1000ul of DNA to the 40mls of prehybridization solution. The final concentration of DNA is 250ng/ml. This is used to block nonspecific binding sites on the blot.
- Add the prehybridization solution to the blot in the bag. Seal and place the bag in hybridization oven that has been warmed to 42^o^C.
- Prehybridize blot for at least 3h30 at 42^o^C. Longer times are possible.
- **Hybridization**
- Prepare hybridization solution for hybridization as follows. Hybridize a 20x30cm blot with 40mls of the buffer. The amount varies depending on the amount of stock probe that you have. Warm the Hybridization solution to 42^o^C.
- Reused the hypridization buffer of the first blot by warming up at 65^o^C for 20’
- Add 6 ul of the DIG-labelled Probe to the warm hybridization solution and mix by shaking well.
- Remove prehybridization solution from hybridization tube. (The prehybridization solution can be reused 2 times. Store at -20^o^C. To reuse, the Salmon DNA in the solution must be denatured. Place the solution in a 65^o^C water bath for 15min. The flashpoint of pure formamide is 68^o^C therefore do not boil the solution.)
- Do not let blot dry in the bag. Immediately add the hybridization solution and place in hybridization oven. Make sure the tube is balanced! Incubate overnight at 37^o^C.
- **DETECTION**
- After hybridization and stringency washes, rinse membrane briefly with WASHING BUFFER about 2-5 min in a plastic tray with the DNA side up.
- Incubate membrane in 100mls of BUFFER 2 for 30 min.
- Dilute anti-DIG-AP conjugate 150 mU/ml (1:5,000 dilution) =10ul in 50ml of fresh BUFFER 2.
- Incubate membrane for 30 min in the antibody solution on shaker either in a plastic tray. Ensure that the solution is covering the entire blot with gentle agitation (30rpm).
- In a plastic tray wash the membrane 2X 15 min. with 100ml of WASHING BUFFER. Apply gentle agitation (30rpm).
- Equilibrate membrane 2-5 min. in 20 ml BUFFER 3.
- Add 400 μl of NBT/BCIP stock solution (vial 4) to 20 ml of Detection buffer. Note: Store protected from light!
- Add the solution to the membrane for color development and keep in dark up to 16h.
- Taking photograph of the blot.
- **Southern blot buffers**

**A. Agrose gel treatment**

1) 0.25N

2) Solution D, Denature solution: 1.5M NaCl and 0.5M NaOH

3) Solution N, Neutralizing solution: 0.5M Tris, 1.5M NaCl and 1mM EDTA; **pH to 7.5**

4) 20 X SSC: 3.0M NaCl, 0.3M NaCitrate ; **pH to 7.0**

**B. Hybridization and washing**

1. Prehybridization and Hybridization solution:

5X SSC, 2% Block solution, 0.1% N-lauroylsarcosine, 0.2% SDS

0.5 volumes of Pure Formamide (deionized)

Aliquot in 50ml tubes and store at -20^o^C until use.

Salmon Testes DNA and Probes are added just before use.

2. 2X Wash Solution: 2X SSC, 0.1% SDS

3) 0.5X Wash Solution: 0.5X SSC, 0.1% SDS

**C. Detection**

**Solutions:**

**1) Maleic Acid Buffer**

0.1M Maleic acid , 0.15M NaCl, pH to 7.5 using NaOH pellets**.**

**2) WASHING BUFFER**

Maleic Acid Buffer, Make the same as above

0.3% Tween 20

**3) Blocking stock solution 10x conc**.

Blocking reagent from BMB, 10% (w/v) in maleic acid buffer. Dissolve blocking reagent by constantly stirring on a heating block at (65^o^C). Do not boil the solution. It is difficult to get into solution and may take several hours. Be sure that all of it has dissolved and then autoclave. Store at 4^o^C. The solution is opaque.

**4) BUFFER 2** (make fresh for each use)

1% Blocking Buffer in Maleic Acid Buffer

**5) BUFFER 3**

0.1M Tris-HCl, 0.1M NaCl, 50mM MgCl2; pH to 9.5

**7)** **Random Primed DNA Labeling Kit** (cat.no. 11004760001, Roche, Switzerland)

**8) DIG Nucleic Acid Detection Kit** (cat.no. 11175041910, Roche, Switzerland)
